# Topology Switching in Polymetallic Fragments Governed by Metal Encapsulation

**DOI:** 10.1021/jacs.6c06300

**Published:** 2026-06-25

**Authors:** Niklas Geue, Kim Greis, Hari R. Newnham, Grigore A. Timco, Richard E. P. Winpenny, Perdita E. Barran

**Affiliations:** † Institute of Chemistry and Biochemistry, Freie Universität Berlin, Altensteinstraße 23a, Berlin 14195, Germany; ‡ Michael Barber Centre for Collaborative Mass Spectrometry, Manchester Institute of Biotechnology, Department of Chemistry, 5292 The University of Manchester, 131 Princess Street, Manchester M1 7DN, U.K.; § Laboratory for Organic Chemistry, Department of Chemistry and Applied Biosciences, ETH Zurich, Zurich CH-8093, Switzerland; ∥ Department of Chemistry, The University of Manchester, Oxford Road, Manchester M13 9PL, U.K.

## Abstract

Metallosupramolecular assemblies are promising platforms for selective extraction, catalysis, and molecular recognition, yet their solution-phase synthesis often relies on complex self-assembly pathways that can be difficult to control. Here, using ion mobility mass spectrometry supported by density functional theory calculations, we introduce a gas-phase approach for directing topology in polymetallic complexes based on the encapsulation of coordination metal cations within a homometallic {Cr_8_} ring. Upon inclusion, the resulting host–guest complexes undergo collision-induced dissociation to produce distinct polymetallic fragments whose topologies, ring, host–guest complex or open chain, depend on the identity of the guest cation. This topology switching enables the targeted synthesis of new polymetallic rings, host–guest complexes and open chains in vacuo and provides insight into the stoichiometric and conformational preferences of such assemblies.

## Graphical abstract

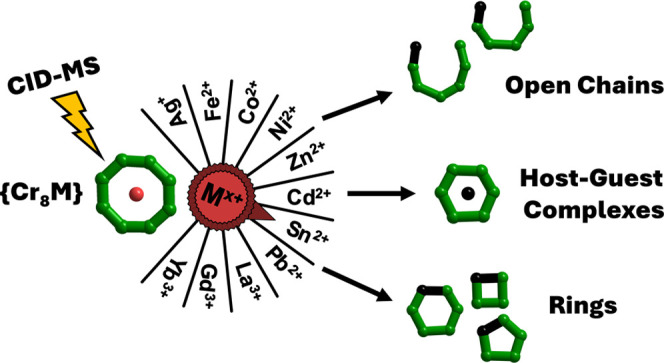

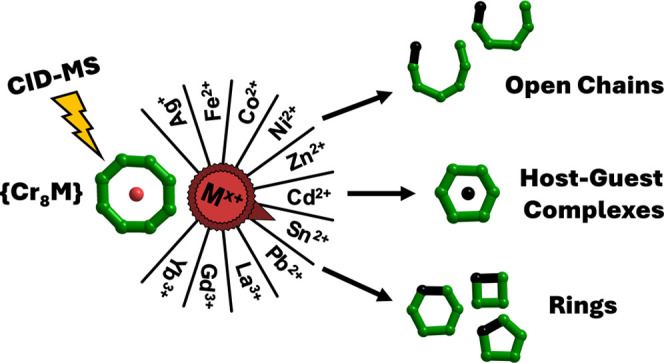

## Introduction

The design and application of metallosupramolecular complexes has attracted substantial interest over the past decades, underpinning their potential in extraction, catalysis, molecular delivery, and even as lithographic resists.^1–4^ However, the synthesis of polymetallic supramolecules typically relies on self-assembly processes that are challenging to control due to the number and complexity of the constituent building blocks. Gaining a deeper understanding of their self-assembly preferences, together with the ability to deliberately direct the formation of specific architectures, represents a critical step toward exploiting the functional potential of such polymetallic systems.^5^

Mass spectrometry (MS) is an ideal tool for investigating supramolecules, offering a unique perspective by probing isolated species in the absence of solvent and counterions.^6–12^ Particularly powerful is its combination with collision-induced dissociation (CID) and ion mobility (IM), which provide complementary dimensions of structural information.^10^ In CID, ions are accelerated into a collision cell filled with an inert gas, inducing structural rearrangements that often lead to fragmentation. The masses of the resulting product ions reveal insights into precursor stability and connectivity. IM separates ions based on their mobility in a buffer gas under low-energy conditions that typically do not induce structural changes. In the simplest form of IM, interactions with the drift gas impede ion motion, with large or extended structures experiencing more interactions than small, compact species. The resulting differences in arrival times provide information on the size and shape of an ion and can be converted into instrument-independent collision cross section (CCS) values.^13–15^

Metallosupramolecular complexes have been studied by several groups using CID and IM-MS.^10^ For example, Li et al. separated macrocyclic precursor ions from the isomeric linear fragment after the collisional activation of a terpyridine-based hexameric Cd-complex.^16^ Peris and co-workers studied the encapsulation of fullerenes and polycyclic aromatic hydrocarbons in metallosquares, showing how IM-MS and density functional theory (DFT) can unravel guest-induced distortions.^17^ More recently, Pfrunder et al. showed that different diastereomers of M_4_L_6_ coordination cages can be resolved with high-resolution IM-MS, demonstrating its potential for the routine analysis of such complexes.^18^

Our group previously used the combination of CID, IM-MS and DFT to unravel the disassembly of Cr^III^-based polymetallic rings and related rotaxanes. We demonstrated how changes in the metal and ligand composition as well as rotaxane stopper groups influence the stability and structural landscape of such complexes.^19–22^ We further showed the formation of polymetallic rings based on the collisional dissociation of larger polymetallic precursors, and developed a workflow to distinguish open and closed polymetallic species, based on their packing density as measured by IM-MS.^23^ When combining IM-MS with scanning tunnelling microscopy via ion soft-landing, we unravelled the self-assembly, rearrangement and disassembly pathways of {Cr_6_}_*n*_ oligomers, highlighting the rich assembly chemistry of such polymetallic systems in vacuo and on surfaces.^5^

Host–guest complex formation is driven by the enthalpic stabilization that accompanies the encapsulation of a guest within a host, enabling a range of applications in sensing, separation, and delivery.^24–26^ Designing hosts and frameworks that can encapsulate heavy metal ions holds promise for the detection and analysis of environmental contaminants.^27–30^ The polymetallic ring [Cr_8_F_8_(O_2_CtBu)_16_], denoted as **1** (Figure 1), was previously predicted to encapsulate suitably sized electropositive guests,^31^ and was later shown to form host–guest complexes with CO_2_,^32^ SO_2_ and H_2_S,^33^ halogens,^34^ as well as alkali metals.^35^

**Figure 1:**
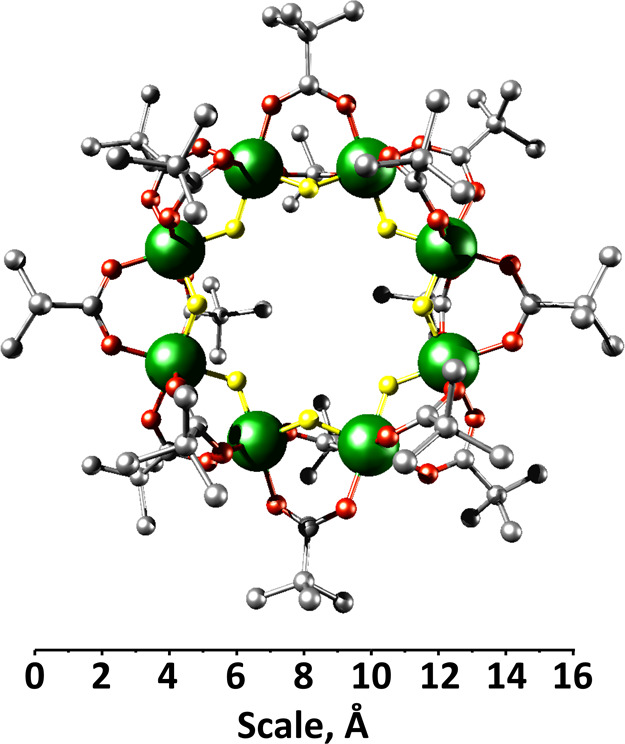
Schematic structure of **1** with a scale in Å (chromium: dark green; fluorine: yellow; oxygen: red; carbon: gray). Hydrogen atoms are omitted for clarity. Adapted from ref 35, Copyright © 2023 The Authors. Published by American Chemical Society.

These findings motivated us to investigate the ability of **1** to bind to heavier metal cations. To probe their structure, stability, and binding preferences, we employed IM-MS and CID. These techniques have been widely applied to characterize host–guest systems, with a key advantage being *m*/*z*-selective ion isolation, which is particularly valuable for hosts such as **1** that can encapsulate guests spanning a wide range of mass.^36–39^

Here, we show the encapsulation of various d- and f-metal cations into the octametallic {Cr_8_} metallocrown **1**, resulting in nonametallic {Cr_8_M} host–guest complexes. These assemblies serve as precursors for the formation of smaller {Cr_*x*_M_*y*_} polymetallic complexes via CID, monitored by IM-MS and supported by DFT. The resulting fragments commonly show the involvement of the guest M in the ring scaffold, enabling the gas-phase formation of heterometallic complexes with distinct topologies (open chain, host–guest complex, or ring), governed by the choice of the guest metal cation (Figure 2).

**Figure 2:**
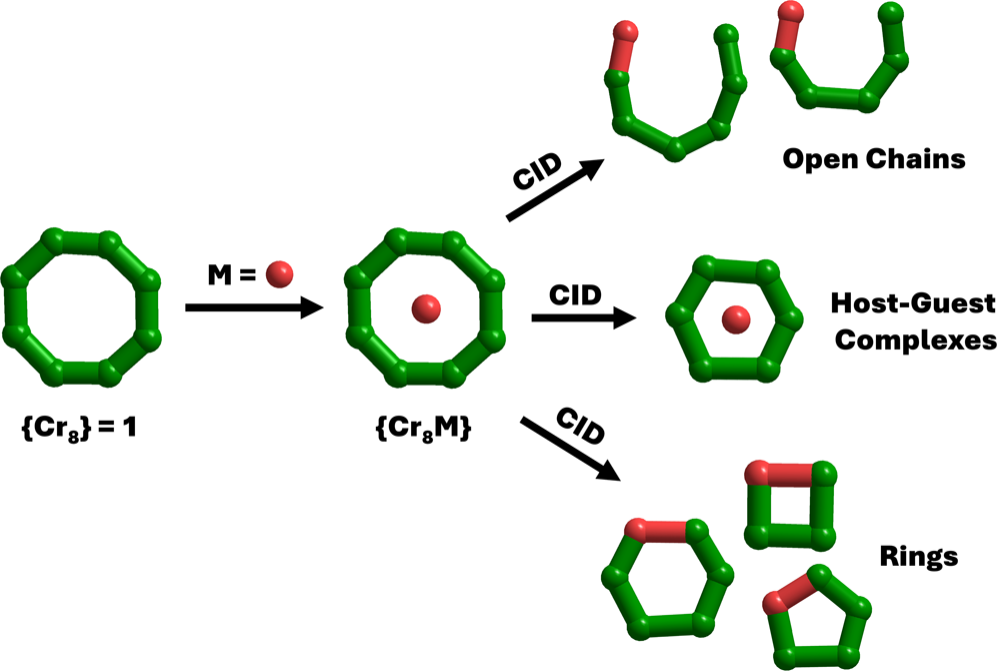
Schematic overview of this work. Encapsulation of different M^*x*+^ in **1** lead to nonametallic {Cr_8_M} host–guest complexes, which are then fragmented by CID. This results in smaller heterometallic stoichiometries that can be host–guest complexes, open chains or rings.

## Experimental Section

### Synthesis, Sample Preparation and Materials

Powders of AgNO_3_, FeCl_2_·4H_2_O, Co­(CH_3_COO)_2_, Ni­(NO_3_)_2_·6H_2_O, Cu­(CH_3_COO)_2_, ZnCl_2_, Cd­(NO_3_)_2_·4H_2_O, SnCl_2_·2H_2_O, Pb­(NO_3_)_2_, Al­(NO_3_)_3_·9H_2_O, Ga­(NO_3_)_3_ hydrate, Y­(CH_3_COO)_3_ hydrate, RuCl_3_ hydrate, InCl_3_ hydrate, Sb­(CH_3_COO)_3_, La­(NO_3_)_3_·9H_2_O, Gd­(NO_3_)_3_·6H_2_O and YbCl_3_·5H_2_O were used. All reagents and solvents were purchased from Sigma-Aldrich, Alfa, Fisher Scientific or Fluorochem and used without further purification.

**1** was prepared according to a previously published route^32^ and was typically transferred to the gas phase from 200 μM solutions in 4:1 toluene/methanol with concentrations of 500 μM or 2500 μM of the respective metal cation. The appearance of the host–guest complex ions was not found to correlate with concentration of the metal cation, however, changed the abundance relative to [**1** + Na]^+^ and related alkali metal adducts.

### IM-MS and Data Processing

The sample solutions were ionized with a nanoelectrospray ionization (nanoESI) source and sprayed from borosilicate glass capillaries (World Precision Instruments, Stevenage, UK), which were pulled on a Flaming/Brown P-2000 laser puller (Sutter Instrument Company, Novato, CA, US). A potential of 1.5–2.5 kV was applied through a platinum wire (Diameter 0.125 mm, Goodfellow, Huntingdon, UK) inserted into the nanoESI capillaries. The source temperature was 23 °C.

IM-MS experiments were performed on a Select Series Cyclic IMS (Waters Corporation).^40^ After the transfer to the gas phase (Cone Voltage: 20 V, Source Offset: 10 V), ions of interest were isolated by a quadrupole mass filter when appropriate, activated in a trap cell at user-defined energies where appropriate (Trap Bias: 2 V, Voltage: 0–200 V) and subsequently injected into the cyclic ion mobility drift ring. In this region, ions were separated by using a nonuniform electric field under a constant nitrogen pressure. Traveling waves (TW, Height: 16–22 V) pushed the ions through the cyclic drift region. Ions traveled one pass in the cyclic drift ring (“single path”, separation time: 2 ms) and were subsequently transferred (transfer energy: 2–15 V) to a time-of-flight mass analyzer. Details of traveling-wave ion mobility spectrometry (TWIMS)^41–43^ and the instrument design^40^ can be found elsewhere. More detailed experimental parameters of this study can be found in the Supplementary Dataset.

Calibration for traveling-wave IM-MS data of polymetallic ions is challenging, due to the lack of easily available calibrants with similar mass, charge and properties.^10^ Experimentally obtained arrival time distributions were converted to nitrogen collisional cross sections (^TW^CCS_N2_, TW = “travelling waves”) and their distributions via two different calibration procedures. All singly charged ions were calibrated with Agilent tune mix^44^ using a power-law approach.^45^ All doubly and triply charged ions were calibrated with Agilent tune mix^44^ and polyalanine^46^ using the state-of-the-art blend function approach.^47^ This was done to keep the data for singly charged ion calibration consistent with our previous works,^19,23,35,48^ where the ^TW^CCS_N2_ values were derived with the same procedure. For higher charged ions, the calibration with singly charged Agilent tune mix ions is not as suitable (particularly for the triply charged ions), which is why polyalanine and the more recently developed blend function approach were applied.

### Density Functional Theory and Collision Cross Section Calculations

The geometries of host–guest complexes and derived homo- and heterometallic fragments were optimized using the composite DFT method *r* ^2^scan-3c,^49^ as implemented in ORCA 6.0.1.^50^ In some difficult to optimize cases, structures were preoptimized using GFN2-xTB as implemented in the xtb package (version 6.5.1).^51^ For the fragments, chemical intuition guided the generation of candidate structures, and CREST was in addition used to sample the conformational space of the homometallic ions [Cr_2_Piv_5_]^+^ and [Cr_3_F_2_Piv_6_]^+^.^52^ Partial charges of all optimized geometries were calculated using the CHELPG scheme at the same level of theory.^53^ All polymetallic ions were assumed to be high-spin complexes because fluorides and carboxylates are weak-field ligands. For the case of [**1** + Fe]^2+^, the low-spin complex yielded the same ^TH^CCS_N2_ value as the high-spin complex within the error of the prediction, however the low-spin complex is 214 kJ mol^–1^ higher in energy (Supplementary Dataset). Hence, all complexes were modeled as high-spin complexes and an impact of the spin state on the ^TH^CCS_N2_ values is likely negligible.

Theoretical CCS values in nitrogen gas (^TH^CCS_N2_, TH: theoretical) were obtained from the software IMoS by using the trajectory method in nitrogen gas including quadrupole potential (temperature = 298 K).^54^

## Results and Discussion

The homometallic ring **1** is based on an octagon of Cr^III^ centers, and each edge of the octagon is bridged by one fluoride ligand on the inside, and two pivalates (^−^O_2_C^*t*^Bu = Piv^–^) on the outside (Figure 1).^55^ The two sides of the ring are not identical in the crystal structures, with a minimum entry distance of the cavity of ca. 4.0 Å on one side and 7.2 Å on the other side.^31^ To date, reported host–guest complexes of **1** have been limited to CO_2_, SO_2_, H_2_S, halogens and alkali metal cations, with no examples involving multiply charged or heavy metal ions.^32–35^ We hypothesized that **1** has the right cavity size to sequestrate a range of d- and f-metals, and we explored the singly charged Ag^+^, the doubly charged Fe^2+^, Co^2+^, Ni^2+^, Cu^2+^, Zn^2+^, Cd^2+^, Sn^2+^, Pb^2+^, and the triply charged Al^3+^, Ga^3+^, Y^3+^, Ru^3+^, In^3+^, Sb^3+^, La^3+^, Gd^3+^ and Yb^3+^.

**1** was transferred to the gas-phase from solutions of the above-mentioned cations in toluene/methanol. The mass spectra reveal a range of species, including the nonametallic ions [**1** + M]^*x*+^ and their water adducts [**1** + M + *y*H_2_O]^*x*+^ (*y* = 1, 2) for M = Ag^+^, Fe^2+^, Co^2+^, Ni^2+^, Cu^2+^, Zn^2+^, Cd^2+^, Sn^2+^, Pb^2+^, La^3+^, Gd^3+^ and Yb^3+^ (Figure 3a for M = Fe^2+^ and Figures S1–S11 for other M^*x*+^). Ions were identified through comparisons of their isotopic patterns with those predicted, and the agreement found was typically excellent (Figure 3a Inset for [**1** + Fe + H_2_O]^2+^). The triply charged cations Al^3+^, Ga^3+^, Y^3+^, Ru^3+^, In^3+^, and Sb^3+^ did not yield [**1** + M]^3+^ or their water adducts [**1** + M + *y*H_2_O]^3+^ (Figures S12–S17), however, some stoichiometries with additionally bound counterions were found (Figure S16 for M = In^3+^). These data suggest that lower charged ions (*x* = +1, +2) are preferred for encapsulation in **1**, whereas for the triply charged metal cations inclusion only occurs for the larger lanthanides (La^3+^, Gd^3+^ and Yb^3+^; Figures S9–S11). The preference for lower oxidation state species can be attributed to stronger destabilizing interactions of M^3+^ with the fluorides in the cavity of **1**, when compared to M^2+^ and M^+^. The size trend further agrees well with our previous data for the alkali metal complexes of **1**, for which we found that the stability order Cs^+^ > K^+^ > Na^+^ depends on the size of the guest cation.^35^

**Figure 3:**
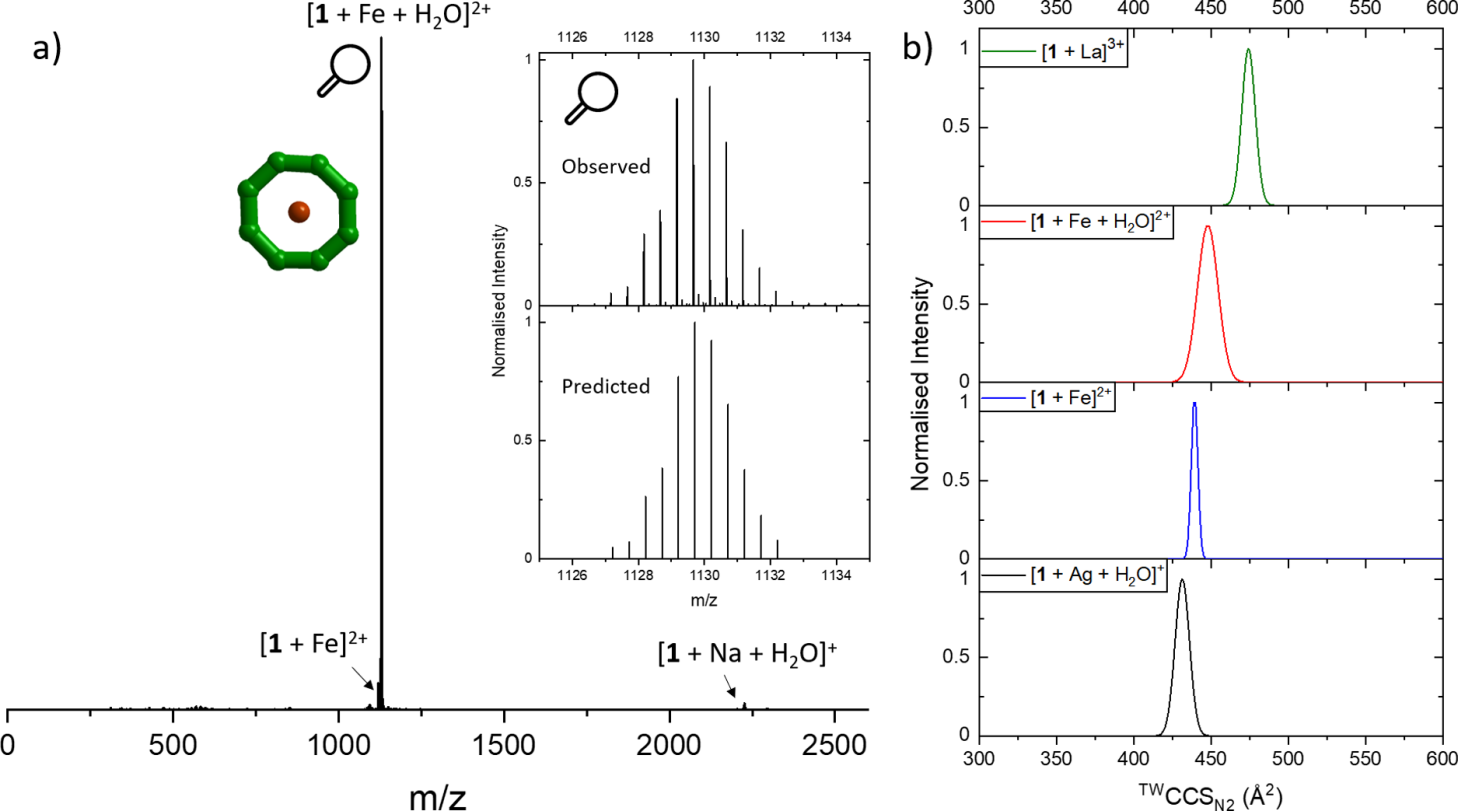
(a) Mass spectrum of 200 μM **1** in 2.5 mM FeCl_2_·4H_2_O dissolved in 4:1 toluene/methanol. [**1** + Fe + H_2_O]^2+^ was found as the base peak, along with small amounts of [**1** + Fe]^2+^ and [**1** + Na + H_2_O]^+^. Inset: Comparison between experimental and observed isotopic distribution for [**1** + Fe + H_2_O]^2+^. (b) ^TW^CCS_N2_ distributions of [**1** + Ag]^+^, [**1** + Fe]^2+^, [**1** + Fe + H_2_O]^2+^ and [**1** + La]^3+^. A ca. 10% increase in ^TW^CCS_N2_ was observed from singly to triply charged ions, along with a slight increase from [**1** + Fe]^2+^ to the water adduct [**1** + Fe + H_2_O]^2+^.

The relative abundances of the host–guest complexes [**1** + M]^*x*+^ and their water adducts differ depending on the encapsulated metal cation (see Supporting Information for details). IM-MS was used to measure CCS_N2_ values of pure host–guest complexes [**1** + M]^*x*+^ and their monowater adduct [**1** + M + H_2_O]^*x*+^ (CCS_N2_ = CCS values in nitrogen gas, Table S1). The CCS_N2_ value of the singly charged [**1** + Ag]^+^ agrees well with previously published values for host–guest complexes of the same family, suggesting inclusion of Ag^+^ in **1**.^19,35^ We observed a ca. 10% increase of CCS_N2_ from [**1** + Ag + H_2_O]^+^ to the triply charged ions, with the doubly charged complexes exhibiting CCS_N2_ values in between (Figure 3b for CCS_N2_ distributions). This trend is known for a range of synthetic molecules and likely corresponds to stronger Coulombic repulsions in higher charged ions, which extend the structure and in turn lead to higher CCS_N2_ values.^5,10^ This, and the comparable high energies of fragmentation when subjected to CID (see further below), strongly suggest the encapsulation of M^*x*+^ and H_2_O in **1**. We also observe a small increase in CCS_N2_ for each host–guest complex when water is included (Figure 3b), which is in the same magnitude as for [**1** + Na]^+^ and [**1** + Na + H_2_O]^+^ in our previous work, where DFT supported the simultaneous inclusion of Na^+^ and H_2_O in **1**.^35^

We used DFT calculations to confirm the encapsulation of the metal cations and water in **1**. The geometries of the experimentally observed host–guest complexes and their corresponding water adducts were optimized (Figures S18–S25 for [**1** + M]^*x*+^ and Figures S26–S35 for [**1** + M + H_2_O]^*x*+^) along with the [**1** + M]^3+^ species that were not found experimentally (Figures S36–S41). The relative binding energies of all computed [**1** + M]^*x*+^ ions were quantified, showing an expected increase in stability for higher oxidation states (Table S2). Among the triply charged ions [**1** + M]^3+^, the largest binding energies were found for the complexes not observed experimentally, which warrants further investigations. The metal ions M^*x*+^ showed different locations within the cavity of **1** depending on their ionic radii (Table S3 for M-F distances in observed [**1** + M]^*x*+^). While the smaller ions Fe^2+^, Co^2+^, Ni^2+^, and Zn^2+^ are located at the edge of the cavity with the smallest M-F distances lying between 1.9–2.0 Å, the larger metals Cd^2+^, Sn^2+^, and Pb^2+^ show minimum M-F distances of 2.3–2.5 Å and are located closer to the center. This effect culminates in the triply charged [**1** + La]^3+^, where the large La^3+^ fully resides in the center of **1** and even induces a distortion of the {Cr_8_} ring toward an ellipsoidal structure (Figure S25). For the DFT structures of the nonobserved complexes, similar effects were found. Ga^3+^, Sb^3+^ and particularly Al^3+^ are located at the edges of the cavity (Table S4 for M-F distances in not observed [**1** + M]^3+^ species), whereas In^3+^ is positioned more central. Y^3+^ and Ru^3+^ induce distortions to the ring structure, similarly to La^3+^ (Figures S38, S39). CCS_N2_ values were simulated for the ions [**1** + M]^*x*+^ and [**1** + M + H_2_O]^*x*+^ that were observed experimentally, and the trajectory method of IMoS was used (Table S1).^54^ The obtained values differ by less than 10% compared to experiment, which is comparable to a systematic discrepancy previously observed for Cr^III^-based polymetallic compounds of similar masses.^19^ This suggests that M^*x*+^ and H_2_O are encapsulated in **1**, which was further supported by the potential energy differences between the DFT optimized structures of [**1** + M]^*x*+^ and [**1** + M + H_2_O]^*x*+^, showing that water inclusion in all cases stabilizes the host–guest complexes (Supplementary Dataset).

We sought to exploit host–guest complexes as precursors for the gas-phase synthesis of smaller polymetallic rings via CID. Previously, using a combination of IM-MS and DFT, we demonstrated that larger polymetallic precursor ions from the same family fragment into smaller ring structures.^19,23,48^ These rearrangements provide access to polymetallic rings that are not readily obtainable in the bulk phase.^23^ However, this strategy relies on the successful synthesis of suitable precursor complexes in solution, which can be difficult to predict and control. We therefore hypothesized that incorporating metal cations into **1** would generate smaller, heterometallic ring fragments, providing a more controlled formation route, given that the identity of the cation M^*x*+^ can be readily varied.

We subjected the host–guest complexes [**1** + M]^*x*+^ and/or their single water adducts [**1** + M + H_2_O]^*x*+^ to CID, depending on their abundance in the mass spectrum, which resulted in a range of homometallic {Cr_a_} and heterometallic {Cr_a_M} fragments with varying ligand stoichiometries. The fragment ions produced were dependent on M as well as the user-defined collision energy. For the water adducts [**1** + M + H_2_O]^*x*+^, the loss of water is the first fragmentation step at comparably low collision energies (Figure S42 for M = Fe^2+^). The next steps, which are in each case highly similar to the first dissociation step of the pure host–guest complexes [**1** + M]^*x*+^, vary from metal to metal.

The complex [**1** + Ag]^+^ follows the consecutive loss of Cr centers along with anionic ligands as the main dissociation channels (Figure S43), similar to the alkali metal adducts [**1** + A]^+^ (A = Na^+^, K^+^, Cs^+^) investigated in our previous work.^35^ The loss of the Ag center, also along with ligands, is observed as a minor pathway in the second step. In contrast to the monovalent metals, the complexes of the divalent metals M = Fe^2+^, Co^2+^, Ni^2+^, Zn^2+^, Cd^2+^, Sn^2+^, and Pb^2+^ follow various fragmentation channels simultaneously, with heterometallic fragments dominating in the low *m*/*z*-region and homometallic ions in the high *m*/*z*-region (Figure 4a for M = Fe^2+^). Due to the possibility of secondary fragmentation, it is difficult to assign the precise disassembly pathways, however, it appears that multiple channels occur at similar energies. This is in contrast to the previously studied, stepwise disassembly of singly charged polymetallic host–guest complexes and (pseudo)­rotaxanes, and we suggest that the multiple fragmentation channels are a consequence of the stronger interactions of the doubly charged guests with the homometallic host **1**.^19,23,35,56^ The host–guest complexes of the larger divalent metals M = Sn^2+^, Pb^2+^ dissociate additionally to {Cr_8_} fragments in low intensities (Figure S44).

**Figure 4:**
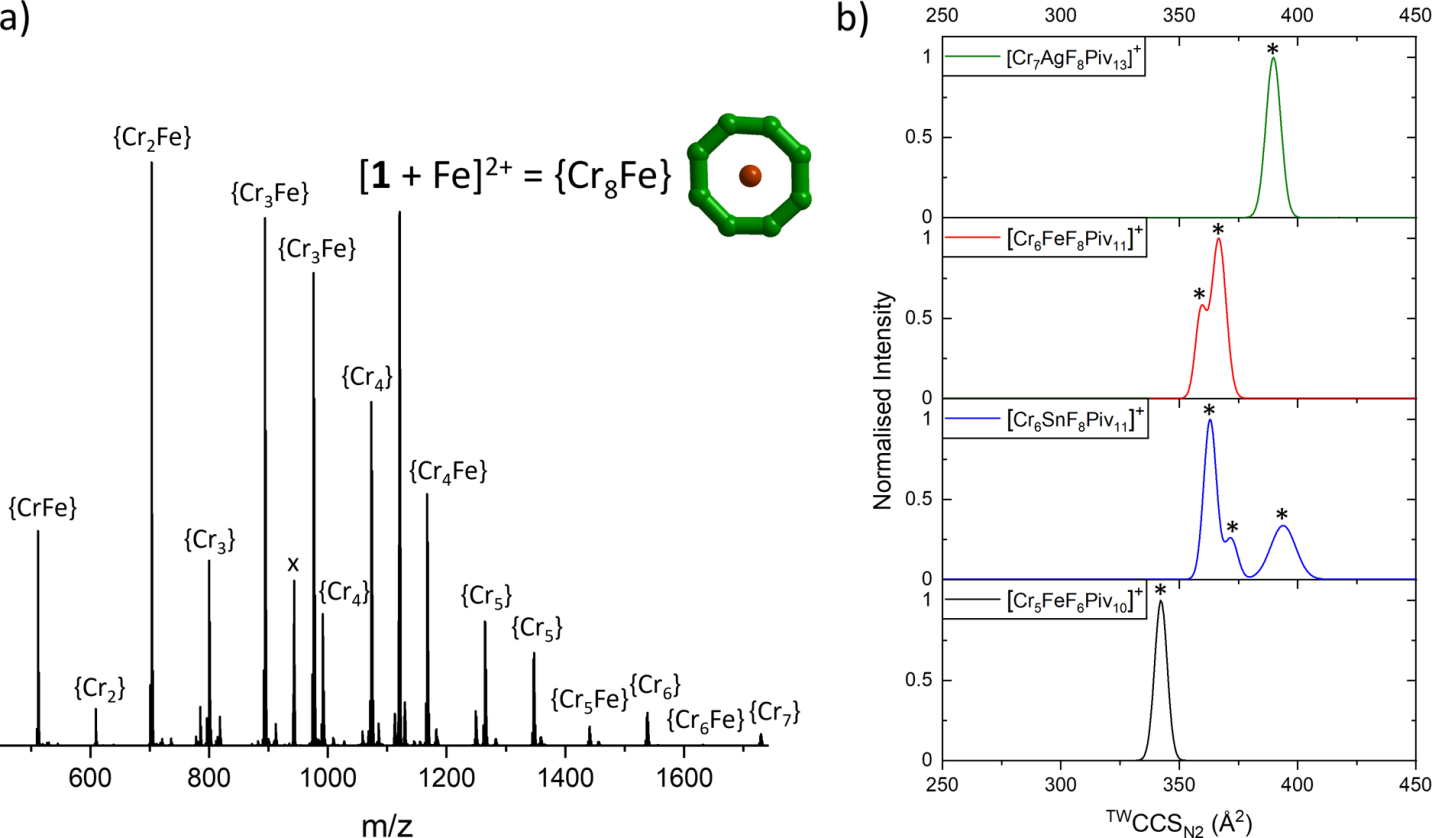
(a) MS^2^ spectrum of [**1** + Fe + H_2_O]^2+^ at *E*_lab_ = 110 eV. Various singly charged fragments were found that include an envelope of homometallic and heterometallic fragment stoichiometries. All fragment ions also possess an appropriate number of anionic F^–^ and Piv^–^ ligands, which are not included in the labels for clarity. The only doubly charged species labeled in the spectrum as “*x*” is [Cr_7_FeF_8_Piv_13_]^2+^. Small fragments are mostly heterometallic, whereas the larger fragments are primarily homometallic. While the ion stoichiometries of these two groups are complementary, their intensities are not, which suggests the occurrence of secondary fragmentation. (b) ^TW^CCS_N2_ distributions of selected fragment ions. Each distribution was fitted with several Gaussians each indicated with a “*”. Data was acquired at *E*_lab_ = 150 eV for [Cr_7_AgF_8_Piv_13_]^+^ and *E*_lab_ = 100 eV for the other three plots.

The exception for the doubly charged metals is Cu^2+^, as the prevalent fragmentation channel of [**1** + Cu]^2+^ involves the loss of CuF_2_ leading to the homometallic fragment [Cr_8_F_6_Piv_16_]^2+^. We attribute this deviation to the Jahn–Teller effect, as previously observed by us for {Cr_7_M} rings,^19,57^ and as a consequence no heterometallic {Cr_a_Cu} fragment ions were found with notable intensities (Figure S45). For the host–guest complexes involving the lanthanides M = La^3+^, Gd^3+^ and Yb^3+^, fragmentation of [**1** + Ln]^3+^ proceeds predominantly to ions of the composition {Cr_6_Ln}^2+^ and {Cr_2_}^+^ as well as {Cr_5_Ln}^2+^ and {Cr_3_}^+^ (Figure S46). Here, larger heterometallic and smaller homometallic fragments are preferred, whereas smaller heterometallic and larger homometallic fragments were found for the fragmentation of the doubly charged host–guest complexes (Figure 4a for M = Fe^2+^). The reason for this different behavior is possibly the distortion of **1** in [**1** + Ln]^3+^ as well as the central position of Ln^3+^, making the attachment to larger homometallic chains easier (Figure S25 for M = La^3+^). Overall, we consistently observe various heterometallic fragment stoichiometries when dissociating each [**1** + M]^*x*+^ or [**1** + M + H_2_O]^*x*+^ (Figure 4a for M = Fe^2+^), except for the case of M = Cu^2+^. Homometallic and heterometallic fragment stoichiometries of the different disassembly processes, ranging from octametallic to dimetallic ions, are noted in Tables S5 and S6.

Arrival time distributions (ATDs) were acquired and extracted for eight homometallic and 67 heterometallic fragments, differing in M and {Cr_a_M} stoichiometry. Ions sharing the same {Cr_a_M} stoichiometry varied in ligand composition and hence mass. For each stoichiometry, the dominant ion(s) was characterized. CCS_N2_ distributions and values were obtained via established calibration procedures.^45,47^ The majority of CCS_N2_ distributions present unimodal, indicating one conformation, however, some homometallic (Table S5) and heterometallic stoichiometries (Figure 4b, Table S6) show multiple conformations, similar to those previously found in polymetallic fragments of this family.^19^ For example, [Cr_7_AgF_8_Piv_13_]^+^ and [Cr_5_FeF_6_Piv_10_]^+^ exhibit one distribution each, whereas the fragments [Cr_6_FeF_8_Piv_11_]^+^ and [Cr_6_SnF_8_Piv_11_]^+^ show two and three peaks, respectively, despite being isostructural to each other (Figure 4b).

Previously, we developed a workflow to assess the topology of polymetallic compounds of the same family, which is based on the packing density of the ions.^5,23,48^ When plotting CCS_N2_ vs ion mass, ring fragments demonstrated a linear correlation, whereas the data for open polymetallic compounds or other noncyclic species was located above this line.^23^ We predict that host–guest complexes have a higher packing density than rings and are hence located below the line of ring fragments. To systematically assess the topology of all heterometallic fragments formed, we chose the smallest structures of the homometallic fragments as the reference for rings (Figure 5, black and Table S5), which yielded a convincing linear correlation (*R* ^2^ = 0.9996) and agreed well with the packing densities of other rings previously reported.^23^ We derived CCS_N2_ vs mass plots for the heterometallic fragments and host–guest complexes described in Tables S1 and S6 and compared them to the homometallic trendline. For the fragments derived from {Cr_8_Fe}^2+^, {Cr_8_Sn}^2+^ and {Cr_8_Ag}^+^, the data is shown in Figure 5.

**Figure 5:**
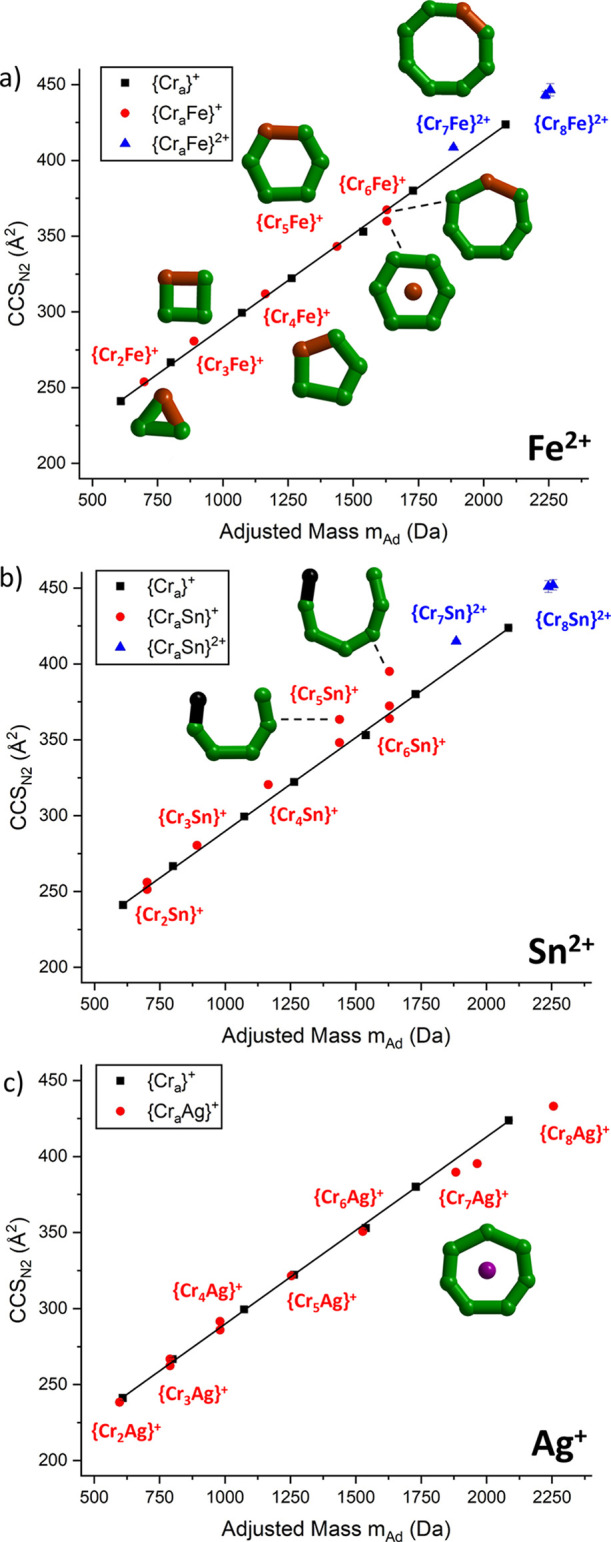
Correlation between CCS_N2_ and adjusted mass (*m*_ad_) for homometallic (+1: black squares, Table S5) and heterometallic ions (+1: red circles; +2: blue triangles; host–guest complexes from Table S1 and fragments from Table S6) upon encapsulation of (a) Fe^2+^, (b) Sn^2+^ and (c) Ag^+^. The mass was adjusted so that every metal center is accounted for with the mass of chromium (52 Da).^23^ The dominant ion of each {Cr_a_M} stoichiometry was chosen, and they can differ in ligand composition and mass. The fragment data for the homometallic {Cr_a_} complexes shows a strong linear correlation, whereas the heterometallic fragment data can be found on, above and below this trend line, suggesting rings, open chains or host–guest complexes, respectively.

The plot for Fe^2+^ suggests two structures for {Cr_6_Fe}^+^, one on the line, assigned as a ring, and one below the line, likely corresponding to a host–guest complex (Figure 5a, Table 1). All smaller stoichiometries exhibit packing densities indicative of rings. In general, CCS_N2_ increases for higher charge states, independently of ion geometry, as observed for the nonametallic host–guest complexes (Table S1). This makes direct assignments of doubly or triply charged fragments in relation to the singly charged homometallic ions challenging. However, the ca. 3% CCS_N2_ deviation of {Cr_7_Fe}^2+^ to the {Cr_a_}^+^ trendline agrees with the 3% CCS_N2_ difference between the host–guest complexes of {Cr_8_Fe}^2+^ and {Cr_8_Ag}^+^, suggesting that this is solely a charge effect (Figure 5a). Hence, {Cr_7_Fe}^2+^ is assigned as a ring. The heterometallic fragments originating from the species {Cr_8_Co}^2+^, {Cr_8_Ni}^2+^, {Cr_8_Zn}^2+^, and {Cr_8_Cd}^2+^ show highly similar behavior as {Cr_8_Fe}^2+^, indicating that all heterometallic fragments of these divalent metals are rings except one structure of {Cr_6_M}^+^ each (Figures S47–S50). Notably, the confidence of the topology assignments is slightly lower for smaller ions due to an increased structural flexibility and less defined topologies.

**Table 1:**
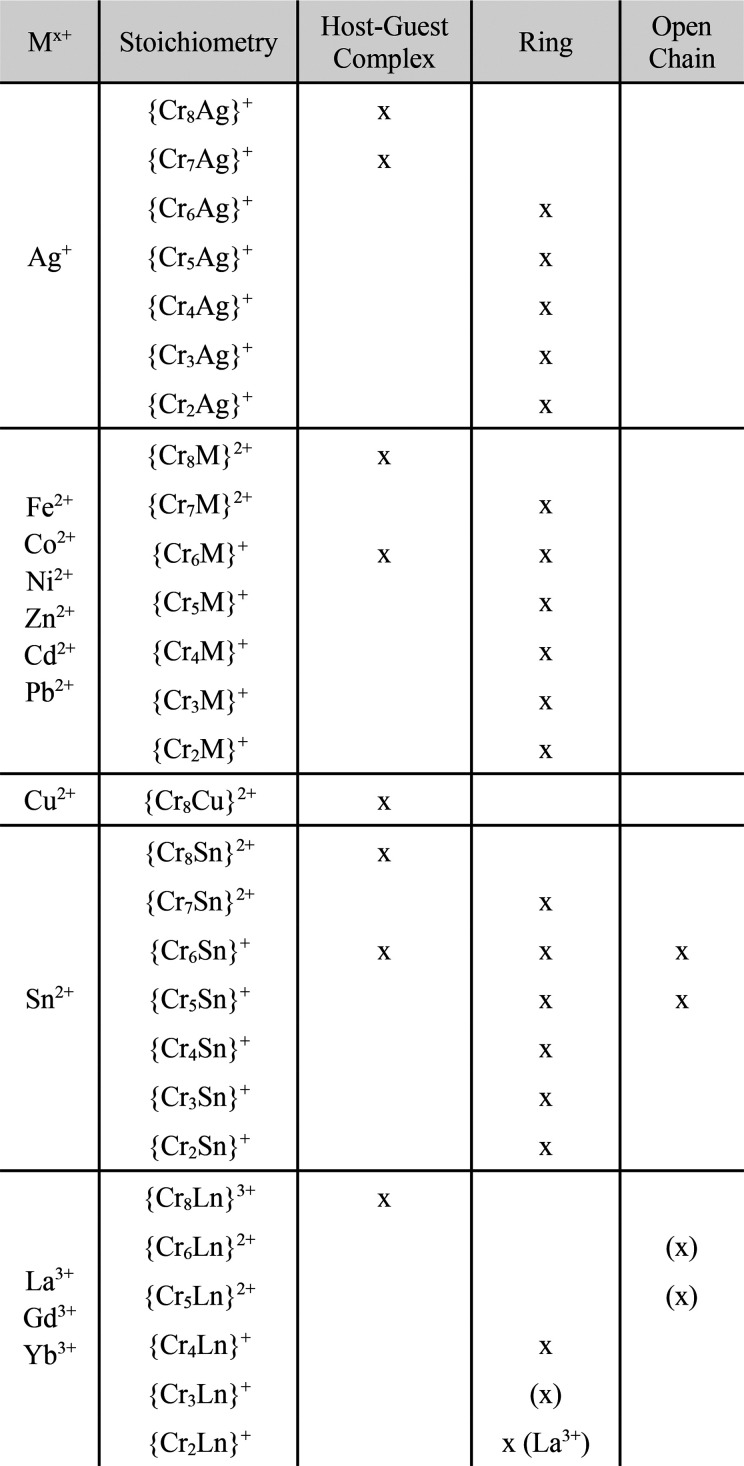
Summary of Observed Heterometallic Precursor and Fragment Ions and Their Topologies^a^

a x denotes that the respective topology was found, whereas (x) indicates tentative assignment.

For {Cr_8_Pb}^2+^ and {Cr_8_Sn}^2+^, a slight increase of CCS_N2_ was found for the obtained fragments (Figures 5b, S51), which is comparable to differences in CCS_N2_ of the host–guest complexes (Table S1) and likely a result of the larger radii of Pb^2+^ and Sn^2+^ compared to the other divalent metal cations, as discussed above.^58^ The stoichiometries {Cr_6_Sn}^+^ and {Cr_5_Sn}^+^ showed an additional structure each, exhibiting a significantly lower packing density than the homometallic trendline (Figure 5b). We assign these ions to open chain structures, as previously found in the fragmentation of {Cr_7_M} rings (Table 1).^19^ The three lanthanide host–guest complexes {Cr_8_Ln}^3+^ (Ln^3+^ = La^3+^, Gd^3+^, Yb^3+^) fragment to {Cr_6_Ln}^2+^ and {Cr_5_Ln}^2+^ species, and these are tentatively assigned to open chain structures (Figures S52–S54). The smaller singly charged fragments agree well with the trendline, except for one structure of {Cr_3_Yb}^+^ and {Cr_3_Gd}^+^, which appear with a higher packing density than a ring (Figures S53, S54). Host–guest complexes are, however, unlikely in these cases due to the size of Ln^3+^ ions, leading to our tentative assignment of a somehow more compact ring topology.

For Ag^+^, the precursor {Cr_8_Ag}^+^ host–guest complex lies below the line, and so does the {Cr_7_Ag}^+^ species formed in the first fragmentation step (Figure 5c). This suggests that the formed {Cr_7_Ag}^+^ ions are also host–guest complexes (Table 1). All smaller {Cr_a_Ag}^+^ fragments are located on or very close to the linear fit, which is indicative of ring structures. Notably, both {Cr_4_Ag}^+^ and {Cr_3_Ag}^+^ show two conformations suggesting a ring each, which could be due to differences in linkage or bridging. This was previously suggested to be a result from distinct rearrangement channels of such polymetallic compounds.^23^

We used DFT calculations to assess ring structures and host–guest complexes of the selected fragment stoichiometries {Cr_7_Ag}^+^, {Cr_7_Cd}^2+^, {Cr_6_Sn}^+^, and {Cr_5_Zn}^+^, with the intention to support the IM-MS-based topology assignments. Cd^2+^ and Zn^2+^ were chosen as the heterometal cations to minimize computational expense, given their closed-shell configurations. As previously observed, the structural landscape of such polymetallic fragments is highly diverse, encompassing numerous connectivities, bridging motifs, and conformations.^19,23^ Reliable modeling of their structures is challenging, and our primary aims were first to assess whether the host–guest complex and ring topologies are in general feasible, and second to support our assignments through comparisons of theoretical CCS_N2_ values with experimental data. Open chain structures were not modeled as they are likely possible for all stoichiometries, due to the absence of steric constraints and a large conformational flexibility.^19^

For the selected fragments, energetic minima were found for both ring and host–guest topologies (Figures S55–S58). CCS_N2_ values were simulated for all modeled complexes (Table S7), and then scaled with a mass-dependent scaling factor. We previously observed that theoretical CCS_N2_ values systematically differ from experimental data for polymetallic compounds of this family,^19^ and for another case of cycloparaphenylenes, we found that this scaling factor can be mass-dependent.^59^ To establish a suitable scaling workflow for the theoretical CCS_N2_ values of the heterometallic fragments, DFT calculations and CCS_N2_ predictions were performed for the homometallic {Cr_a_}^+^ ring structures (Figures S59–S65, Table S8). The resulting linear function between theoretical CCS_N2_ and mass was divided by the linear function between experimental CCS_N2_ and mass, yielding a mass-dependent scaling function (Figure S66). This was then used to scale the theoretical CCS_N2_ values of the heterometallic fragments (Table 2).^19^

**Table 2:** ^TW^CCS_N2_ and Scaled ^TH^CCS_N2_ Values of Selected Fragment Ions Including Experimental and Theoretical Error, Respectively^a^

fragment	mass (Da)	^TW^CCS_N2_ (Å^2^)	experimental assignment	scaled ^TH^CCS_N2_ of the ring (Å^2^)	scaled ^TH^CCS_N2_ of the host–guest complex (Å^2^)
[Cr_7_AgF_8_Piv_13_]^+^	1938.3	389.6 ± 0.1	host–guest complex	399.5 ± 0.8	391.3 ± 1.1
[Cr_7_CdF_8_Piv_13_]^2+^	1943.2	407.1 ± 0.6	ring	412.3 ± 2.2	404.7 ± 1.3
[Cr_6_SnF_8_Piv_11_]^+^	1695.2	363.9 ± 0.1	host–guest complex	373.3 ± 1.2	366.2 ± 1.8
		372.2 ± 0.1	ring		
[Cr_5_ZnF_6_Piv_10_]^+^	1450.2	342.3 ± 0.1	ring	342.9 ± 1.1	337.9 ± 0.9

a ^TH^CCS_N2_ values and errors were scaled with a mass-dependent scaling factor (Figure S66). Unscaled ^TH^CCS_N2_ values can be found in Table S7.

For {Cr_7_Ag}^+^, the host–guest complex structure was found to be energetically more stable than the ring (Table S7), and the scaled ^TH^CCS_N2_ value of the host–guest complex agrees well with experiment (Table 2). This supports the formation of a {Cr_7_Ag}^+^ host–guest complex as suggested by IM-MS. The CCS_N2_ comparison for the isostructural {Cr_7_Cd}^2+^ however is not conclusive (Table 2), which could be attributed to a potential charge-dependence of the scaling factor. This cannot be further quantified due to the lack of multiply doubly charged homometallic fragments. In addition, the relative minimum energies favor the ring structure, which contrasts with experimental assignment (Table S7). This is potentially due to a lack of structural and conformational sampling, which is in general challenging for such large polymetallic structures.^19^

For {Cr_6_Sn}^+^, the scaled ^TH^CCS_N2_ values of the modeled host–guest complex and ring agree well with the two smaller experimental structures, as proposed from experiment (Table 2). Energetically, a ring is only slightly more stable than the host–guest complex, which is in favor of both structures coexisting as predicted by IM-MS (Table S7). The experimental CCS_N2_ for {Cr_5_Zn}^+^ agrees very well with the data of the modeled ring structure, which is also energetically preferred over the host–guest complex (Tables 2, S7). This supports the IM-MS-based assignment.

## Discussion

Collision-induced dissociation of {Cr_8_M} host–guest complexes yields smaller host–guest complexes, polymetallic rings, or open chain motifs, with the preferred dissociation pathway governed by the fragment stoichiometry and by the identity of the encapsulated metal ion M. All these processes are resolved by IM-MS and supported by DFT calculations, which together enable the discrimination between the three topological outcomes. We suggest that a combination of thermodynamic stability and kinetic effects, such as isomerization barriers, determine the topology of the fragments, with the size and charge of M strongly influencing the energetics. For all M, the majority of the heterometallic fragments identified were ring structures in which the metal ion M was incorporated into the ring framework. Host–guest complexes were observed only for {Cr_6_M} stoichiometries and for the singly charged {Cr_7_Ag}^+^ species, indicating that low charged metal ions are more likely to remain encapsulated within the homometallic ring upon fragmentation. This behavior, however, is limited to polymetallic assemblies with overall larger stoichiometries due to spatial requirements of M^*x*+^. In contrast, open chain structures were found exclusively for larger metal ions (Sn^2+^, and likely La^3+^, Gd^3+^, and Yb^3+^), again likely due to the greater steric demand of M^*x*+^ (Table 1).

Beyond providing insight into the intrinsic stability and reactivity of these assemblies, these results establish gas-phase fragmentation as a route to well-defined molecular architectures that are directly relevant to synthetic metallosupramolecular chemistry. Notably, this approach represents a substantial advance over our previous work,^23^ as it does not rely on the solution-phase synthesis of larger precursor ions and is instead highly versatile owing to the broad range of metal ions that can be encapsulated within the homometallic ring **1**. Whether polymetallic rings that do not incorporate Cr^III^ centers exhibit comparable fragmentation behavior including topology switching will be explored in a future study.^60–62^

Gas-phase chemistry can provide valuable insight into bulk-phase reactivity, as stoichiometrically and structurally well-defined fragments observed in the gas phase are often sufficiently stable to be viable targets for condensed-phase synthesis. Accordingly, this approach establishes a predictive platform for the discovery of new bulk-phase polymetallic {Cr_a_M} compounds,^23^ potentially enabling artificial intelligence tools to identify such structures with desirable properties.^10^ Such data sets can be efficiently generated with acquisition times of just a few minutes per metal while requiring only minimal sample quantities. One recent example is the prediction of a stable {Cr_6_Gd_2_} ring that we observed using IM-MS following CID,^23^ which we then synthesized and crystallized outside the mass spectrometer.^63^

A second benefit of gas-phase synthesis is that the generated fragments can be further characterized by gas-phase methods such as spectroscopy^64–66^ or multistage fragmentation studies. A particularly compelling extension for synthetic chemistry is the integration of gas-phase synthesis with ion soft-landing, which we have previously applied to generate {Cr_6_} horseshoe-shaped open chain oligomers of the same polymetallic family.^5^ In this method, *m*/*z*- (and conformationally)^67^ selected/purified ions^68^ are carefully deposited on a surface inserted in the mass spectrometer, which then in turn can be analyzed with conventional characterization techniques ex situ,^69^ or even exploited for on-surface reactions.^70,71^ Although such instrumentation has so far been developed by only a limited number of specialized groups,^69,72,73^ analytical tools including single-molecule microscopy,^74–77^ IR spectroscopy,^78^ and mass spectrometry,^79^ have demonstrated its utility for the characterization of soft-landed species. As this field continues to mature, we expect gas-phase synthesis strategies to become increasingly relevant for the rational design of metallosupramolecules.^10^ However, a central challenge in ion soft-landing is the generation of sufficiently high ion numbers to be of synthetic relevance. For the heterometallic {Cr_a_M} complexes discussed here, this limitation is in some cases compounded by comparatively low relative ion yields (below 1%), which arise from the presence of multiple competing fragmentation pathways (for example in Figure 4a).^80^

## Conclusions

We demonstrate that the homometallic {Cr_8_} ring **1** can encapsulate d- and f-metal cations of different sizes and charges, giving rise to a series of previously unobserved nonametallic host–guest complexes. These species serve as well-defined precursors for dissociation into smaller polymetallic rings, host–guest complexes, or open chain structures, as distinguished by IM-MS and supported by DFT calculations. By appropriate choice of the encapsulated metal cation, this strategy enables access to a diverse range of polymetallic {Cr_a_M} stoichiometries and provides detailed insight into the conformational and stoichiometric preferences of such assemblies.

## Supplementary Material



## Data Availability

The Supplementary Dataset is available on Figshare (10.6084/m9.figshare.31752442.v2) containing the raw data of ion mobility mass spectrometry measurements as well as results of DFT calculations.

## Notes

Mass spectra of **1** in different salt solutions, discussion on water binding in the host–guest complexes of **1**, experimental and theoretical CCS_N2_ values of the host–guest complexes and fragments, DFT optimized structures of the host–guest complexes and fragments as well as their relative energies, M-F distances in the DFT optimized structures of the host–guest complexes, tandem mass spectra of host–guest complexes, plots of CCS_N2_ vs adjusted mass for other heterometallic fragments, derivation of mass-dependent scaling function (PDF)
