# Supplementary material for: Topology Switching in Polymetallic Fragments Governed by Metal Encapsulation
Source: J Am Chem Soc. 2026 Jun 25;148(26):27570–80. doi: 10.1021/jacs.6c06300 (PMC13352637; doi:10.1021/jacs.6c06300)
Supplement: Supplementary file 1 [file ja6c06300_si_001.pdf]

Supporting Information

Topology Switching in Polymetallic Fragments Governed by Metal Encapsulation

Niklas Geue,[1,2,*] Kim Greis,[3] Hari R. Newnham,[2] Grigore A. Timco,[4] Richard E. P. Winpenny[4] and Perdita E. Barran[2,*]

[1]Institute of Chemistry and Biochemistry, Freie Universität Berlin, Altensteinstraße 23a, 14195 Berlin, Germany. [2]Michael Barber Centre for Collaborative Mass Spectrometry, Manchester Institute of Biotechnology, Department of Chemistry, The University of Manchester, 131 Princess Street, Manchester, M1 7DN, UK. [3]Laboratory for Organic Chemistry, Department of Chemistry and Applied Biosciences, ETH Zurich, CH-8093 Zurich, Switzerland. [4]Department of Chemistry, The University of Manchester, Oxford Road, Manchester, M13 9PL, UK.

[*]Corresponding Authors: niklas.geue@fu-berlin.de, perdita.barran@manchester.ac.uk

Table of Contents

Figure S1: Mass spectrum of **1** and $AgNO_3$ 5

Figure S2: Mass spectrum of **1** and $Co(CH_3COO)_2$ 6

Figure S3: Mass spectrum of **1** and $Ni(NO_3)_2 \cdot 6\ H_2O$ 7

Figure S4: Mass spectrum of **1** and $Cu(CH_3COO)_2$ 8

Figure S5: Mass spectrum of **1** and $ZnCl_2$ 9

Figure S6: Mass spectrum of **1** and $Cd(NO_3)_2 \cdot 4\ H_2O$ 10

Figure S7: Mass spectrum of **1** and $SnCl_2 \cdot 2\ H_2O$ 11

Figure S8: Mass spectrum of **1** and $Pb(NO_3)_2$ 12

Figure S9: Mass spectrum of **1** and $La(NO_3)_3 \cdot 9\ H_2O$ 13

Figure S10: Mass spectrum of **1** and $Gd(NO_3)_3 \cdot 6\ H_2O$ 14

Figure S11: Mass spectrum of **1** and $YbCl_3 \cdot 5\ H_2O$ 15

Figure S12: Mass spectrum of **1** and $Al(NO_3)_3 \cdot 9\ H_2O$ 16

Figure S13: Mass spectrum of **1** and $Ga(NO_3)_3$ hydrate 17

Figure S14: Mass spectrum of **1** and $Y(CH_3COO)_3$ hydrate 18

Figure S15: Mass spectrum of **1** and $RuCl_3$ hydrate 19

Figure S16: Mass spectrum of **1** and $InCl_3$ hydrate 20

Figure S17: Mass spectrum of **1** and $Sb(CH_3COO)_3$ 21

Discussion on Water Binding in the Host-Guest Complexes 22

Table S1: $^{TW}CCS_{N2}$ and $^{TH}CCS_{N2}$ values of [**1** + M]$^{x+}$ and [**1** + M + H_2O]$^{x+}$ 23

Figure S18: DFT optimised structure of [**1** + Fe]$^{2+}$ 24

Figure S19: DFT optimised structure of [**1** + Co]$^{2+}$ 25

Figure S20: DFT optimised structure of [**1** + Ni]$^{2+}$ 26

Figure S21: DFT optimised structure of [**1** + Zn]$^{2+}$ 27

Figure S22: DFT optimised structure of [**1** + Cd]$^{2+}$ 28

Figure S23: DFT optimised structure of [**1** + Sn]$^{2+}$ 29

Figure S24: DFT optimised structure of [**1** + Pb]$^{2+}$ 30

Figure S25: DFT optimised structure of [**1** + La]$^{3+}$ 31

Figure S26: DFT optimised structure of [**1** + Ag + H_2O]$^{+}$ 32

Figure S27: DFT optimised structure of [**1** + Fe + H_2O]$^{2+}$ 33

Figure S28: DFT optimised structure of [**1** + Co + H_2O]$^{2+}$ 34

Figure S29: DFT optimised structure of [**1** + Ni + H_2O]$^{2+}$ 35

Figure S30: DFT optimised structure of [**1** + Cu + H_2O]$^{2+}$ 36

Figure S31: DFT optimised structure of [**1** + Zn + H_2O]$^{2+}$ 37

Figure S32: DFT optimised structure of [**1** + Cd + H_2O]$^{2+}$ 38

Figure S33: DFT optimised structure of [**1** + Sn + H_2O]$^{2+}$ 39

Figure S34: DFT optimised structure of [**1** + Pb + H_2O]$^{2+}$ 40
Figure S35: DFT optimised structure of [**1** + La + H_2O]$^{3+}$ 41
Figure S36: DFT optimised structure of [**1** + Al]$^{3+}$ 42
Figure S37: DFT optimised structure of [**1** + Ga]$^{3+}$ 43
Figure S38: DFT optimised structure of [**1** + Y]$^{3+}$ 44
Figure S39: DFT optimised structure of [**1** + Ru]$^{3+}$ 45
Figure S40: DFT optimised structure of [**1** + In]$^{3+}$ 46
Figure S41: DFT optimised structure of [**1** + Sb]$^{3+}$ 47
Table S2: Relative binding energies of M to **1** in the DFT optimised structures of [**1** + M]$^{x+}$ 48
Table S3: Eight M-F distances of the experimentally observed [**1** + M]$^{x+}$ species 49
Table S4: Eight M-F distances of the not experimentally observed [**1** + M]$^{3+}$ species 50
Figure S42: MS^2 spectrum of [**1** + Fe + H_2O]$^{2+}$ at E_{lab} = 60 eV 51
Figure S43: MS^2 spectrum of [**1** + Ag + H_2O]$^{+}$ at E_{lab} = 155 eV 52
Figure S44: Comparison between observed and predicted isotopic distribution of [$Cr_8F_8Piv_{15}$]$^{+}$ 53
Figure S45: MS^2 spectrum of [**1** + Cu + H_2O]$^{2+}$ at E_{lab} = 80 eV 54
Figure S46: MS^2 spectrum of [**1** + La]$^{3+}$ at E_{lab} = 99 eV 55
Table S5: Composition, m/z and $^{TW}CCS_{N2}$ of selected homometallic fragments 56
Table S6: Composition, m/z and $^{TW}CCS_{N2}$ of selected heterometallic fragments 57
Figure S47: Correlation between CCS_{N2} and adjusted mass for {Cr_aCo} ions. 61
Figure S48: Correlation between CCS_{N2} and adjusted mass for {Cr_aNi} ions. 62
Figure S49: Correlation between CCS_{N2} and adjusted mass for {Cr_aZn} ions. 63
Figure S50: Correlation between CCS_{N2} and adjusted mass for {Cr_aCd} ions. 64
Figure S51: Correlation between CCS_{N2} and adjusted mass for {Cr_aPb} ions. 65
Figure S52: Correlation between CCS_{N2} and adjusted mass for {Cr_aLa} ions. 66
Figure S53: Correlation between CCS_{N2} and adjusted mass for {Cr_aGd} ions. 67
Figure S54: Correlation between CCS_{N2} and adjusted mass for {Cr_aYb} ions. 68
Figure S55: DFT optimised structures of fragment [$Cr_7AgF_8Piv_{13}$]$^{+}$ 69
Figure S56: DFT optimised structures of fragment [$Cr_7CdF_8Piv_{13}$]$^{2+}$ 70
Figure S57: DFT optimised structures of fragment [$Cr_6SnF_8Piv_{11}$]$^{+}$ 71
Figure S58: DFT optimised structures of fragment [$Cr_5ZnF_6Piv_{10}$]$^{+}$ 72
Table S7: Unscaled $^{TH}CCS_{N2}$ values of selected fragment ions 73
Figure S59: DFT optimised structures of fragment [Cr_2Piv_5]$^{+}$ 74
Figure S60: DFT optimised structures of fragment [$Cr_3F_2Piv_6$]$^{+}$ 75
Figure S61: DFT optimised structure of fragment [$Cr_4F_3Piv_8$]$^{+}$ 76
Figure S62: DFT optimised structures of fragment [$Cr_5F_5Piv_9$]$^{+}$ 77
Figure S63: DFT optimised structure of fragment [$Cr_6F_6Piv_{11}$]$^{+}$ 78

Figure S64: DFT optimised structures of fragment $[Cr_7F_8Piv_{12}]^+$ 79
Figure S65: DFT optimised structure of fragment $[Cr_8F_8Piv_{15}]^+$ 80
Table S8: $^{TH}CCS_{N2}$ values and relative minimum energies for singly charged homometallic fragments.......... 81
Figure S66: Scaling function plot based on homometallic $\{Cr_a\}^+$ ions.......... 82
References 84

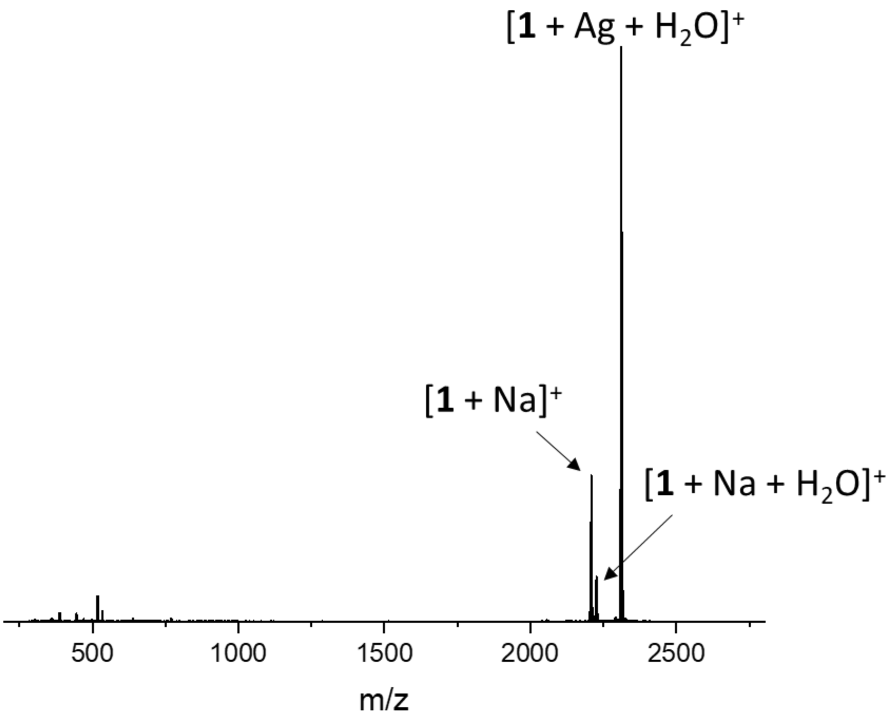

Figure S1: Mass spectrum of **1** and $AgNO_3$ dissolved in 4:1 toluene/methanol. The ion [**1** + Ag + H_2O]$^+$ was found as the main peak.

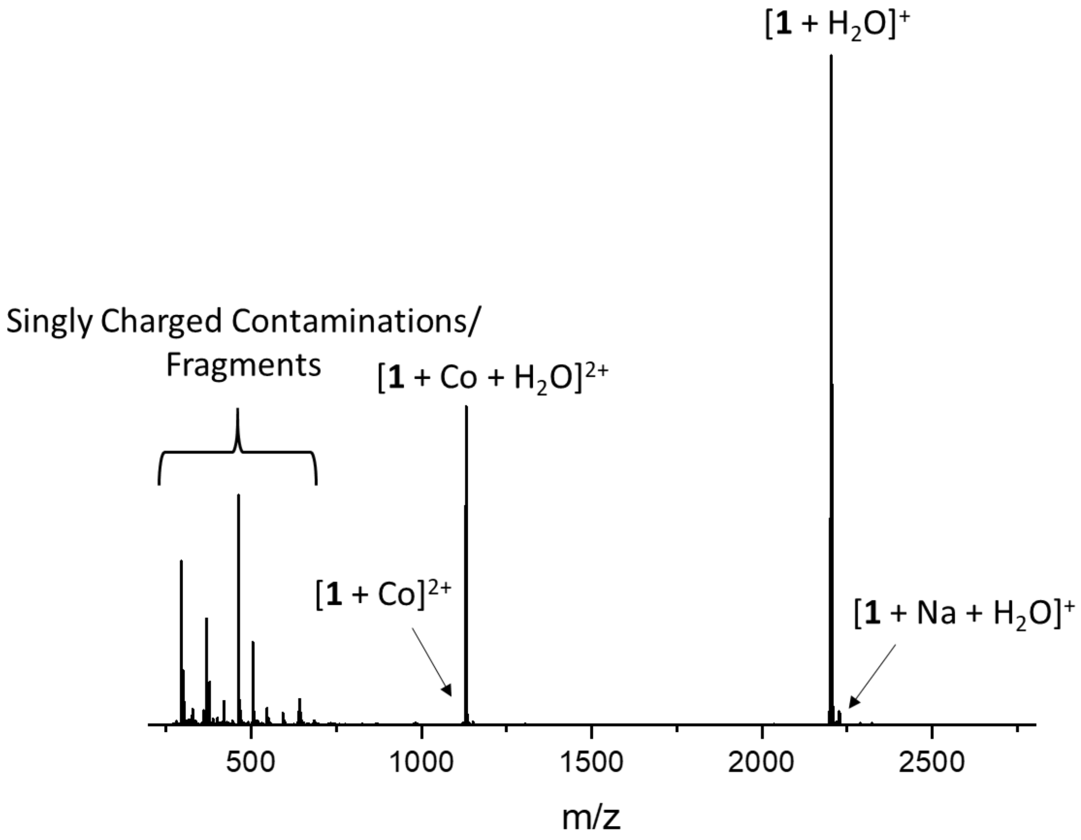

Figure S2: Mass spectrum of **1** and $Co(CH_3COO)_2$ dissolved in 4:1 toluene/methanol. Except for the non-cobalt-containing ion assigned as [**1** + H_2O]$^+$, the water adduct [**1** + Co + H_2O]$^{2+}$ was found as the main peak, with traces of [**1** + Co]$^{2+}$.

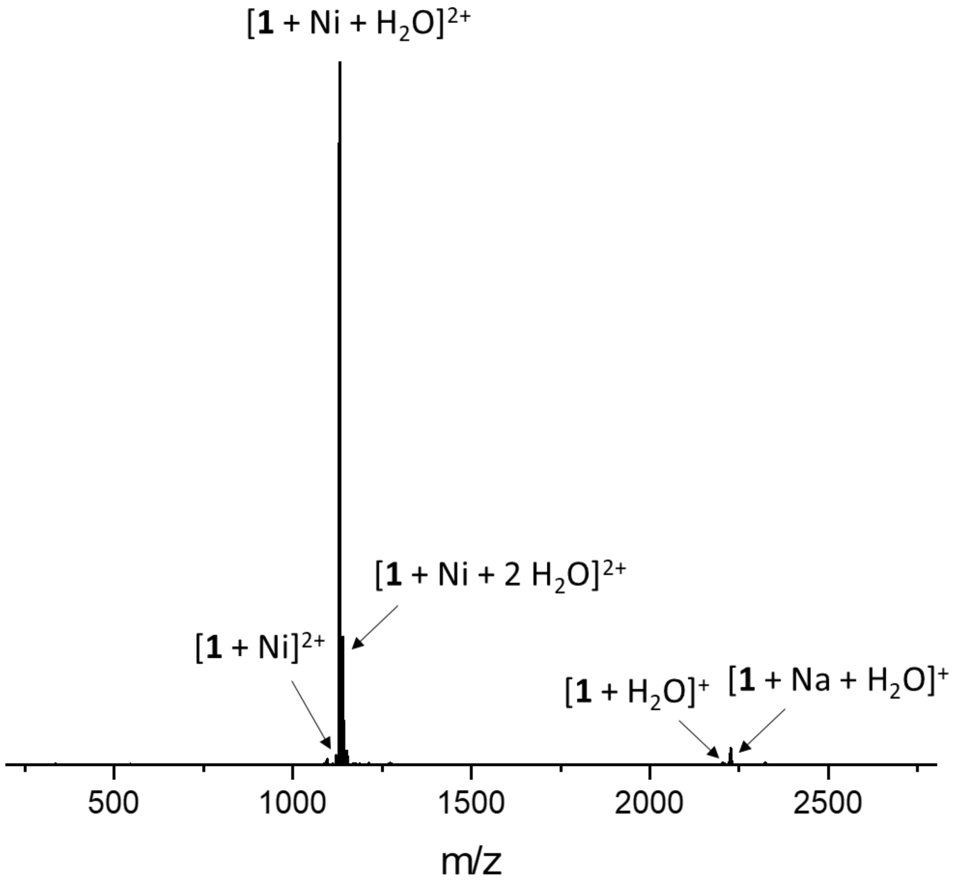

Figure S3: Mass spectrum of **1** and $Ni(NO_3)_2 \cdot 6\ H_2O$ dissolved in 4:1 toluene/methanol. The water adduct [**1** + Ni + H_2O]$^{2+}$ was found as the main peak, with traces of [**1** + Ni]$^{2+}$ and [**1** + Ni + H_2O]$^{2+}$.

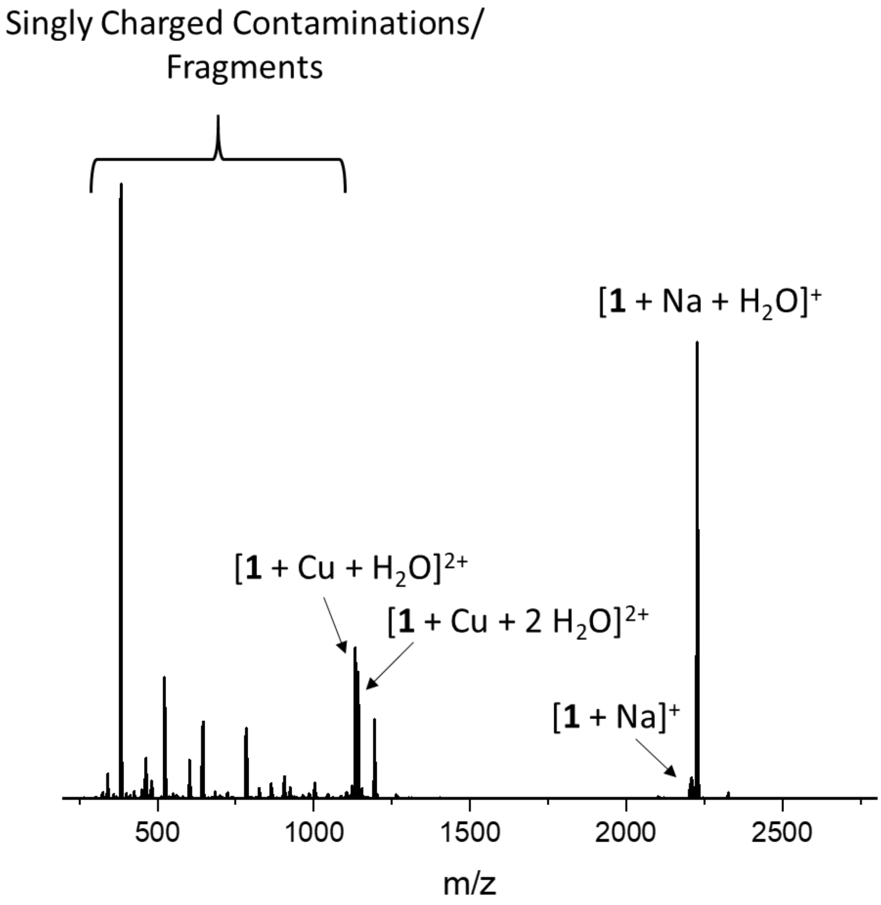

Figure S4: Mass spectrum of **1** and $Cu(CH_3COO)_2$ dissolved in 4:1 toluene/methanol. From the copper-containing ions, the water adduct [**1** + Cu + $H_2O]^{2+}$ was found as the main peak, with traces of [**1** + Cu + $H_2O]^{2+}$.

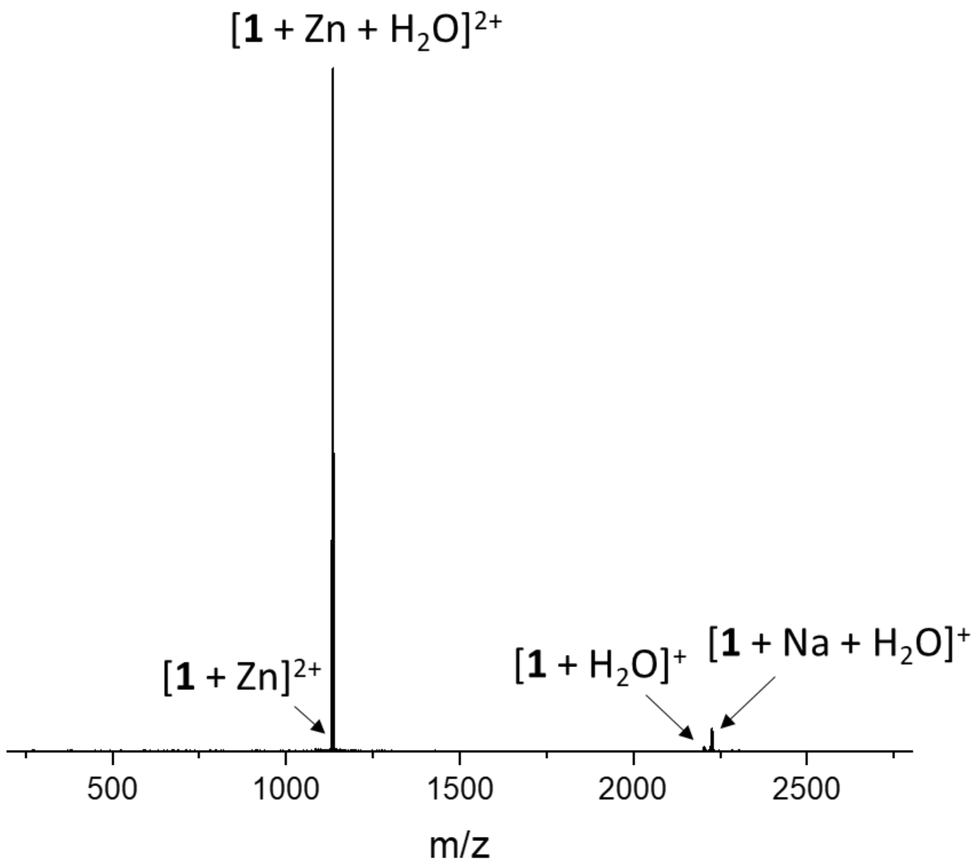

Figure S5: Mass spectrum of **1** and $ZnCl_2$ dissolved in 4:1 toluene/methanol. The water adduct [**1** + Zn + H_2O]$^{2+}$ was found as the main peak, with traces of [**1** + Zn]$^{2+}$.

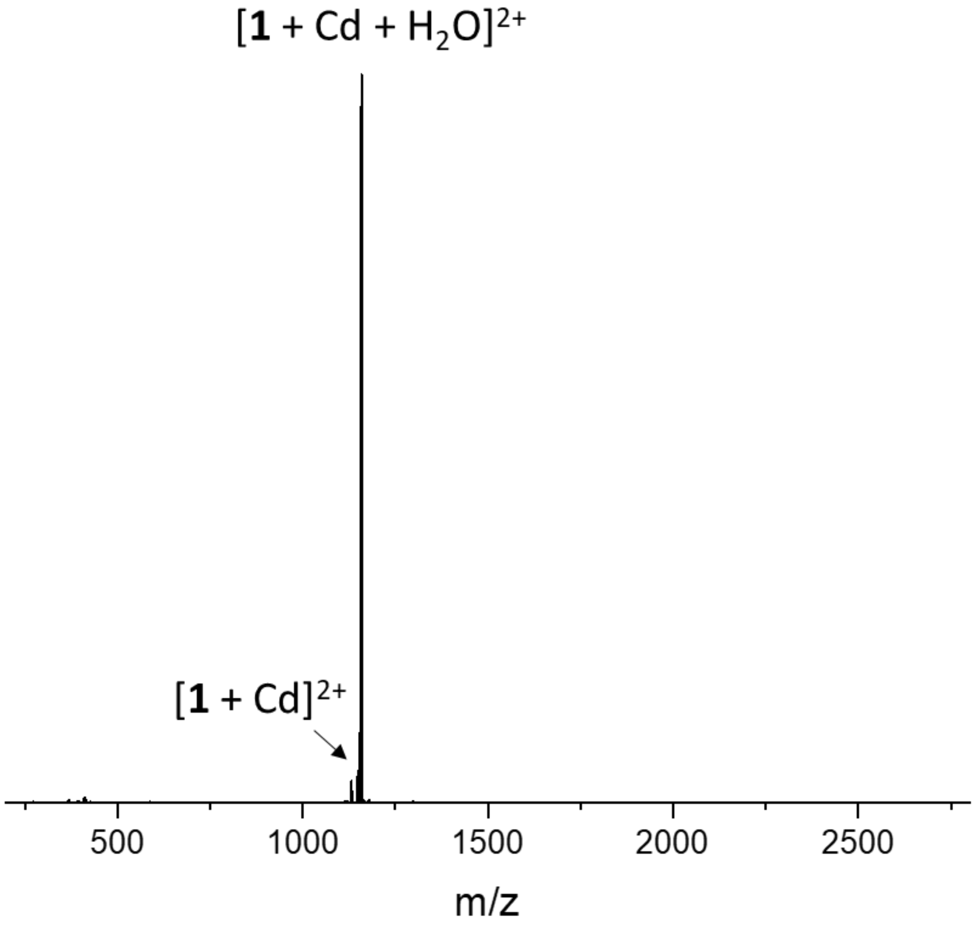

Figure S6: Mass spectrum of **1** and $Cd(NO_3)_2 \cdot 4\ H_2O$ dissolved in 4:1 toluene/methanol. The water adduct [**1** + Cd + H_2O]$^{2+}$ was found as the main peak, with traces of [**1** + Cd]$^{2+}$.

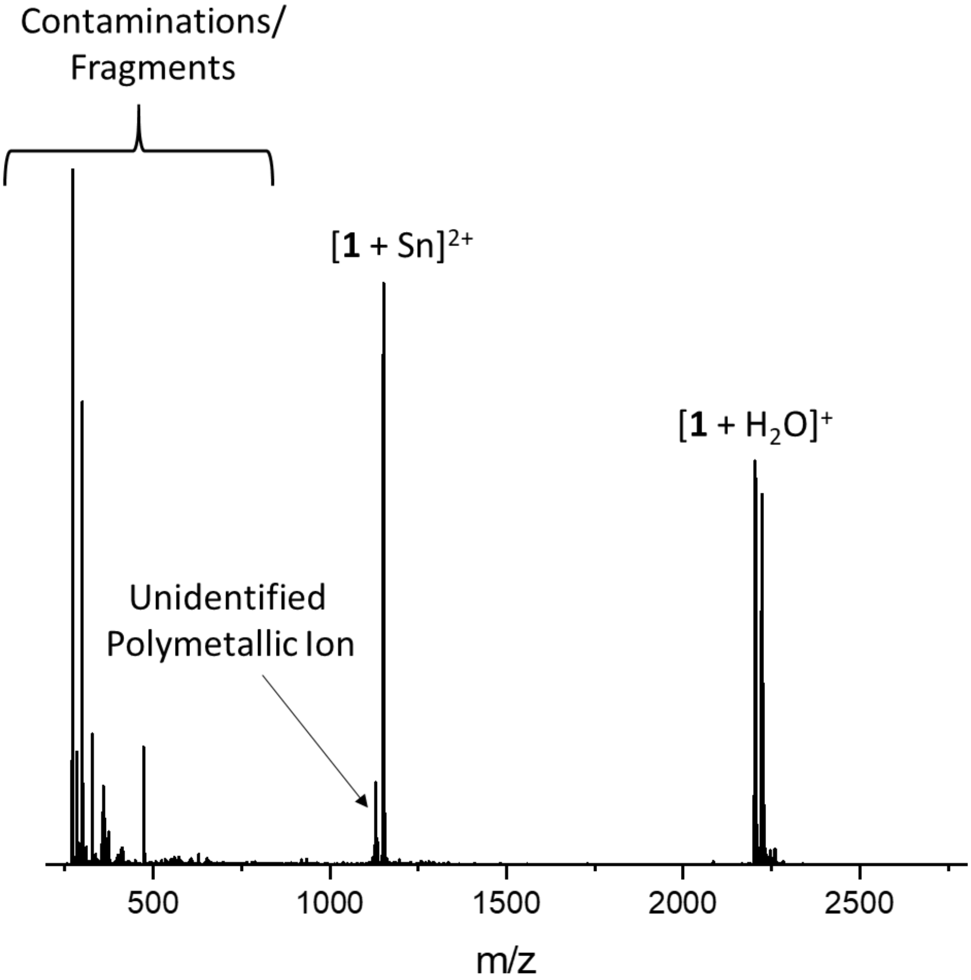

Figure S7: Mass spectrum of **1** and $SnCl_2 \cdot 2\ H_2O$ dissolved in 4:1 toluene/methanol. From the peaks containing tin, the ion [**1** + Sn + $H_2O]^{2+}$ was found as the main peak.

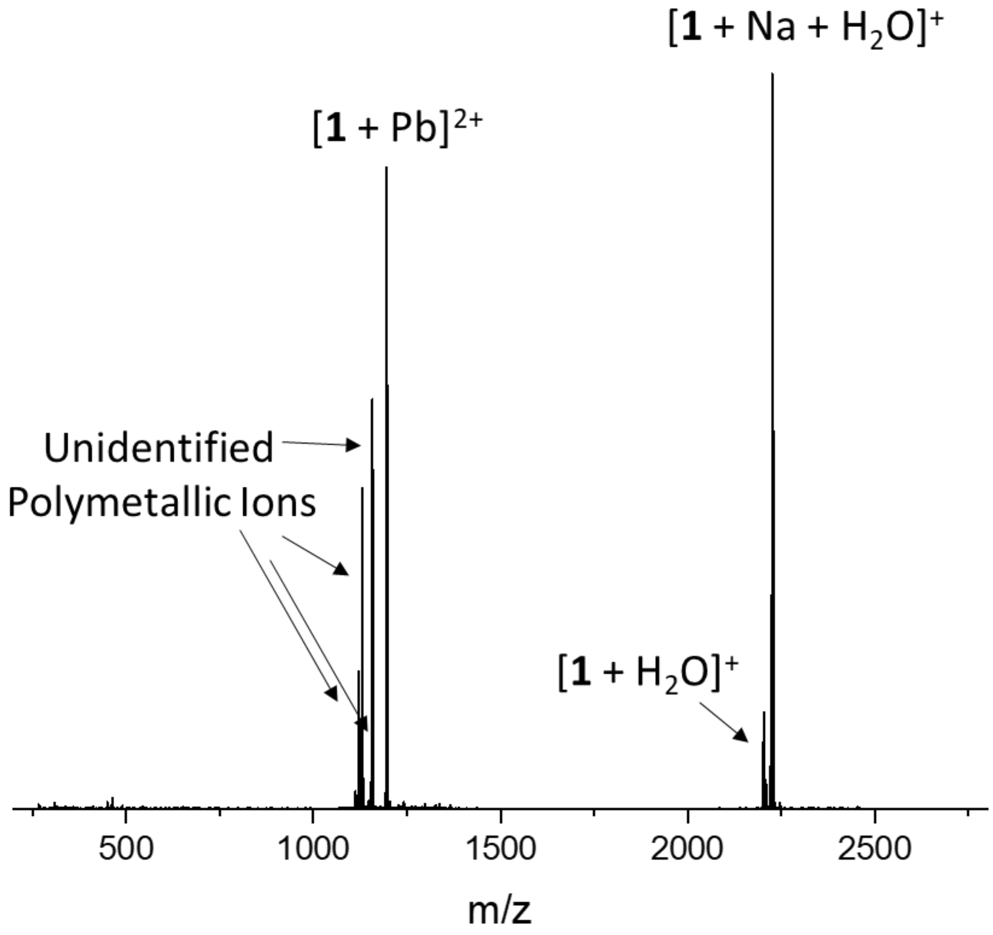

Figure S8: Mass spectrum of **1** and $Pb(NO_3)_2$ dissolved in 4:1 toluene/methanol. From the peaks containing lead, the ion [**1** + Pb]$^{2+}$ was found as the main peak.

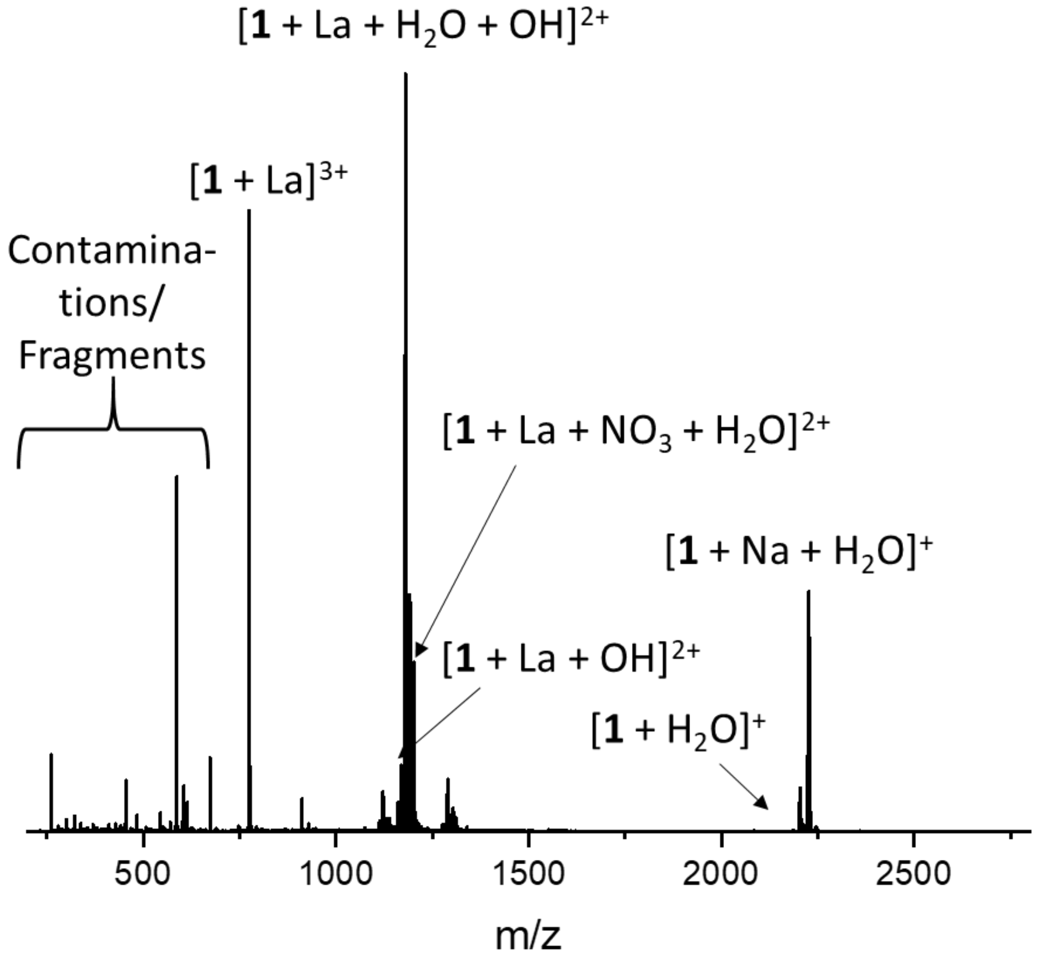

Figure S9: Mass spectrum of **1** and $La(NO_3)_3 \cdot 9\ H_2O$ dissolved in 4:1 toluene/methanol. Besides the triply charged [**1** + La]$^{3+}$, several doubly charged ions containing lanthanum were found.

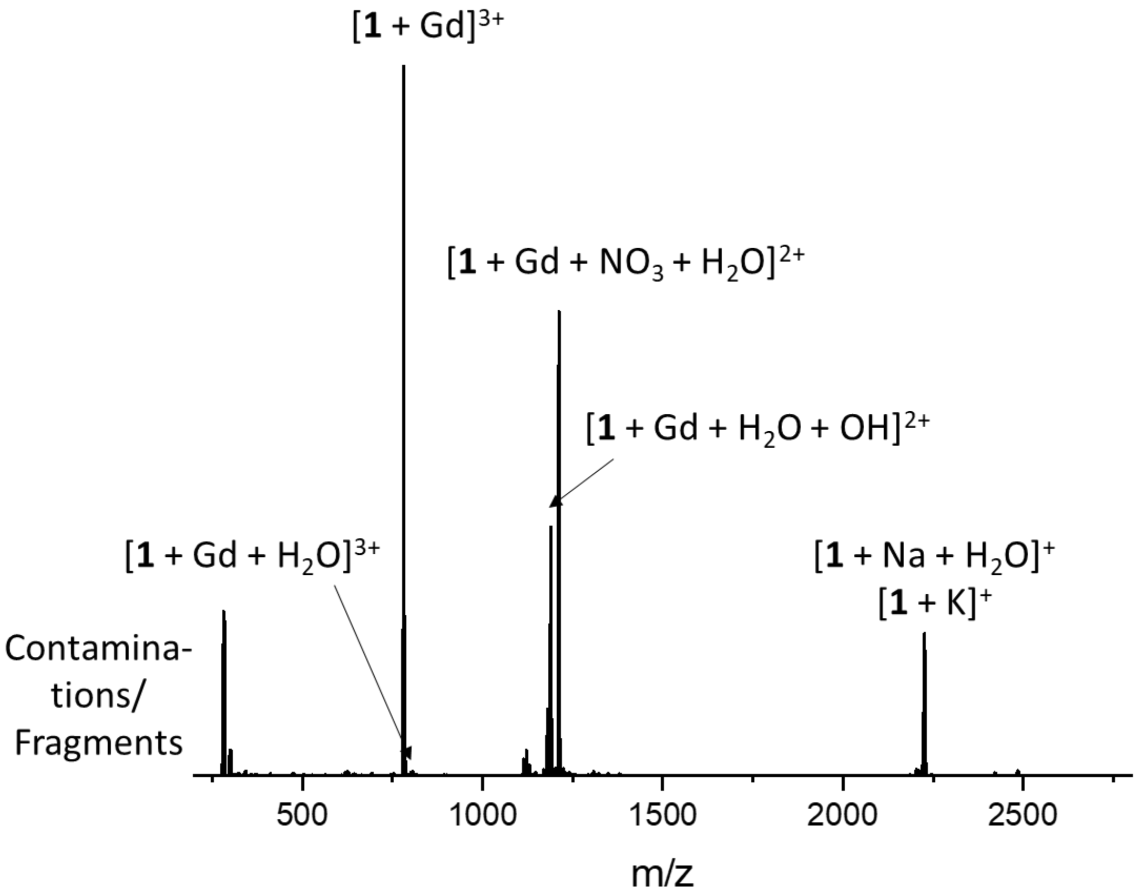

Figure S10: Mass spectrum of **1** and $Gd(NO_3)_3 \cdot 6\ H_2O$ dissolved in 4:1 toluene/methanol. Besides the triply charged [**1** + Gd]$^{3+}$ and its water adduct [**1** + Gd + H_2O]$^{3+}$, several doubly charged ions containing gadolinium were found.

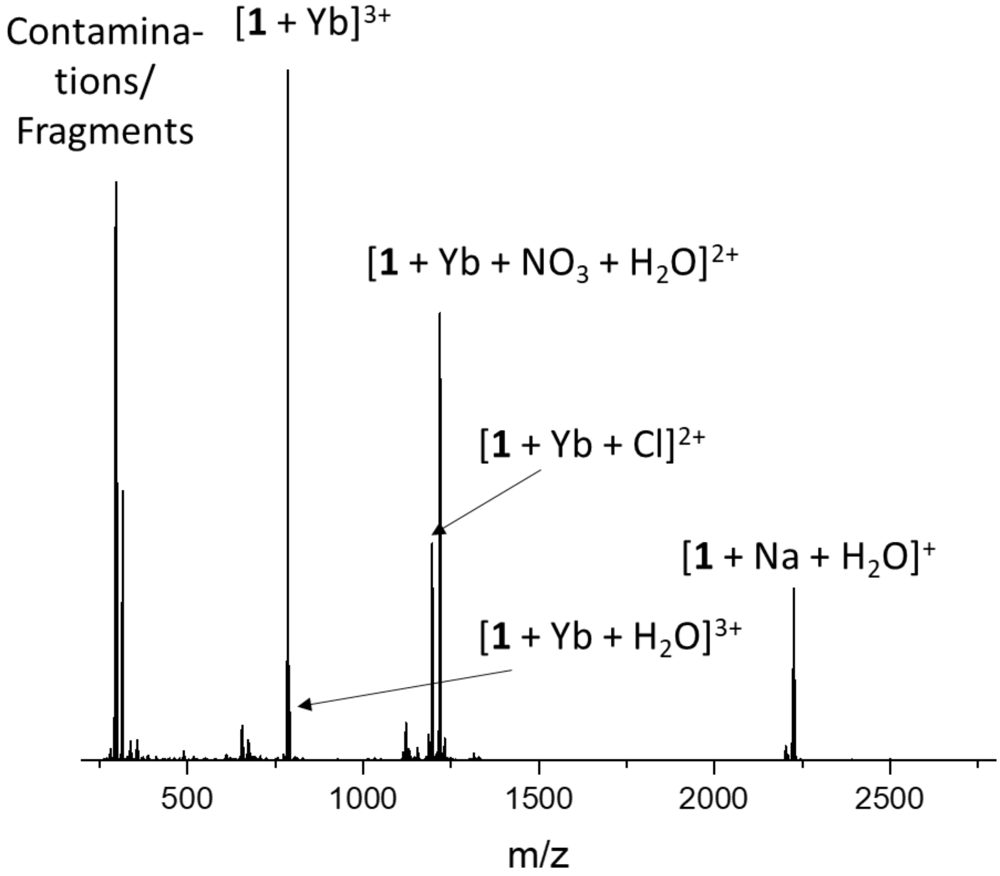

Figure S11: Mass spectrum of **1** and $YbCl_3 \cdot 5\ H_2O$ dissolved in 4:1 toluene/methanol. Besides the triply charged [**1** + Yb]$^{3+}$ and its water adduct [**1** + Yb + H_2O]$^{3+}$, several doubly charged ions containing gadolinium were found.

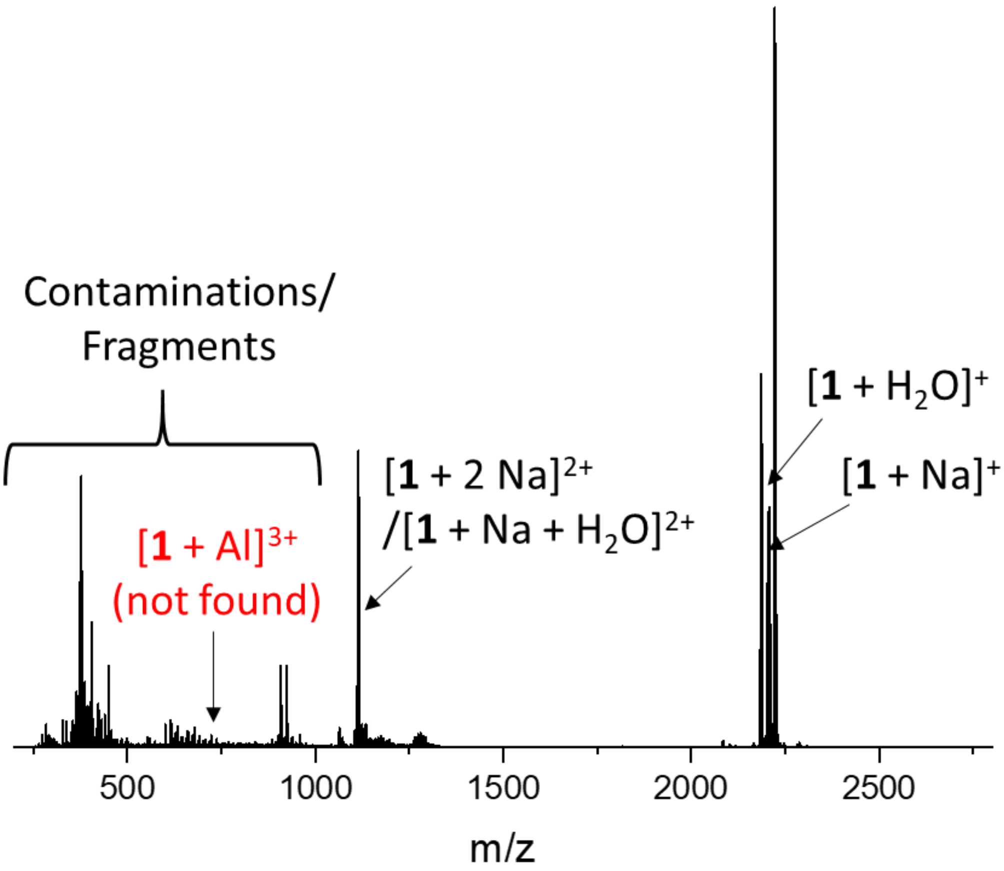

Figure S12: Mass spectrum of **1** and $Al(NO_3)_3 \cdot 9\ H_2O$ dissolved in 4:1 toluene/methanol. The spectrum appears with many peaks, however, no triply charged [**1** + Al]$^{3+}$ was found.

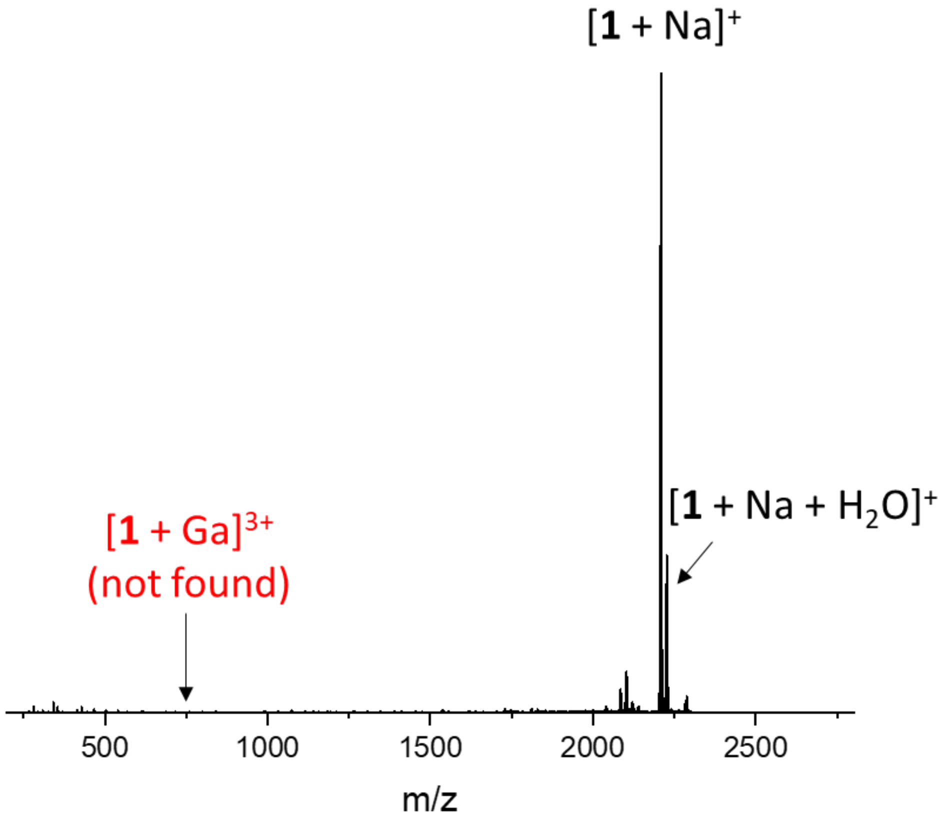

Figure S13: Mass spectrum of **1** and $Ga(NO_3)_3$ hydrate dissolved in 4:1 toluene/methanol. The spectrum appears almost exclusively with singly charged ions containing Na^+, no triply charged [**1** + Ga]$^{3+}$ was found.

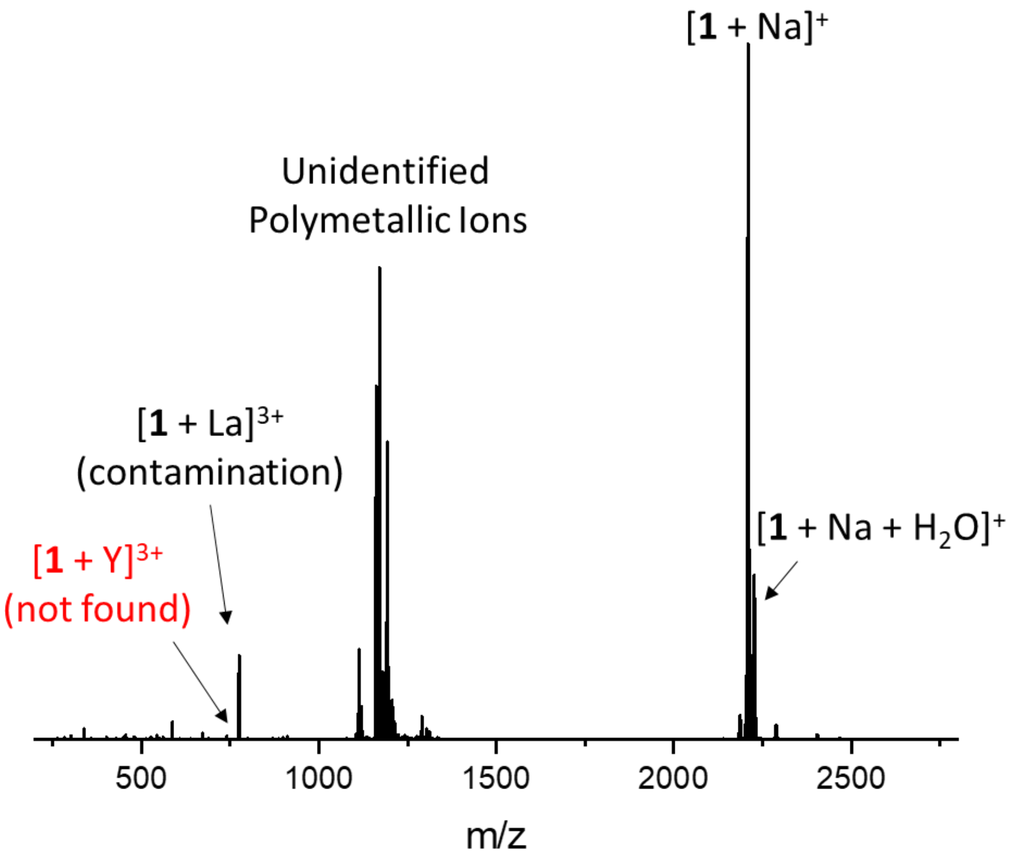

Figure S14: Mass spectrum of **1** and $Y(CH_3COO)_3$ hydrate dissolved in 4:1 toluene/methanol. The spectrum appears with many identified polymetallic ions, a [**1** + La]$^{3+}$ cross-contamination and singly-charged ions containing Na^+ and H_2O. No triply-charged [**1** + Y]$^{3+}$ was found.

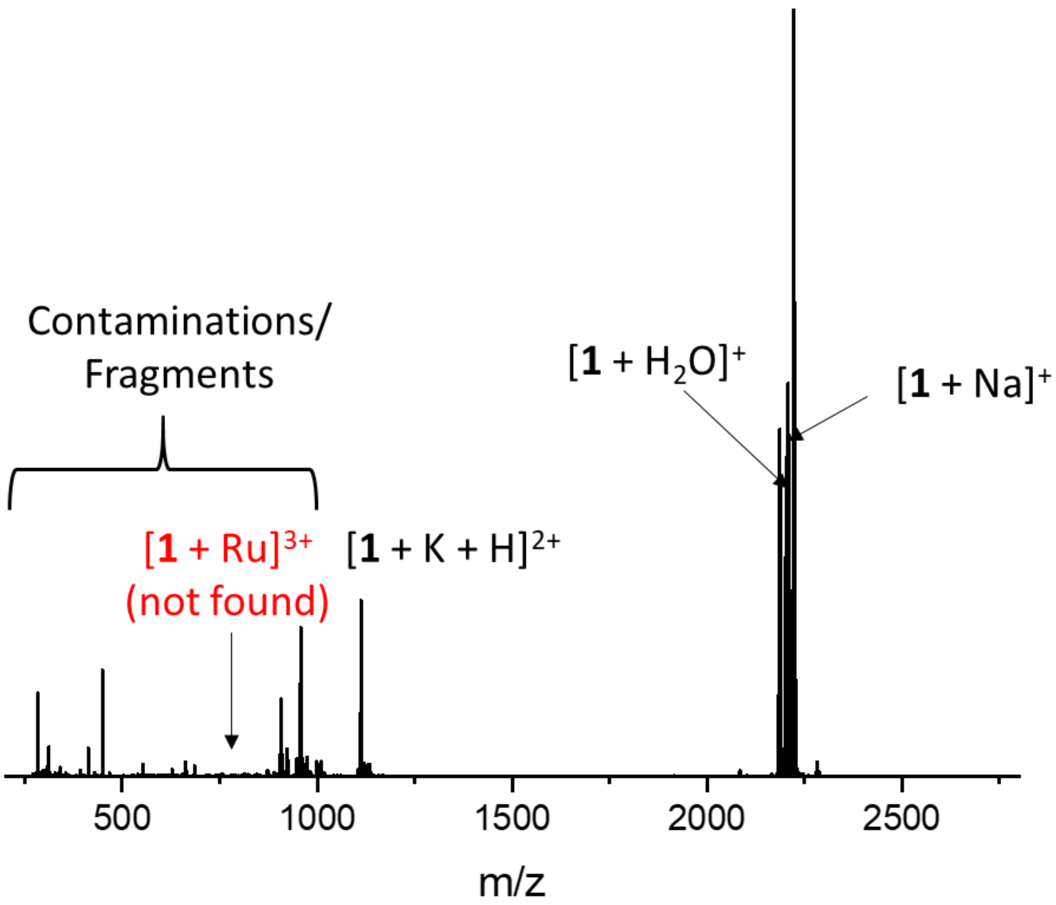

Figure S15: Mass spectrum of **1** and $RuCl_3$ hydrate dissolved in 4:1 toluene/methanol. The spectrum appears with many different ions, however no triply-charged [**1** + Ru]$^{3+}$ was found.

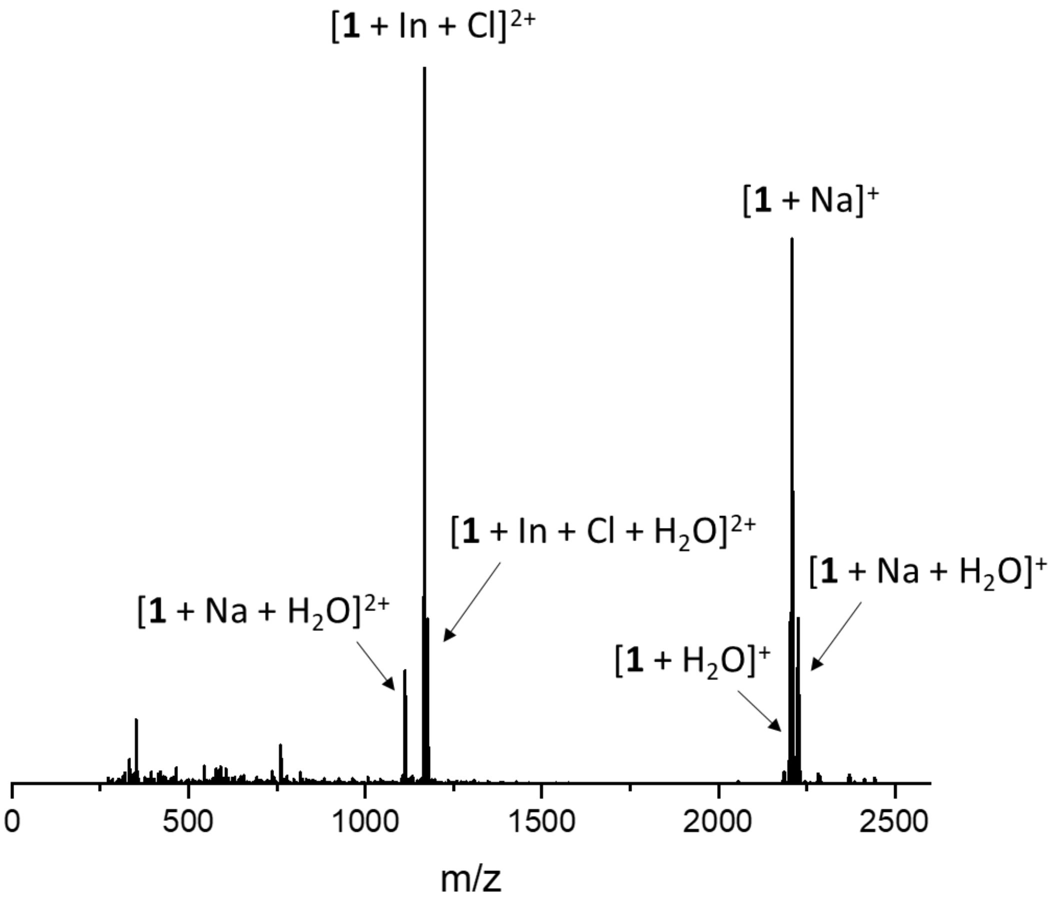

Figure S16: Mass spectrum of **1** and $InCl_3$ hydrate dissolved in 4:1 toluene/methanol. The chloride adduct of the indium-based host-guest complex was found as the base peak, along with the corresponding water adduct and peaks corresponding to adducts of **1** with Na^+ and H_2O.

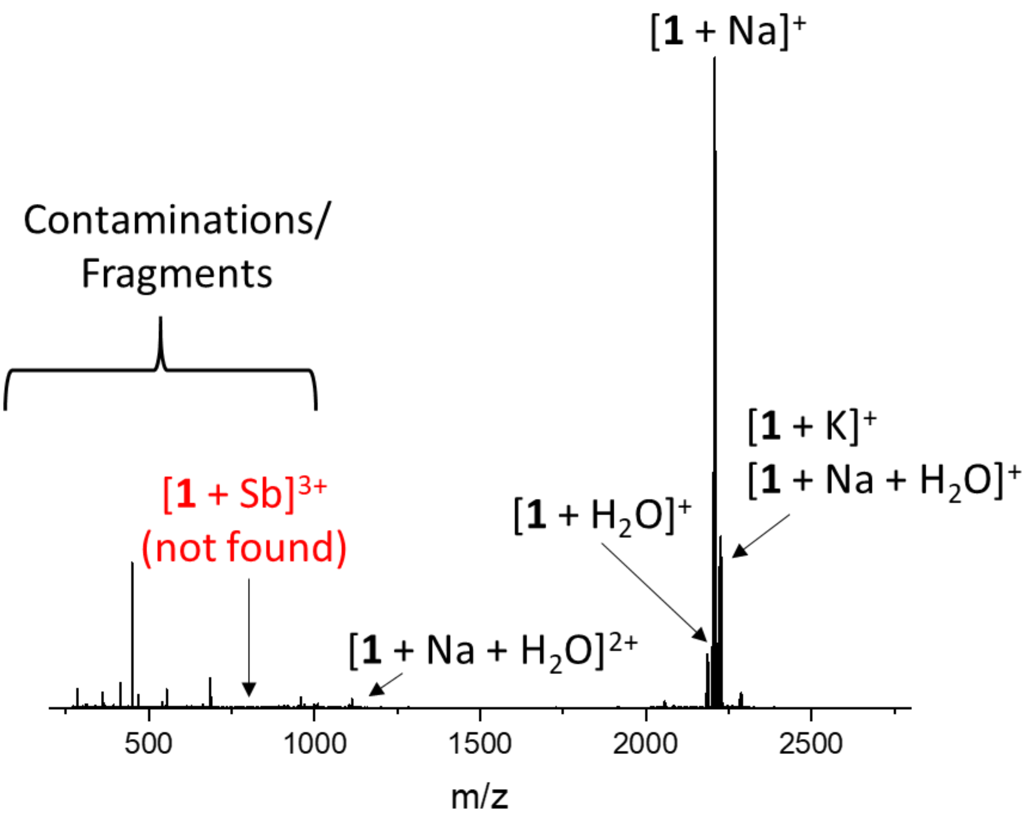

Figure S17: Mass spectrum of **1** and $Sb(CH_3COO)_3$ dissolved in 4:1 toluene/methanol. The spectrum appears almost exclusively with ions containing H_2O, Na^+ and K^+. No triply charged [**1** + Sb]$^{3+}$ was found.

Discussion on Water Binding in the Host-Guest Complexes

Under highly similar instrument parameters, we found that the singly charged Ag^+ forms almost exclusively the ion [**1** + Ag + H_2O]$^+$ and not [**1** + Ag]$^+$ (Figure S1), whereas the three studied lanthanides La^{3+}, Gd^{3+}, and Yb^{3+} yield [**1** + Ln]$^{3+}$ primarily, with only small amounts of [**1** + Ln + H_2O]$^{3+}$ present (Figures S9 – S11). The ratio of water adducts, relative to [**1** + Ln]$^{3+}$, increases in the order La^{3+}, Gd^{3+}, and Yb^{3+}. This trend follows the lanthanide contraction, suggesting that smaller cations tend to bind water in the cavity of the ring more strongly than the larger ones.[1] This agrees with our previous finding that Na^+ tends to be encapsulated along with H_2O in **1**, in contrast to the larger alkali metals K^+ and Cs^+.[2] For the doubly charged M^{2+}, we found the ion [**1** + M]$^{2+}$ to be dominant for M = Sn^{2+}, Pb^{2+} (Figures S7, S8), whereas Fe^{2+}, Co^{2+}, Ni^{2+}, Cu^{2+}, Zn^{2+}, and Cd^{2+} predominantly form the water adduct [**1** + M + H_2O]$^{2+}$ (Figures 3a, S2 – S6). This also follows the trend in ionic radius, which is larger for Sn^{2+} and Pb^{2+} than for the d-block cations.[3] For Ni^{2+} and more pronounced for Cu^{2+}, the double-water adduct [**1** + M + 2 H_2O]$^{2+}$ was found in significant amounts (Figures S3, S4). Intriguingly, this matches the Irving-Williams series where the thermodynamic binding of ligands is strongest to Ni^{2+} and Cu^{2+}.[4]

Table S1: $^{TW}CCS_{N2}$ and $^{TH}CCS_{N2}$ values of [**1** + M]$^{x+}$ and [**1** + M + H_2O]$^{x+}$. Values for [**1** + Ag]$^{+}$ and [**1** + Cu]$^{2+}$ were not obtained, as the former does only occur after collisional dissociation of [**1** + Ag + H_2O]$^{+}$ and the latter is not stable after *m/z*-selection without additional collisional activation. Theoretical $^{TH}CCS_{N2}$ values were simulated with IMoS and are 4-10% higher than experimental $^{TW}CCS_{N2}$ values. Calculating lanthanides with standard DFT methods is challenging, and hence $^{TH}CCS_{N2}$ calculations for [**1** + Ln]$^{3+}$ and [**1** + Ln + H_2O]$^{3+}$ (Ln = Gd^{3+}, Yb^{3+}) could not be realised.

**Metal Cation M^{x+}**	**$^{TW}CCS_{N2}$ (Å^2) of [1 + M]$^{x+}$**	**$^{TH}CCS_{N2}$ (Å^2) of [1 + M]$^{x+}$**	**$^{TW}CCS_{N2}$ (Å^2) of [1 + M + H_2O]$^{x+}$**	**$^{TH}CCS_{N2}$ (Å^2) of [1 + M + H_2O]$^{x+}$**
Ag^{+}	-	-	433.0 ± 1.3	468.1 ± 1.6
Fe^{2+}	443.1 ± 2.4	483.2 ± 1.1	446.5 ± 4.0	482.3 ± 1.9
Co^{2+}	442.1 ± 3.5	484.0 ± 2.4	450.1 ± 4.1	482.1 ± 2.4
Ni^{2+}	441.7 ± 3.3	483.8 ± 2.6	446.7 ± 3.8	480.3 ± 2.0
Cu^{2+}	-	-	449.9 ± 3.4	478.2 ± 1.7
Zn^{2+}	442.9 ± 3.3	483.1 ± 1.9	448.0 ± 3.9	478.4 ± 2.0
Cd^{2+}	442.3 ± 3.0	479.6 ± 2.0	447.0 ± 3.4	481.4 ± 1.4
Sn^{2+}	451.0 ± 3.9	482.2 ± 1.5	452.0 ± 3.5	482.1 ± 2.6
Pb^{2+}	449.3 ± 2.9	481.1 ± 1.4	451.5 ± 2.7	478.3 ± 1.6
La^{3+}	474.7 ± 2.6	492.2 ± 2.1	477.3 ± 2.6	504.5 ± 1.4
Gd^{3+}	473.5 ± 2.6	-	477.9 ± 2.6	-
Yb^{3+}	477.9 ± 2.6	-	478.5 ± 2.6	-

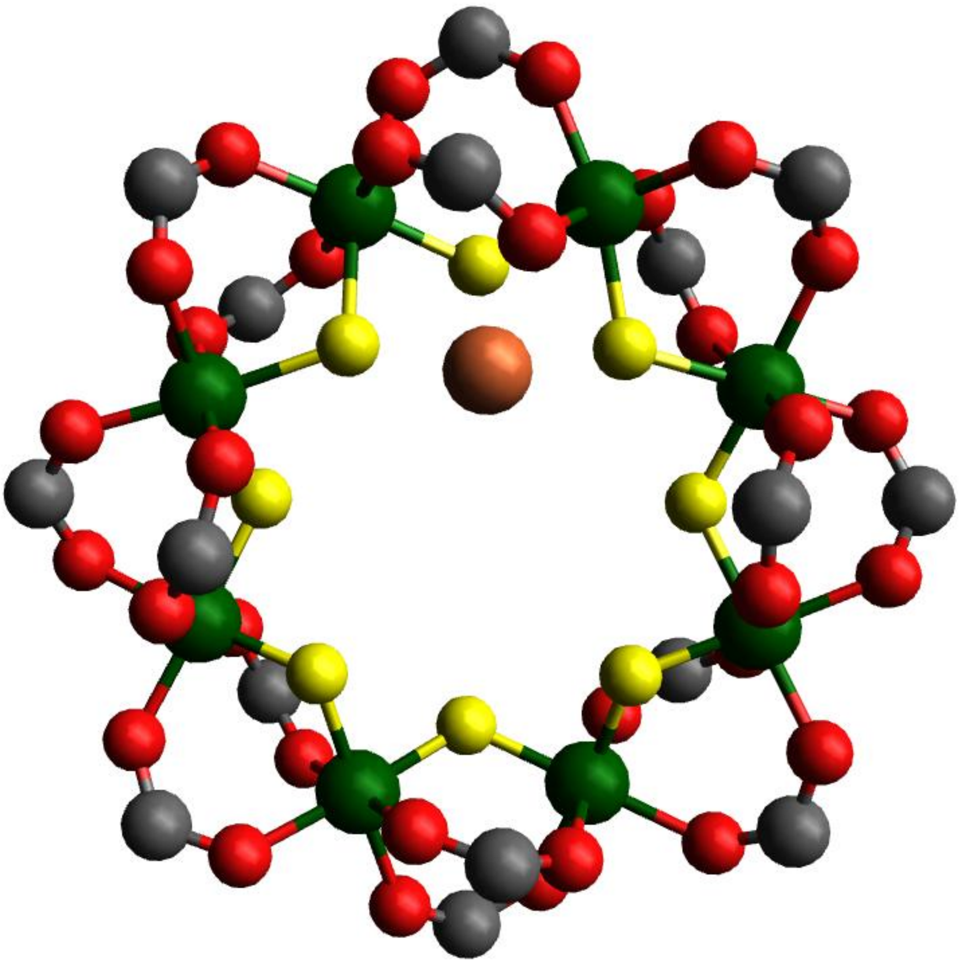

Figure S18: DFT optimised structure of [**1** + Fe]$^{2+}$ (Fe: orange, F: yellow, Cr: dark green, C: black, O: red). *Tert*-butyl groups were omitted for clarity. Optimised coordinates, ESP charges and the minimum energy can be found in the Supplementary Dataset.

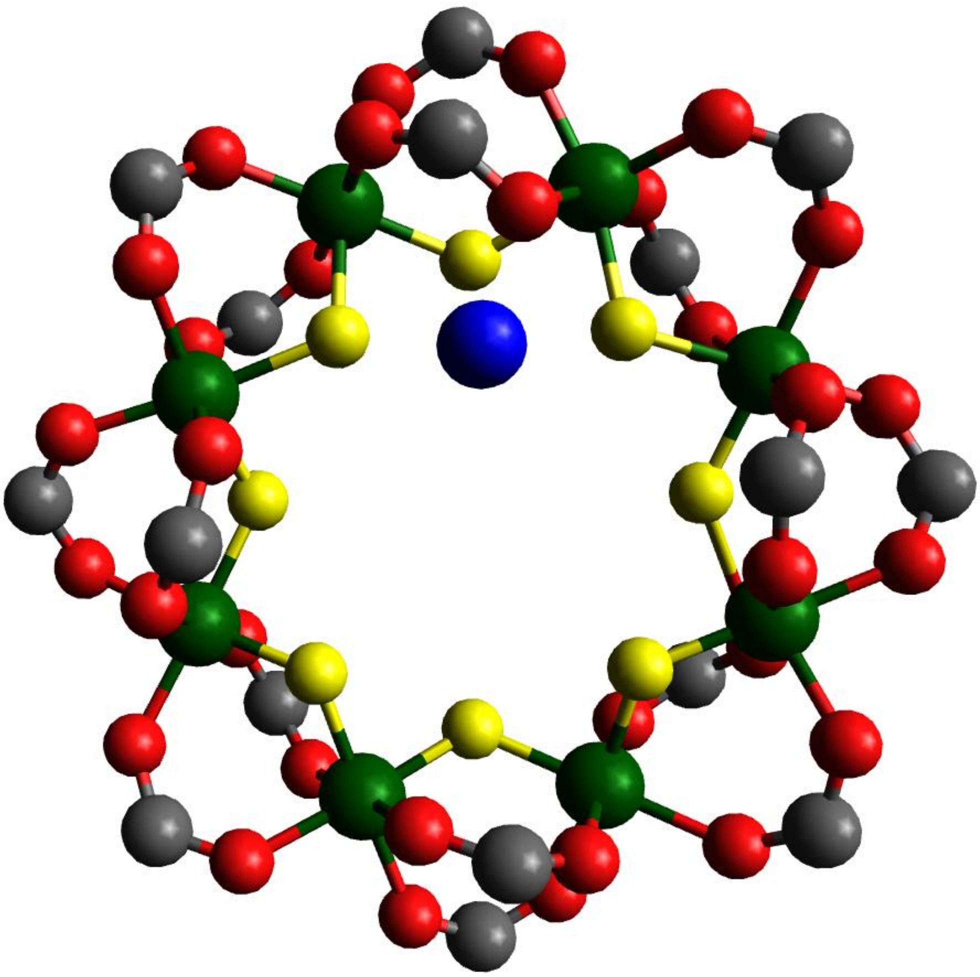

Figure S19: DFT optimised structure of [**1** + Co]$^{2+}$ (Co: dark blue, F: yellow, Cr: dark green, C: black, O: red). *Tert*-butyl groups were omitted for clarity. Optimised coordinates, ESP charges and the minimum energy can be found in the Supplementary Dataset.

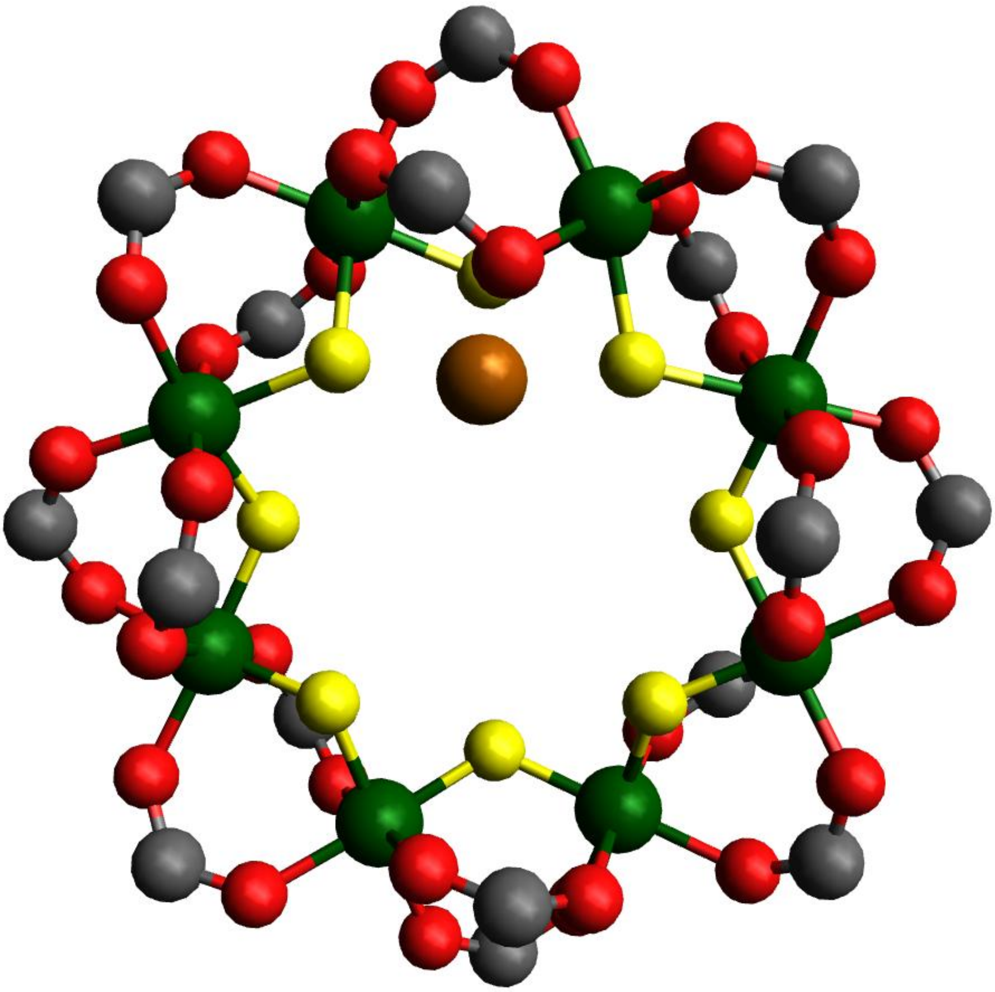

Figure S20: DFT optimised structure of [**1** + Ni]$^{2+}$ (Ni: brown, F: yellow, Cr: dark green, C: black, O: red). *Tert*-butyl groups were omitted for clarity. Optimised coordinates, ESP charges and the minimum energy can be found in the Supplementary Dataset.

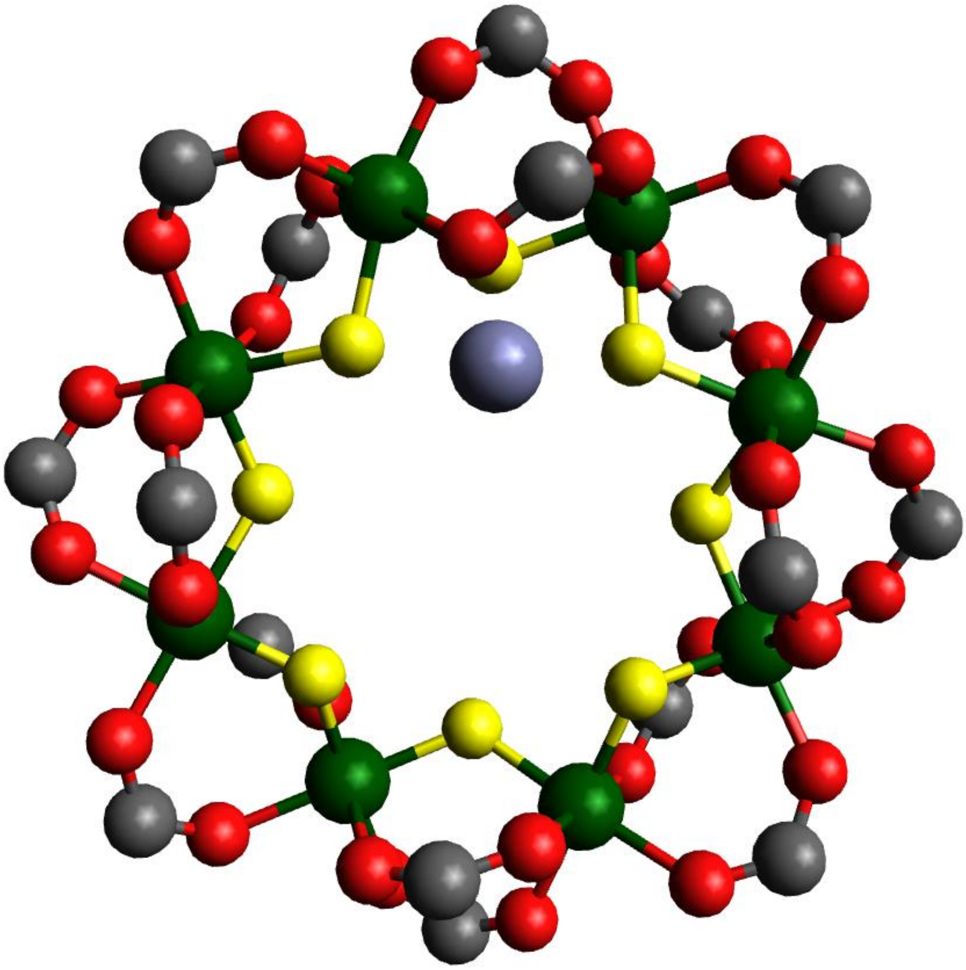

Figure S21: DFT optimised structure of [**1** + Zn]$^{2+}$ (Zn: blue grey, F: yellow, Cr: dark green, C: black, O: red). *Tert*-butyl groups were omitted for clarity. Optimised coordinates, ESP charges and the minimum energy can be found in the Supplementary Dataset.

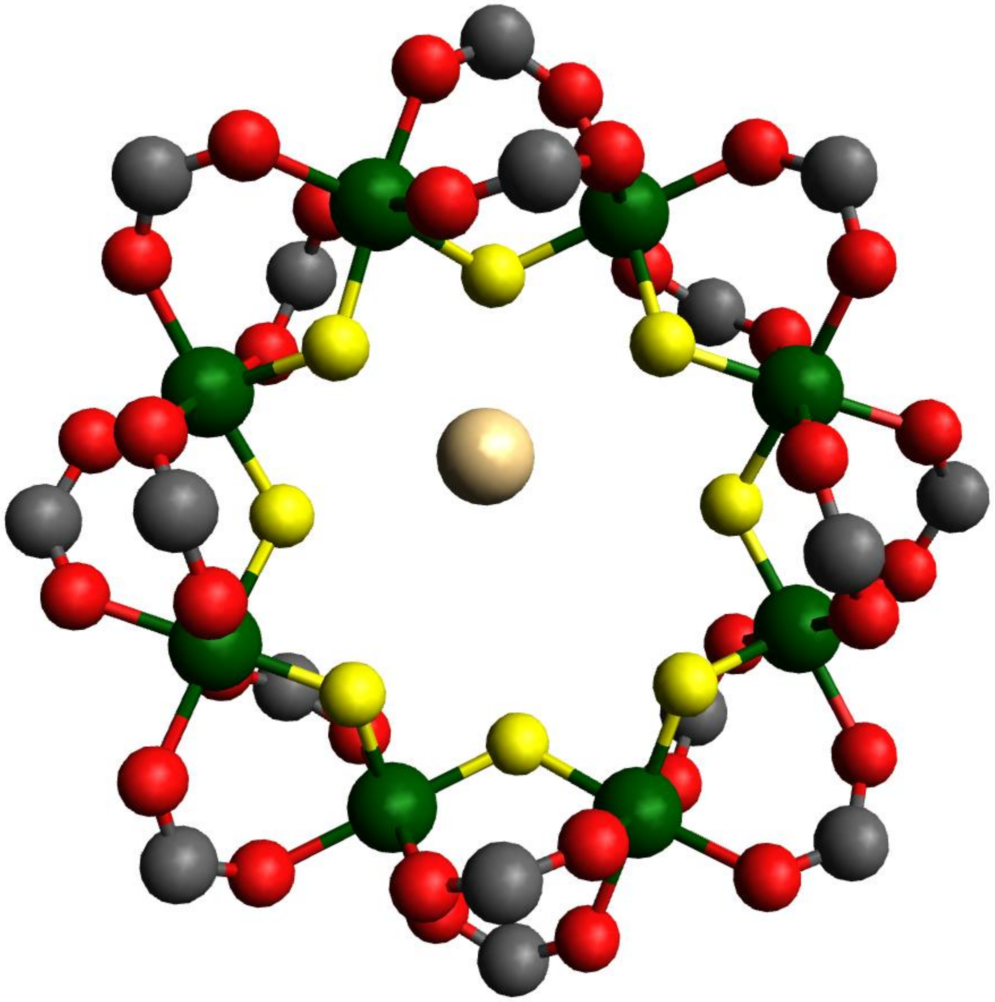

Figure S22: DFT optimised structure of [**1** + Cd]$^{2+}$ (Cd: gold, F: yellow, Cr: dark green, C: black, O: red). *Tert*-butyl groups were omitted for clarity. Optimised coordinates, ESP charges and the minimum energy can be found in the Supplementary Dataset.

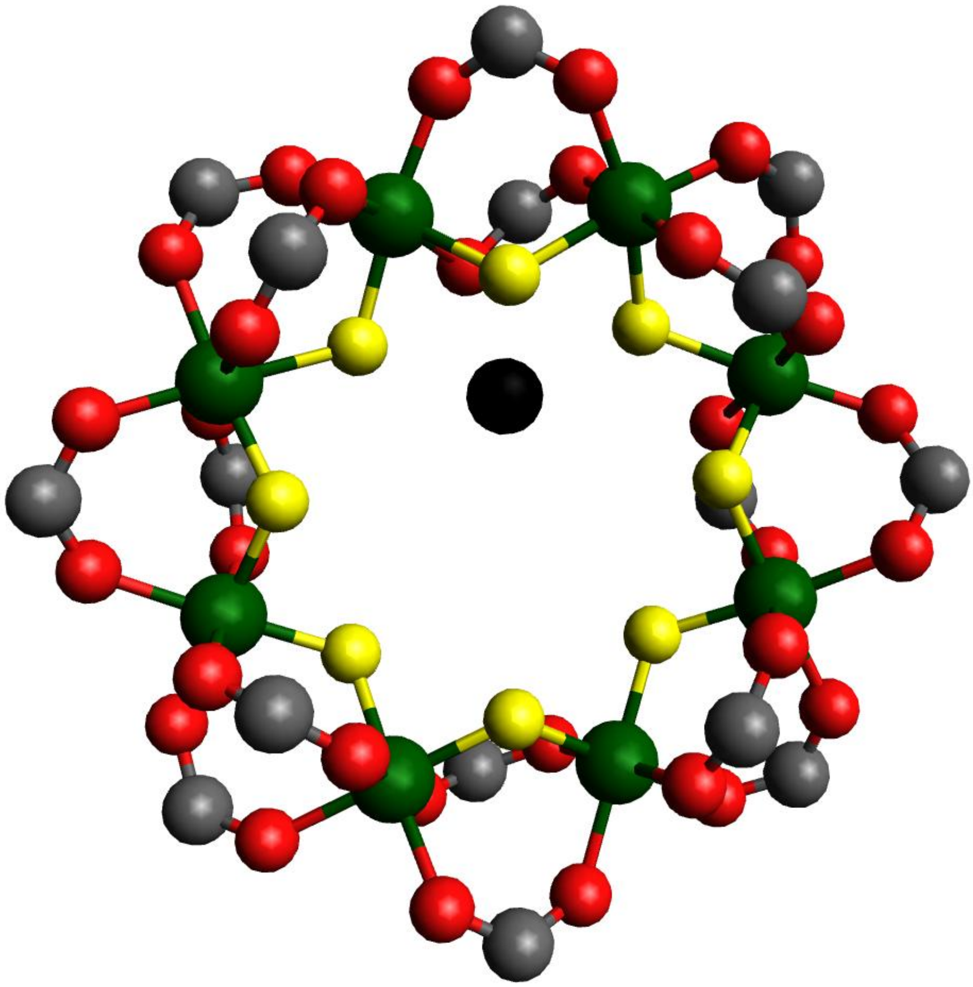

Figure S23: DFT optimised structure of [**1** + Sn]$^{2+}$ (Sn: dark black, F: yellow, Cr: dark green, C: black, O: red). *Tert*-butyl groups were omitted for clarity. Optimised coordinates, ESP charges and the minimum energy can be found in the Supplementary Dataset.

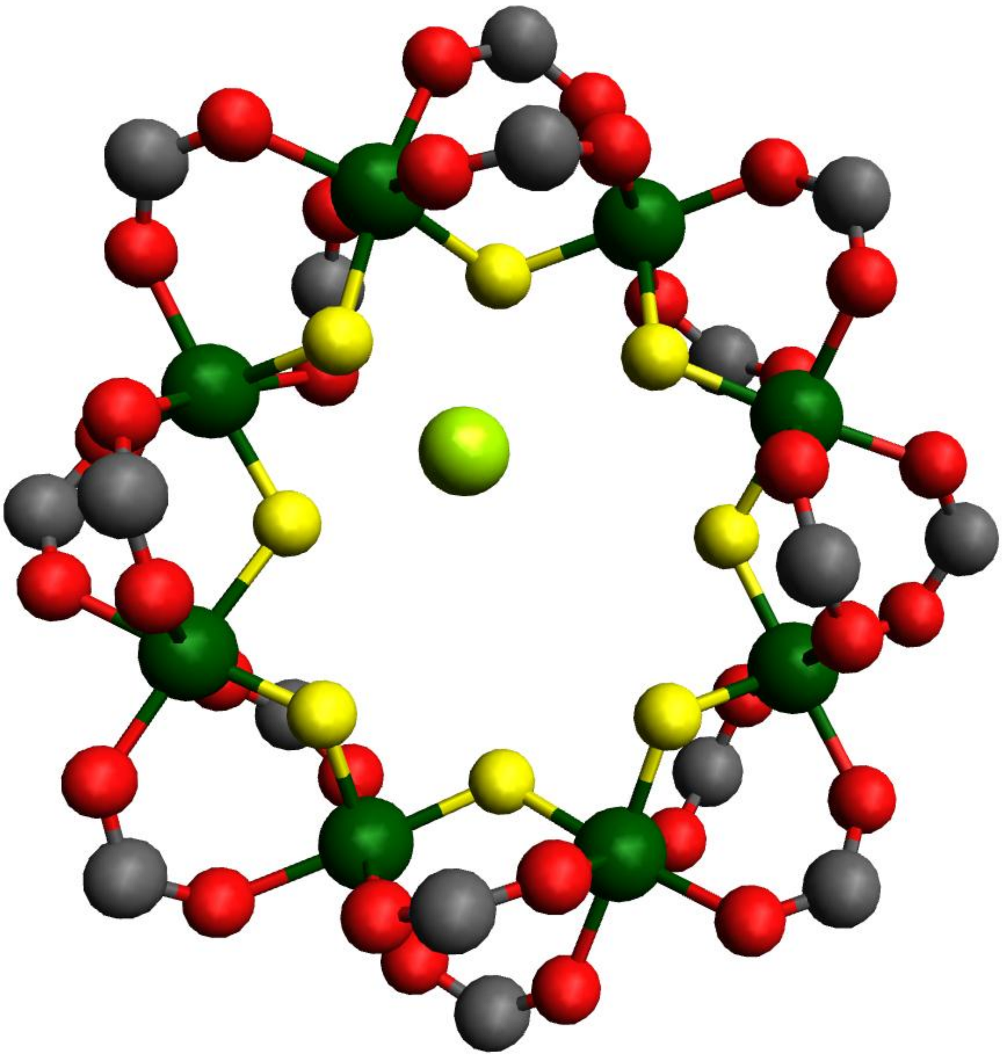

Figure S24: DFT optimised structure of [**1** + Pb]$^{2+}$ (Pb: light green, F: yellow, Cr: dark green, C: black, O: red). *Tert*-butyl groups were omitted for clarity. Optimised coordinates, ESP charges and the minimum energy can be found in the Supplementary Dataset.

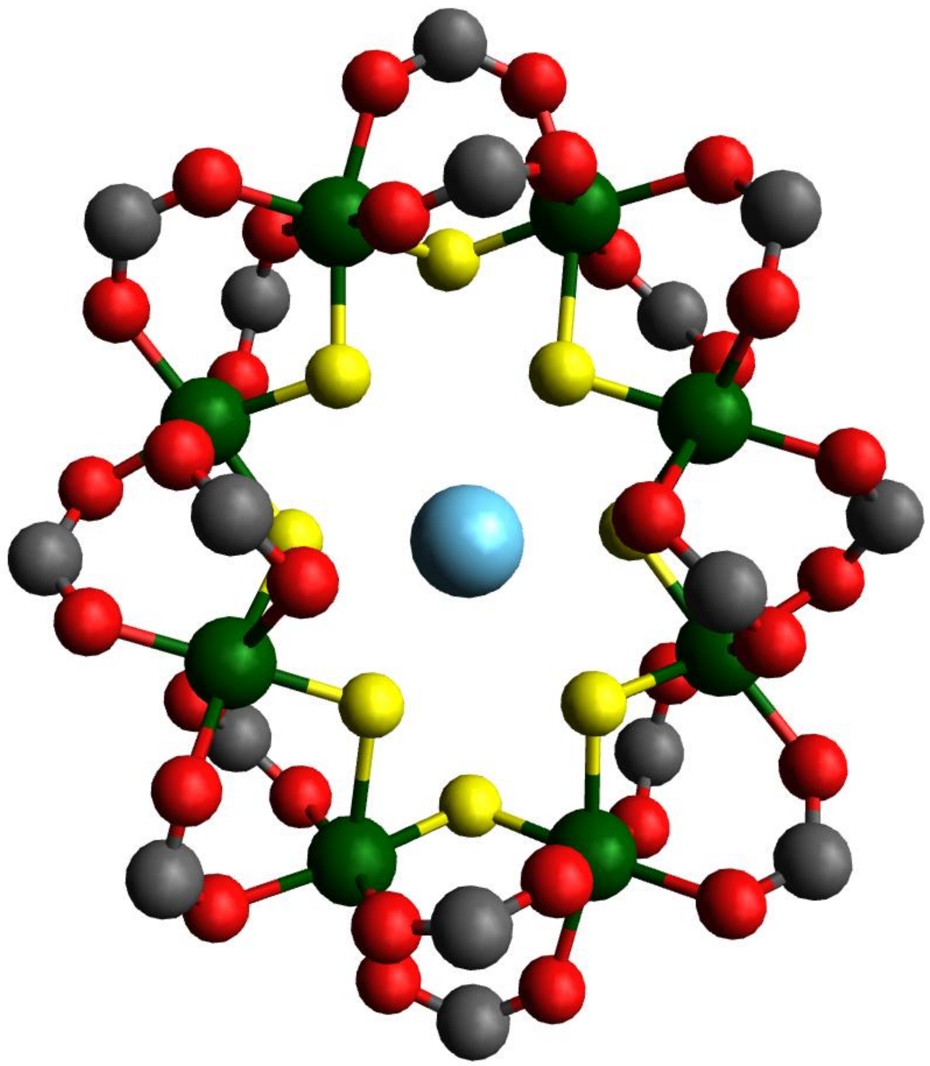

Figure S25: DFT optimised structure of [**1** + La]$^{3+}$ (La: cyan, F: yellow, Cr: dark greent, C: black, O: red). *Tert*-butyl groups were omitted for clarity. Optimised coordinates, ESP charges and the minimum energy can be found in the Supplementary Dataset.

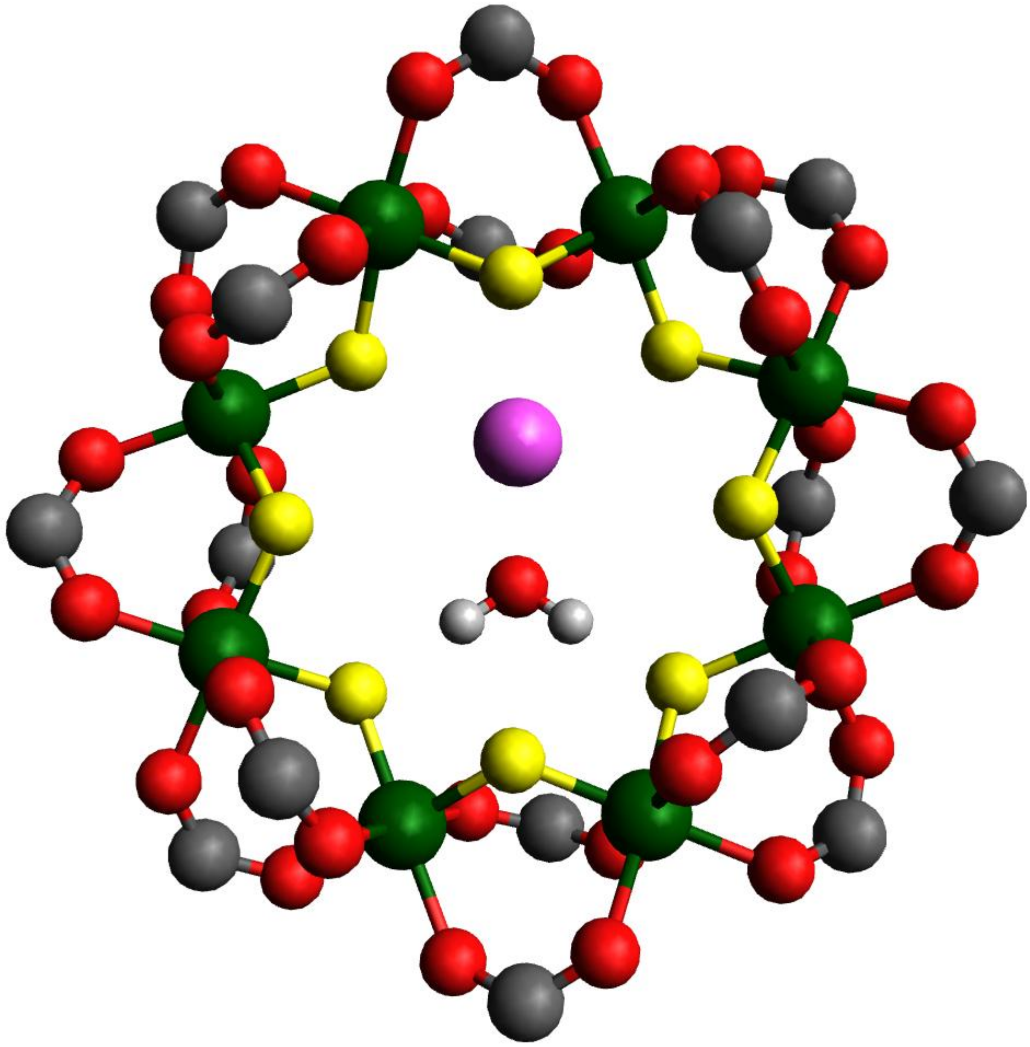

Figure S26: DFT optimised structure of [**1** + Ag + H_2O]$^+$ (Ag: pink, F: yellow, Cr: dark green, C: black, O: red, H: white). *Tert*-butyl groups were omitted for clarity. Optimised coordinates, ESP charges and the minimum energy can be found in the Supplementary Dataset.

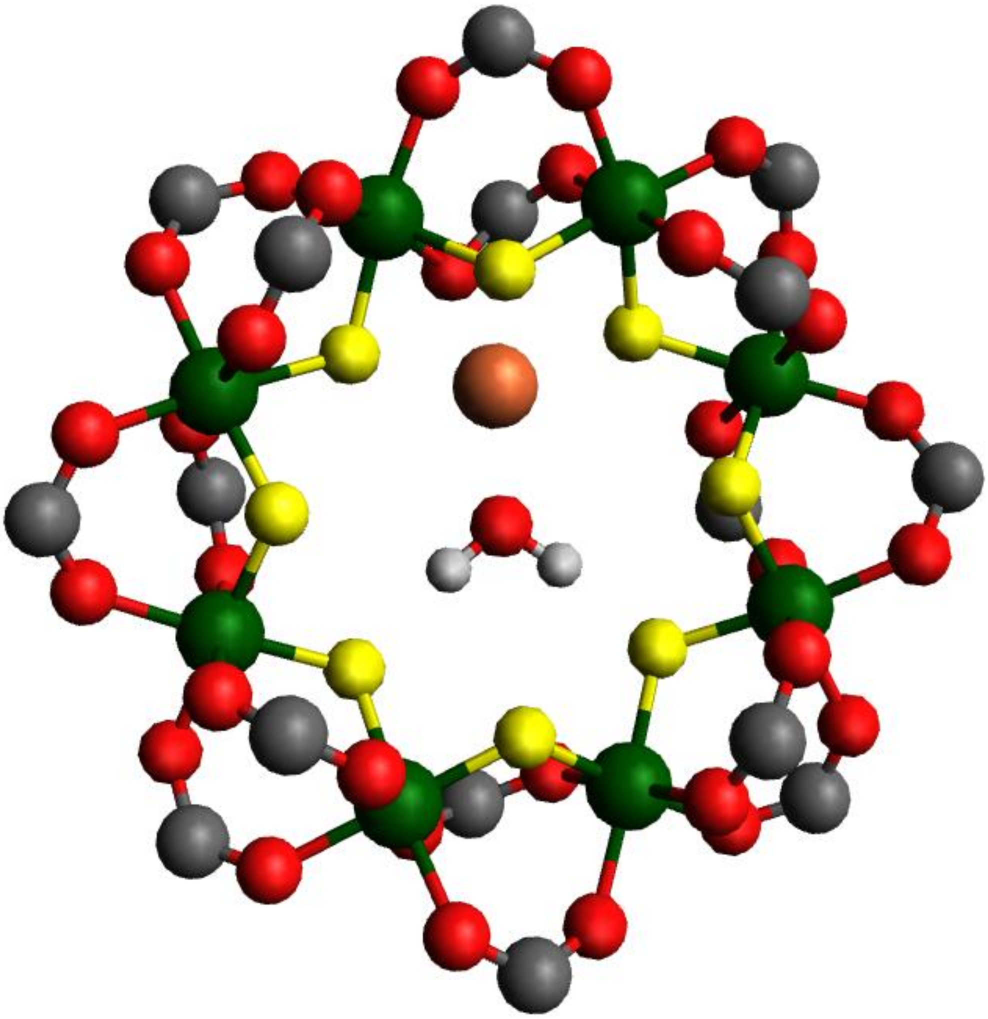

Figure S27: DFT optimised structure of [**1** + Fe + H_2O]$^{2+}$ (Fe: orange, F: yellow, Cr: dark green, C: black, O: red, H: white). *Tert*-butyl groups were omitted for clarity. Optimised coordinates, ESP charges and the minimum energy can be found in the Supplementary Dataset.

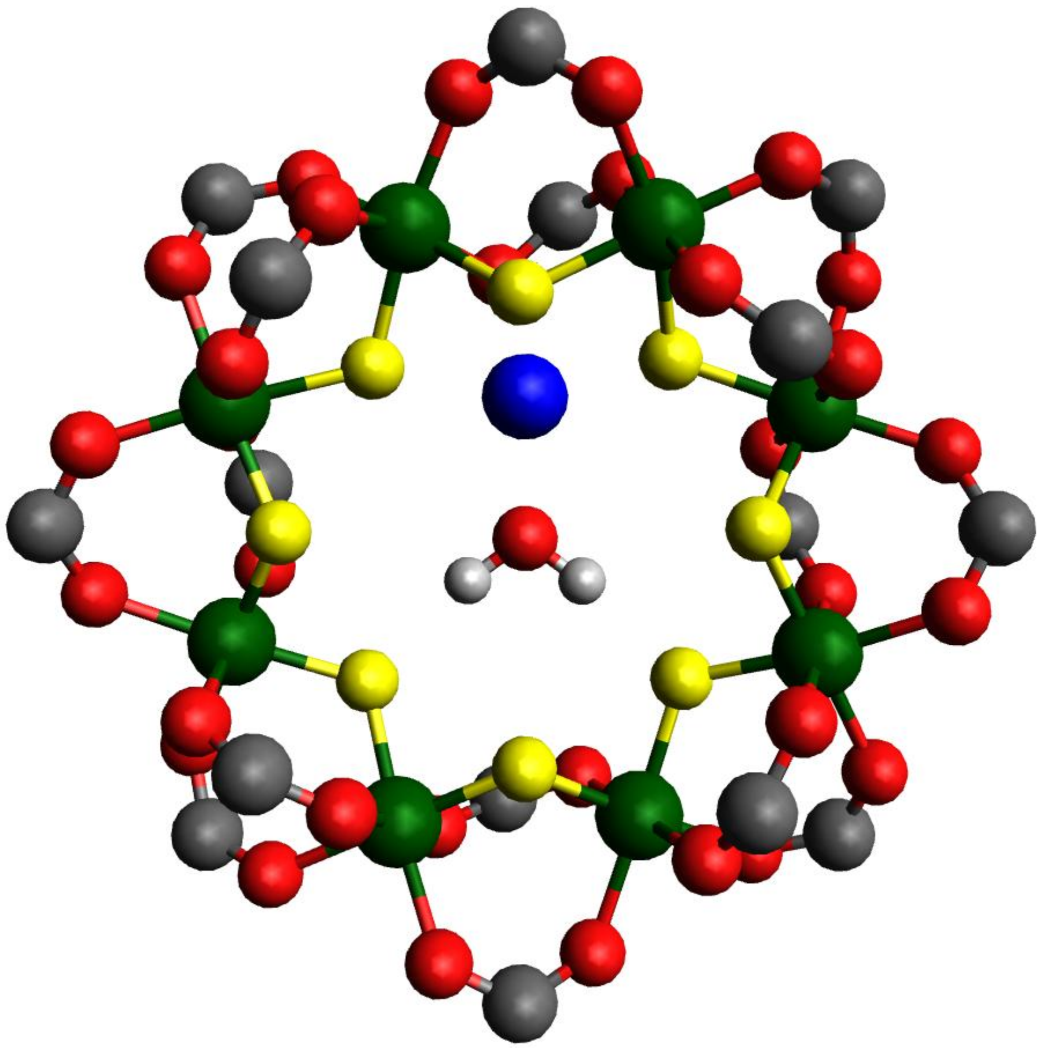

Figure S28: DFT optimised structure of [**1** + Co + H_2O]$^{2+}$ (Co: dark blue, F: yellow, Cr: dark green, C: black, O: red, H: white). *Tert*-butyl groups were omitted for clarity. Optimised coordinates, ESP charges and the minimum energy can be found in the Supplementary Dataset.

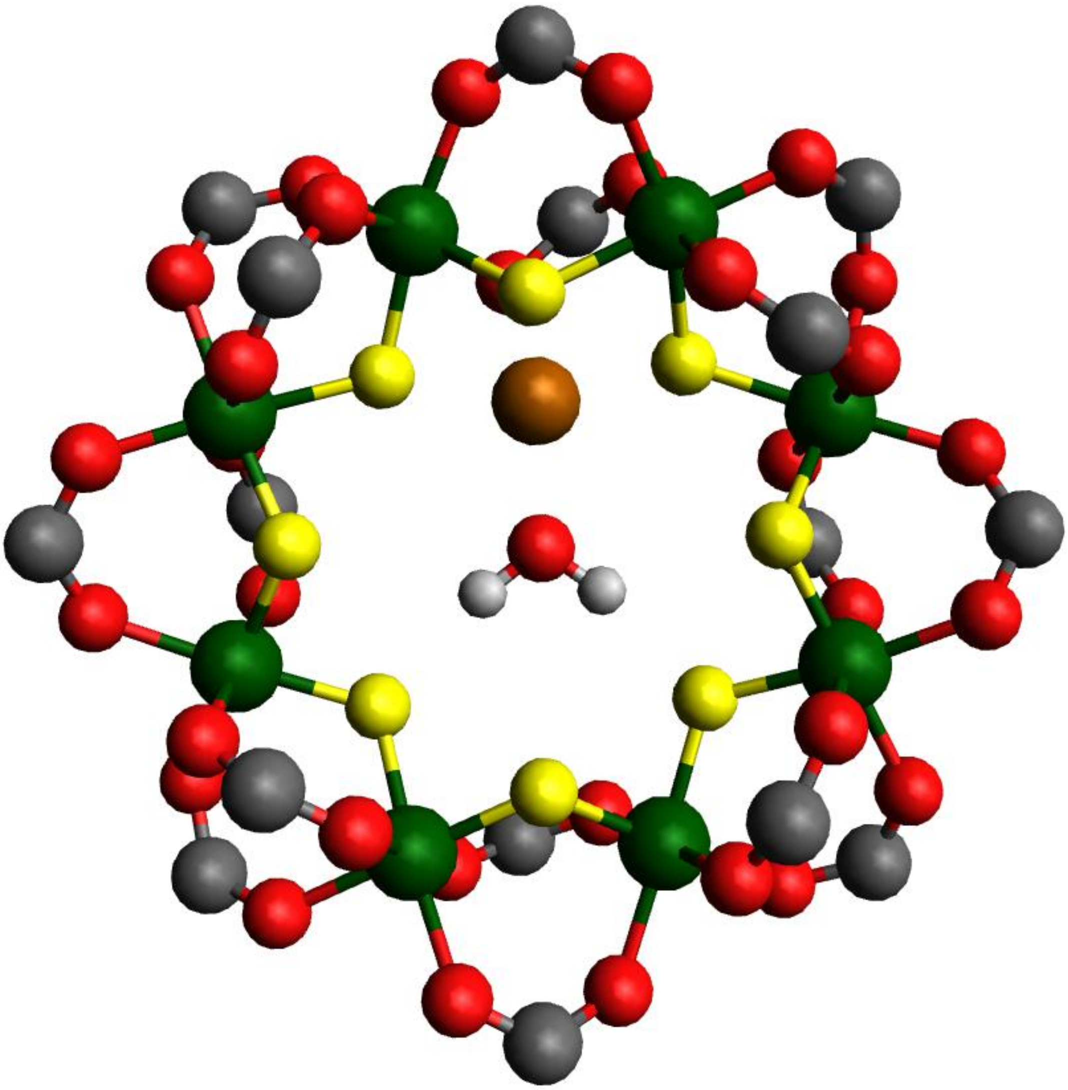

Figure S29: DFT optimised structure of [**1** + Ni + H_2O]$^{2+}$ (Ni: brown, F: yellow, Cr: dark green, C: black, O: red, H: white). *Tert*-butyl groups were omitted for clarity. Optimised coordinates, ESP charges and the minimum energy can be found in the Supplementary Dataset.

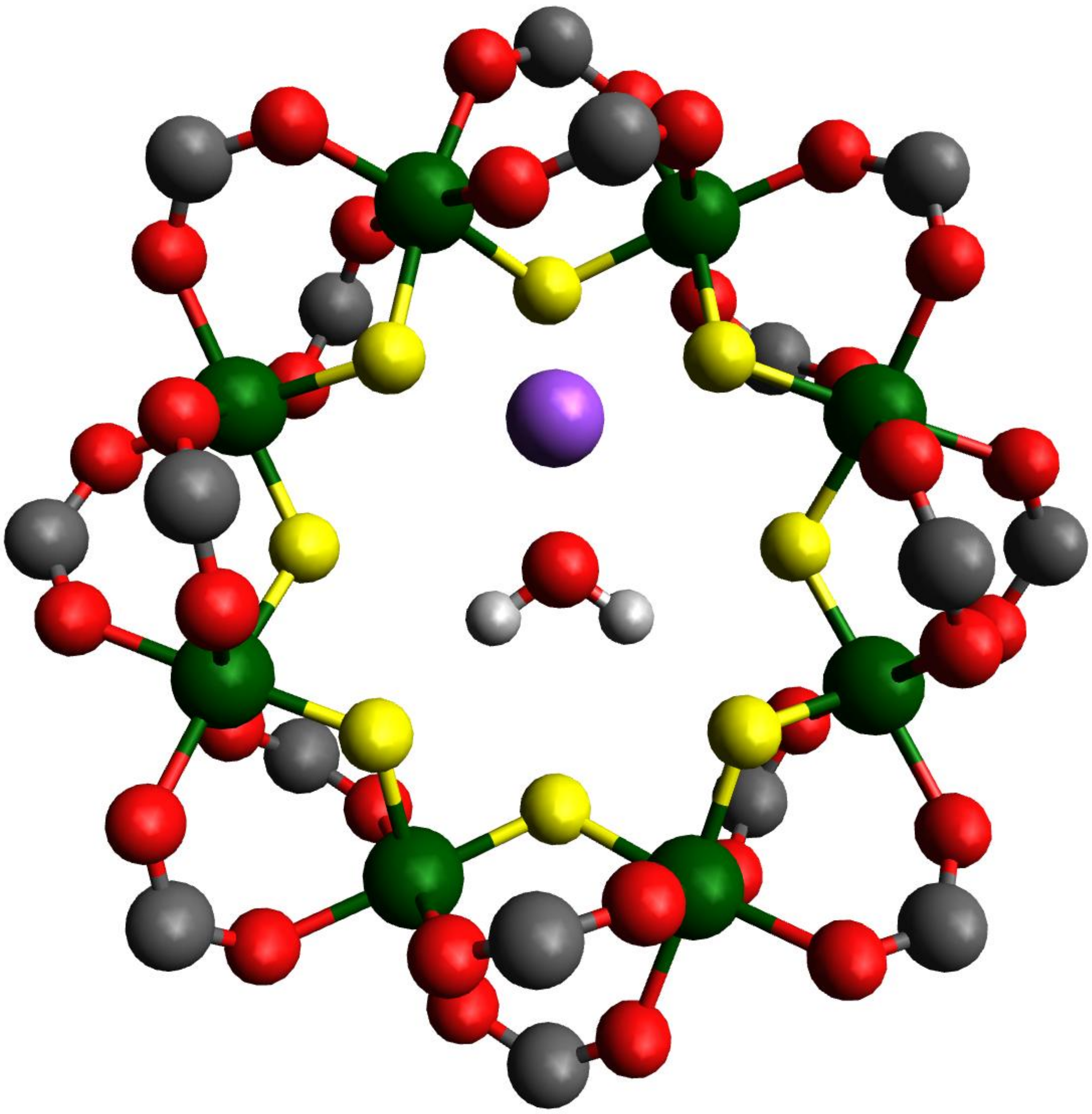

Figure S30: DFT optimised structure of [**1** + Cu + H_2O]$^{2+}$ (Cu: purple, F: yellow, Cr: dark green, C: black, O: red, H: white). *Tert*-butyl groups were omitted for clarity. Optimised coordinates, ESP charges and the minimum energy can be found in the Supplementary Dataset.

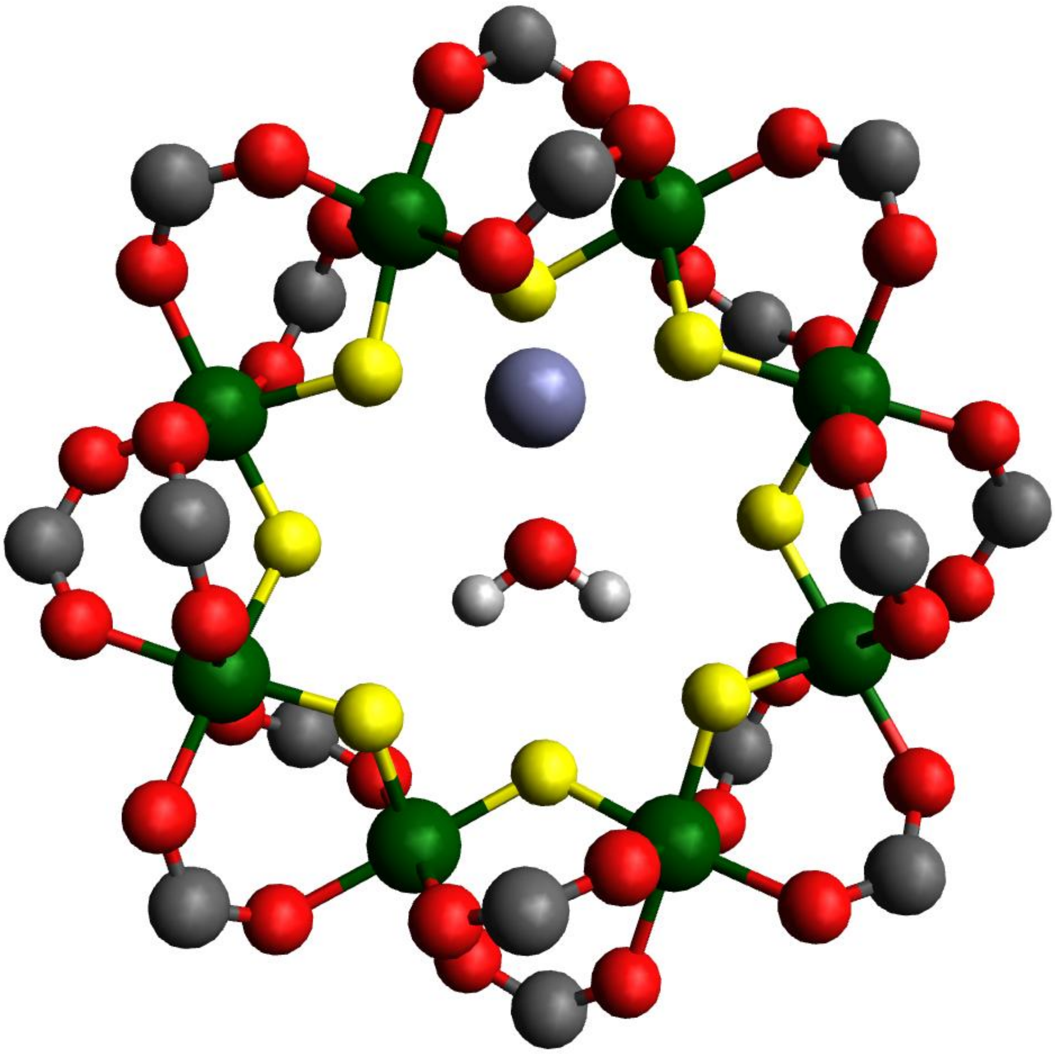

Figure S31: DFT optimised structure of [**1** + Zn + H_2O]$^{2+}$ (Zn: blue grey, F: yellow, Cr: dark blue, C: black, O: red, H: white). *Tert*-butyl groups were omitted for clarity. Optimised coordinates, ESP charges and the minimum energy can be found in the Supplementary Dataset.

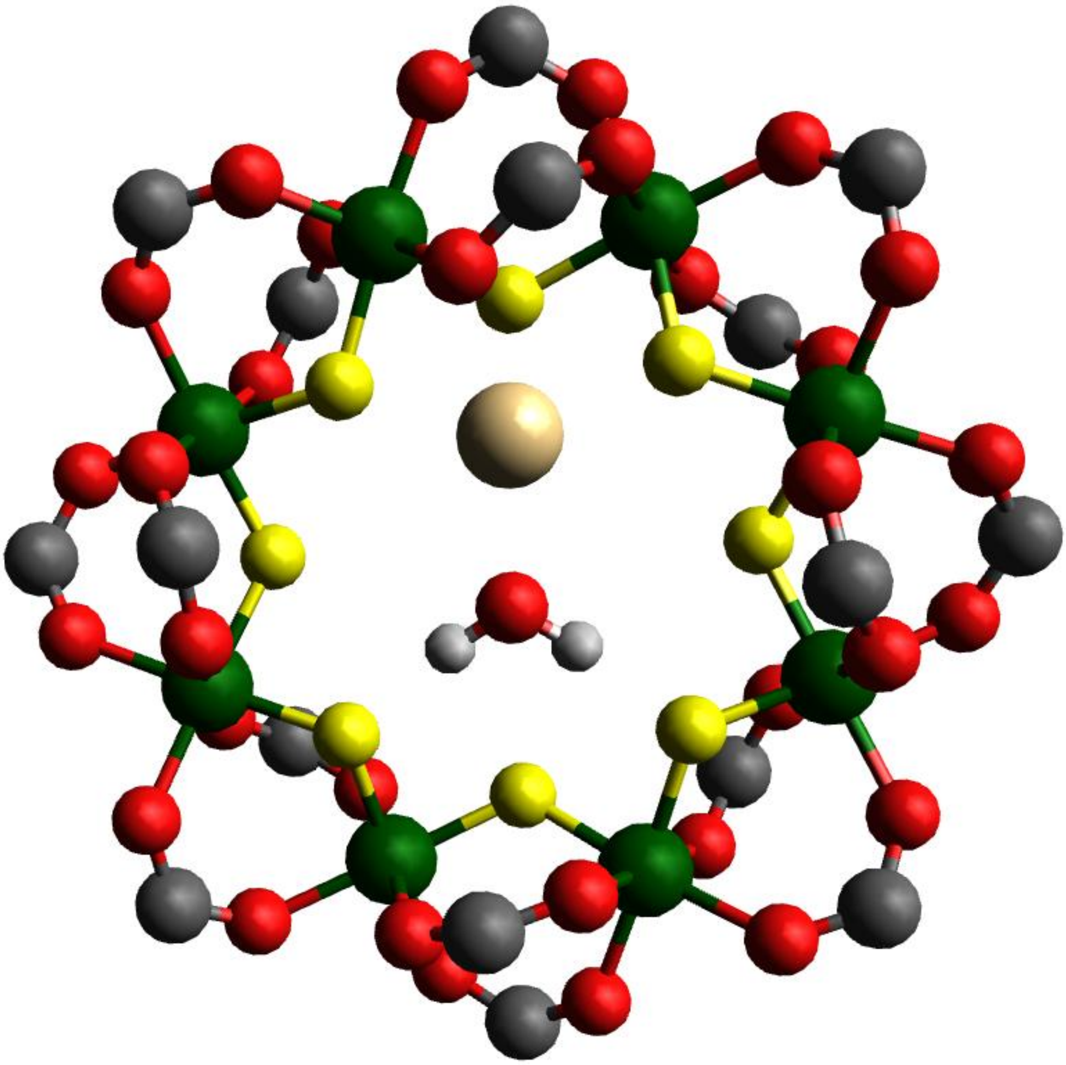

Figure S32: DFT optimised structure of [**1** + Cd + H_2O]$^{2+}$ (Cd: gold, F: yellow, Cr: dark green, C: black, O: red, H: white). *Tert*-butyl groups were omitted for clarity. Optimised coordinates, ESP charges and the minimum energy can be found in the Supplementary Dataset.

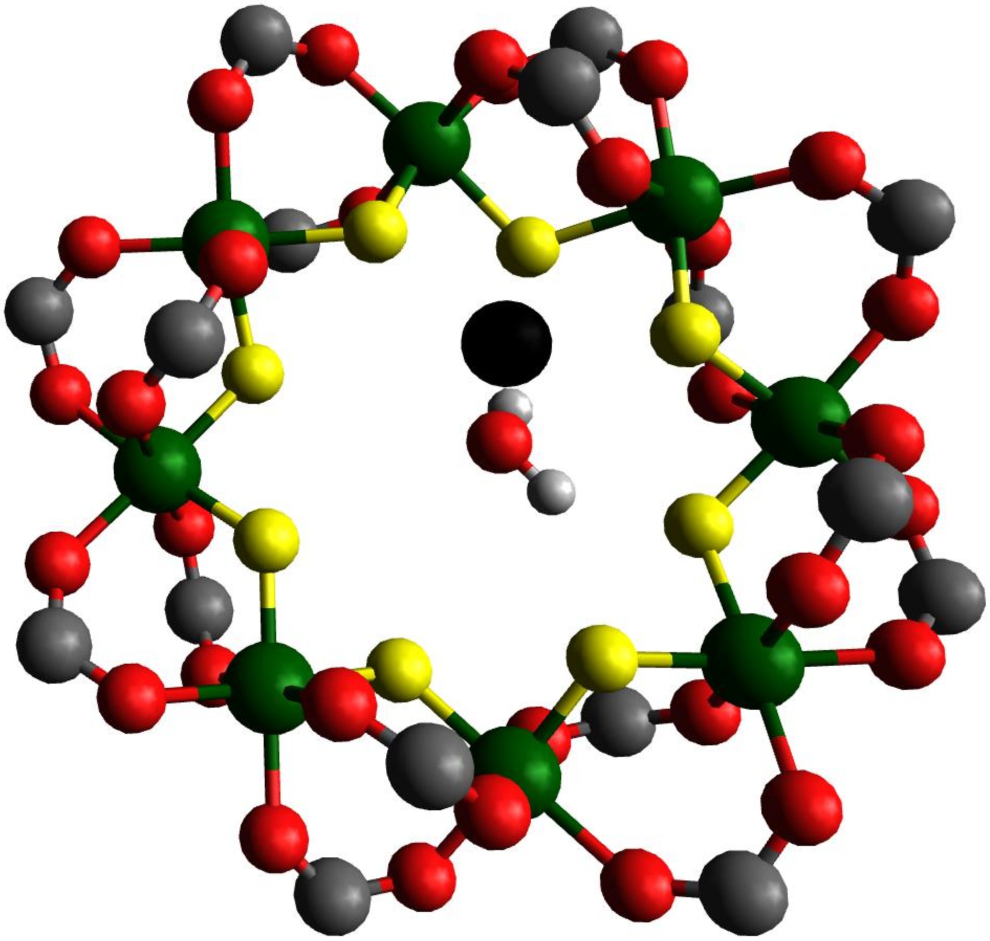

Figure S33: DFT optimised structure of [**1** + Sn + H_2O]$^{2+}$ (Sn: dark black, F: yellow, Cr: dark green, C: black, O: red, H: white). *Tert*-butyl groups were omitted for clarity. Optimised coordinates, ESP charges and the minimum energy can be found in the Supplementary Dataset.

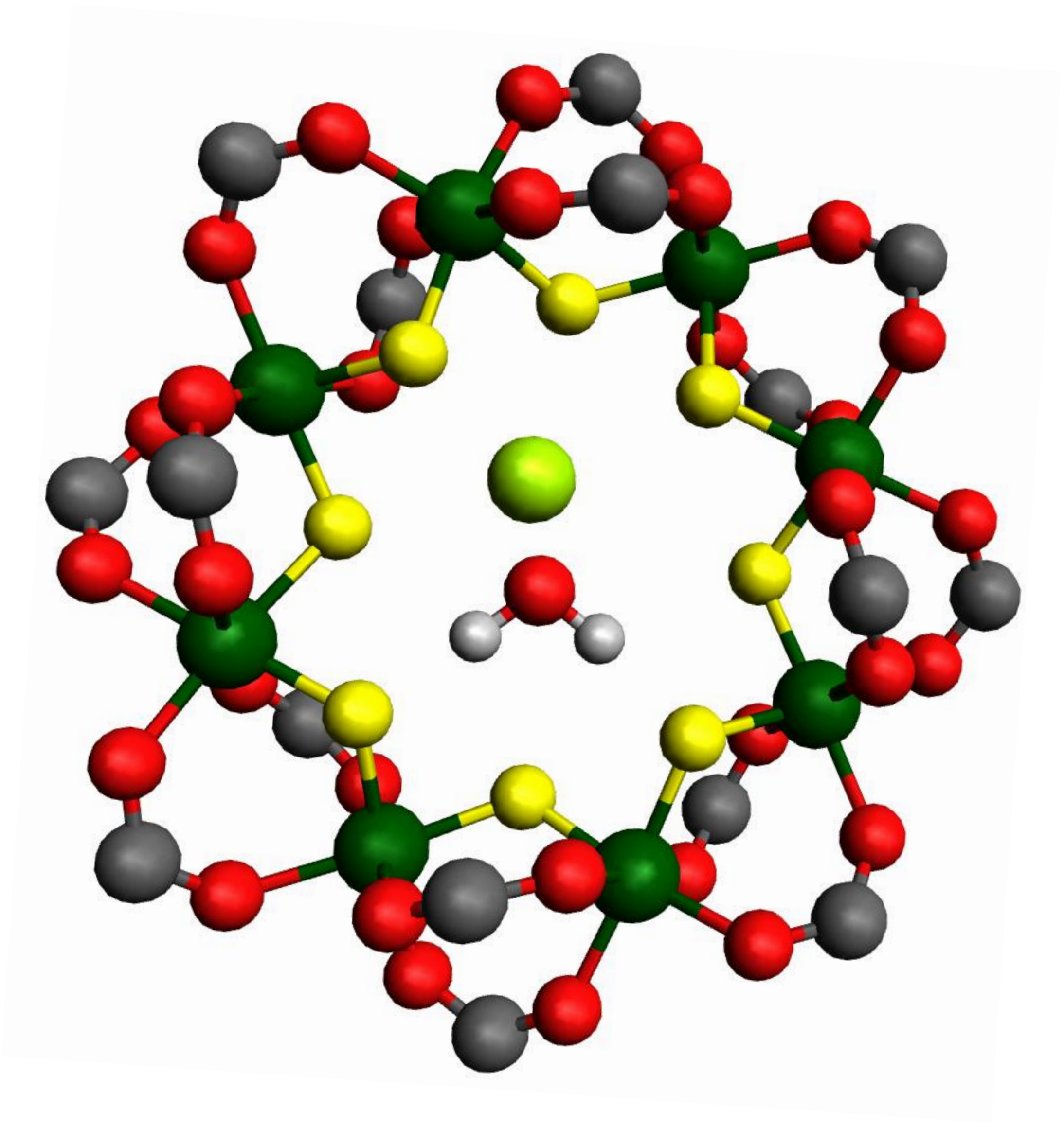

Figure S34: DFT optimised structure of [**1** + Pb + H_2O]$^{2+}$ (Pb: light green, F: yellow, Cr: dark green, C: black, O: red, H: white). *Tert*-butyl groups were omitted for clarity. Optimised coordinates, ESP charges and the minimum energy can be found in the Supplementary Dataset.

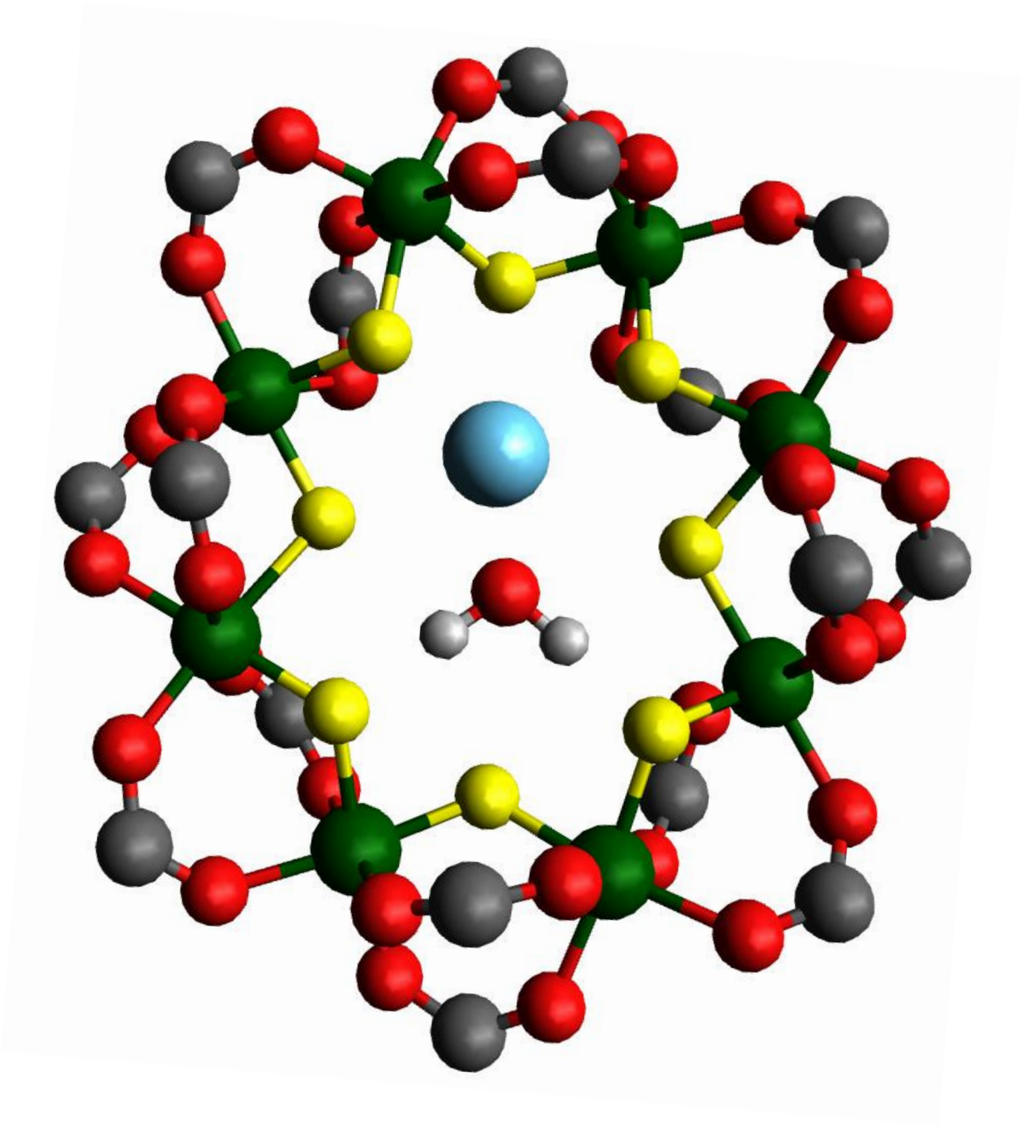

Figure S35: DFT optimised structure of [**1** + La + H_2O]$^{3+}$ (La: cyan, F: yellow, Cr: dark green, C: black, O: red, H: white). *Tert*-butyl groups were omitted for clarity. Optimised coordinates, ESP charges and the minimum energy can be found in the Supplementary Dataset.

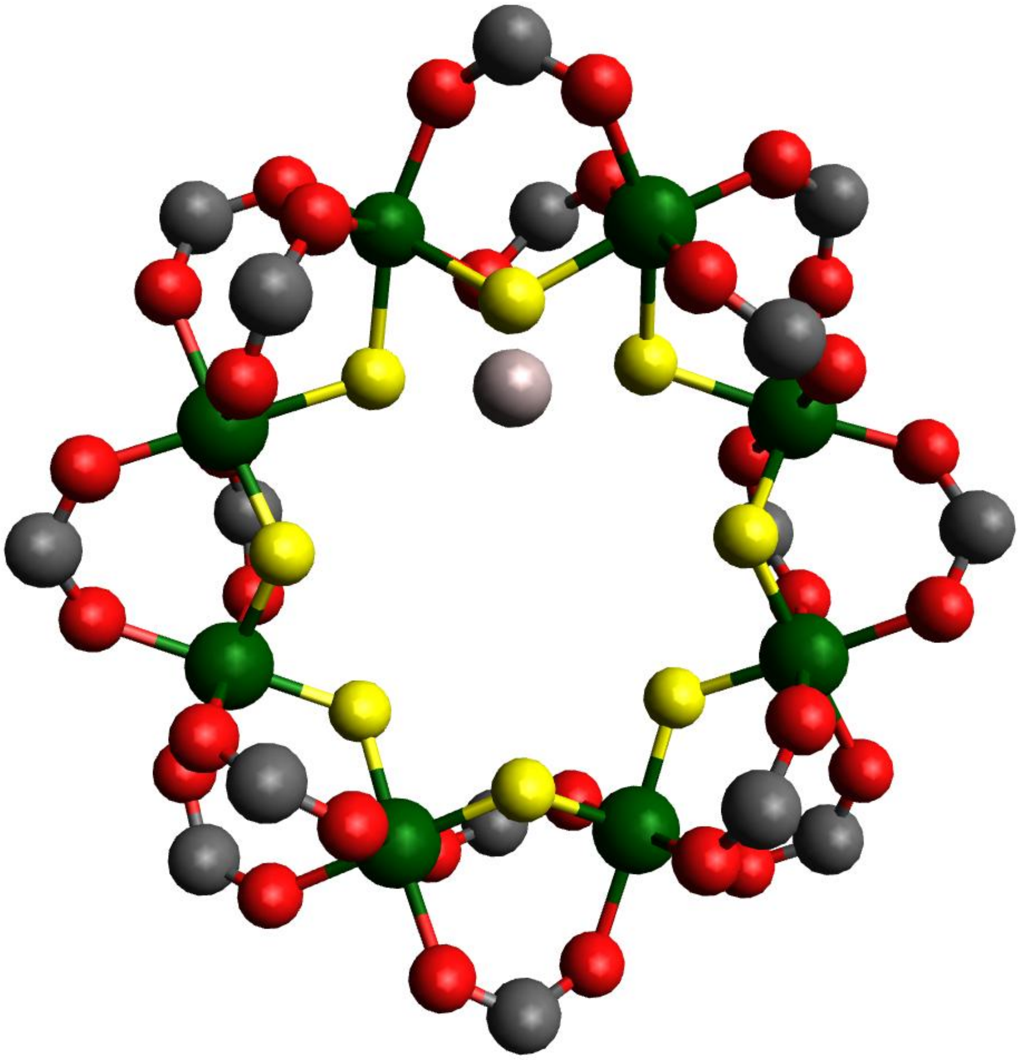

Figure S36: DFT optimised structure of [**1** + Al]$^{3+}$ (Al: grey, F: yellow, Cr: dark green, C: black, O: red, H: white). *Tert*-butyl groups were omitted for clarity. Optimised coordinates, ESP charges and the minimum energy can be found in the Supplementary Dataset.

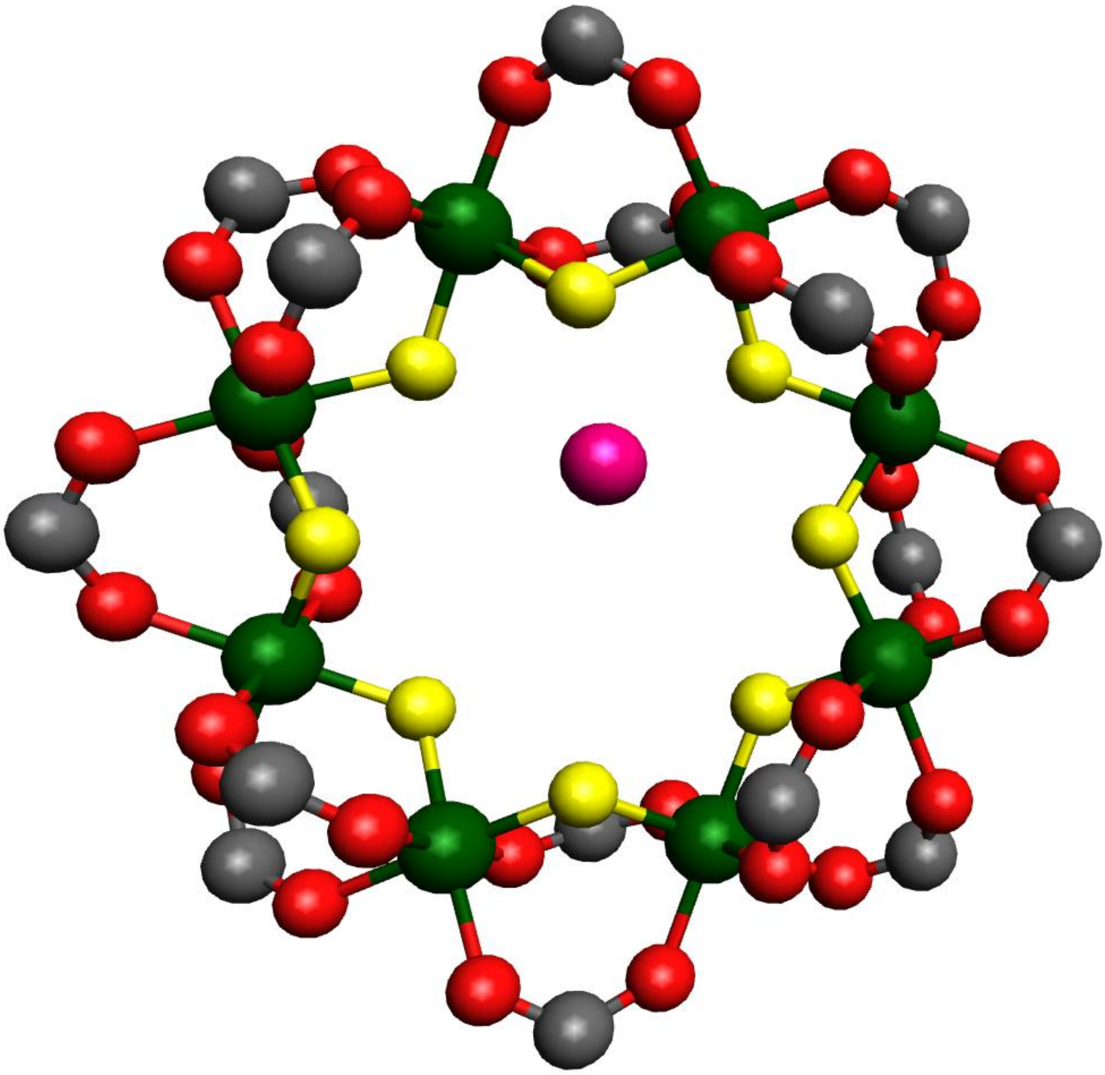

Figure S37: DFT optimised structure of [**1** + Ga]$^{3+}$ (Ga: magenta, F: yellow, Cr: dark green, C: black, O: red, H: white). *Tert*-butyl groups were omitted for clarity. Optimised coordinates, ESP charges and the minimum energy can be found in the Supplementary Dataset.

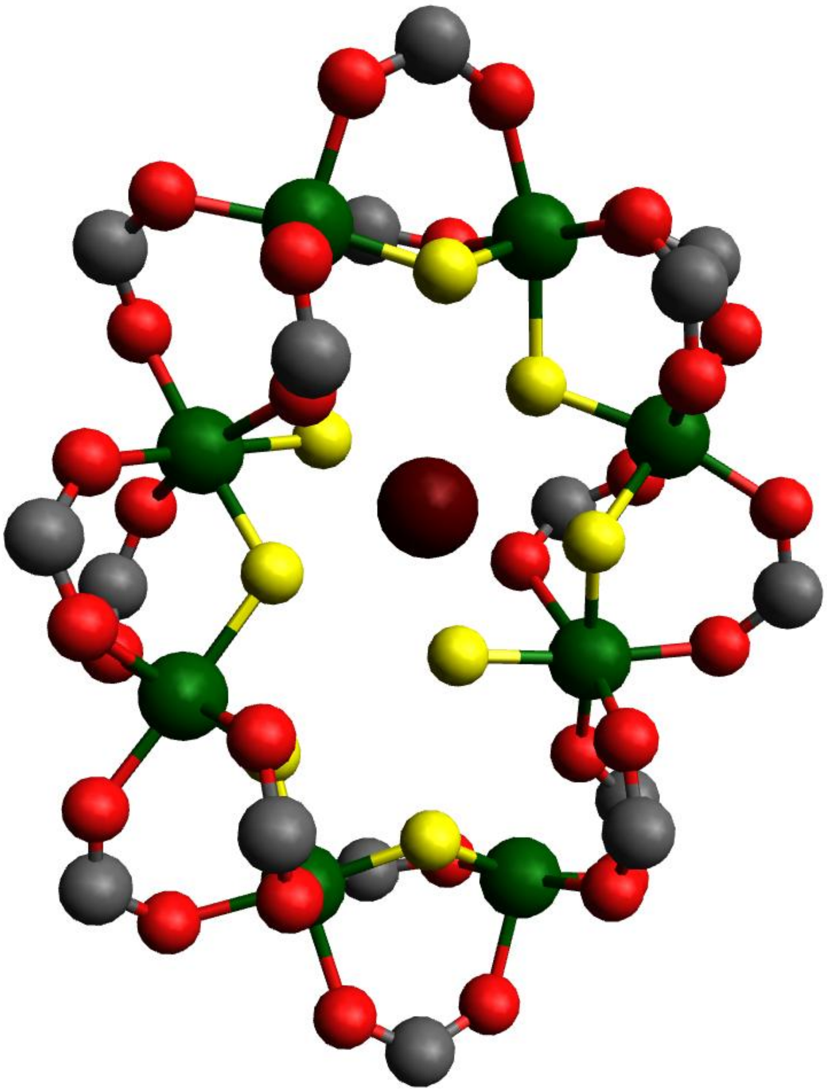

Figure S38: DFT optimised structure of [**1** + Y]$^{3+}$ (Y: dark brown, F: yellow, Cr: dark green, C: black, O: red, H: white). *Tert*-butyl groups were omitted for clarity. Optimised coordinates, ESP charges and the minimum energy can be found in the Supplementary Dataset.

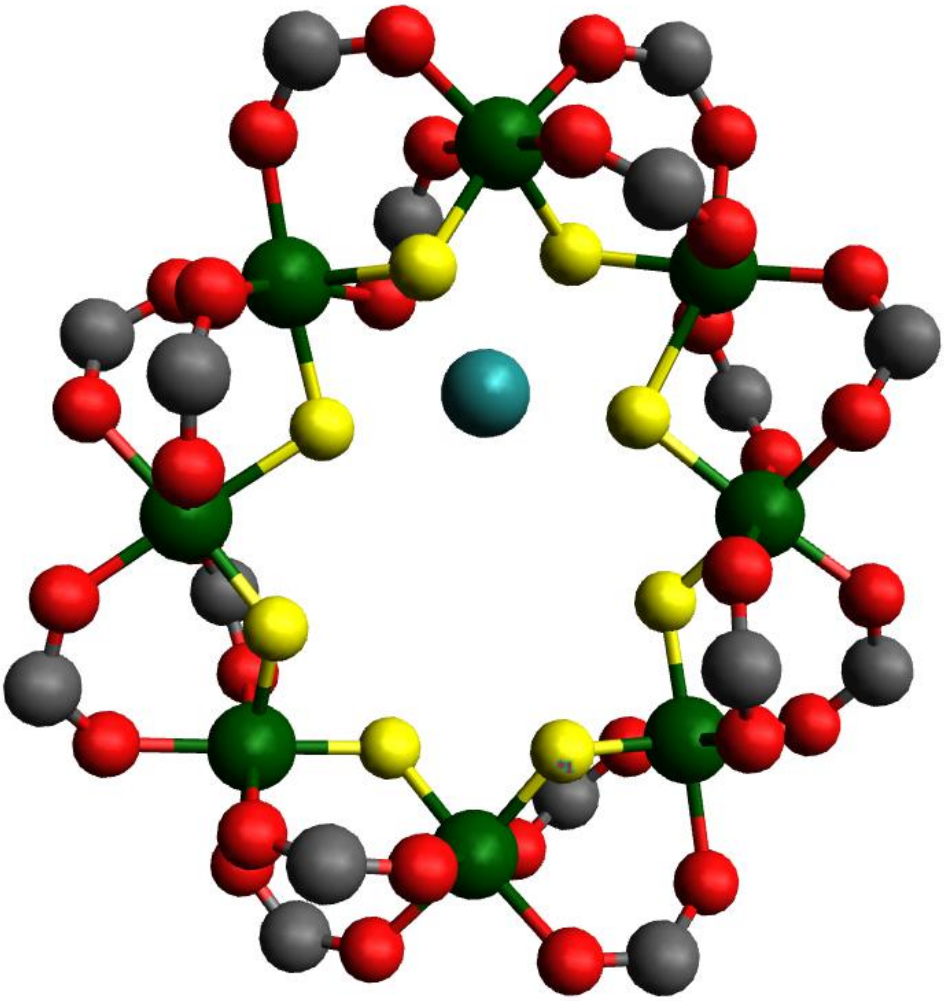

Figure S39: DFT optimised structure of [**1** + Ru]$^{3+}$ (Ru: petrol, F: yellow, Cr: dark green, C: black, O: red, H: white). *Tert*-butyl groups were omitted for clarity. Optimised coordinates and the minimum energy can be found in the Supplementary Dataset.

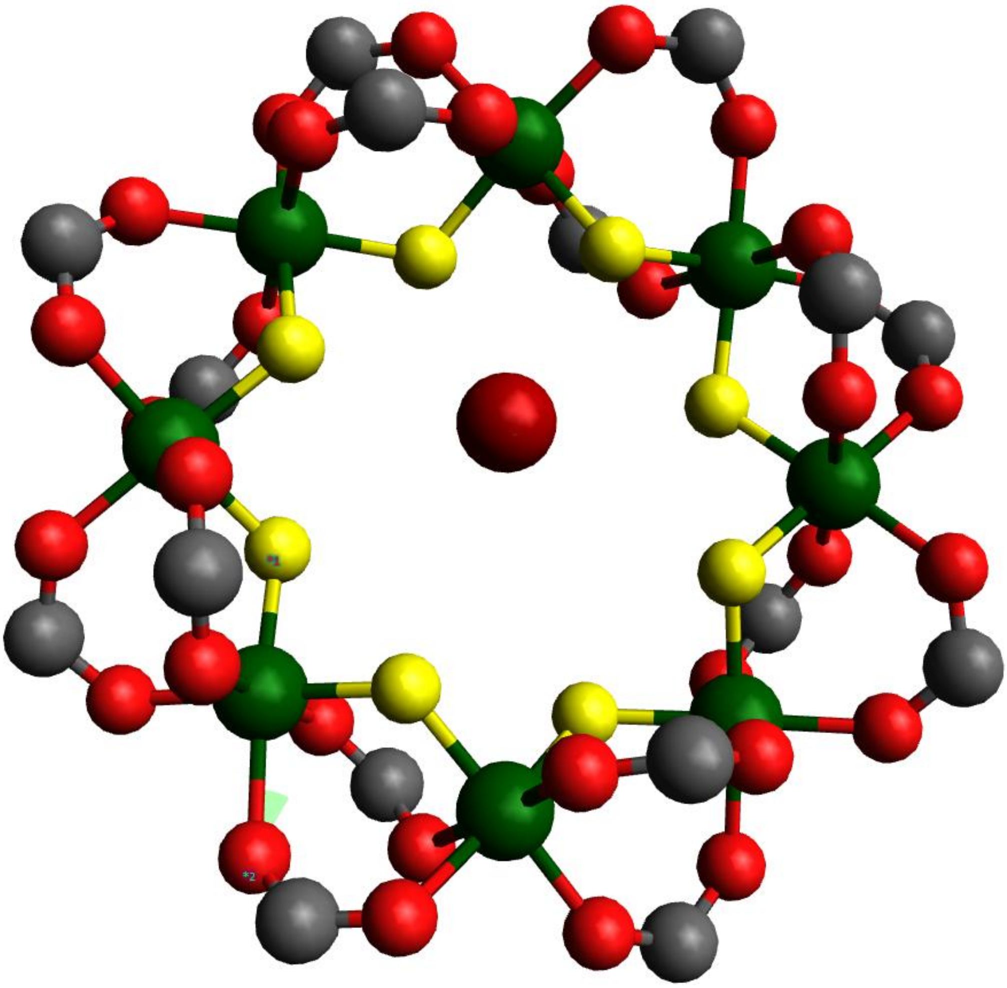

Figure S40: DFT optimised structure of [**1** + In]$^{3+}$ (In: dark red, F: yellow, Cr: dark green, C: black, O: red, H: white). *Tert*-butyl groups were omitted for clarity. Optimised coordinates, ESP charges and the minimum energy can be found in the Supplementary Dataset.

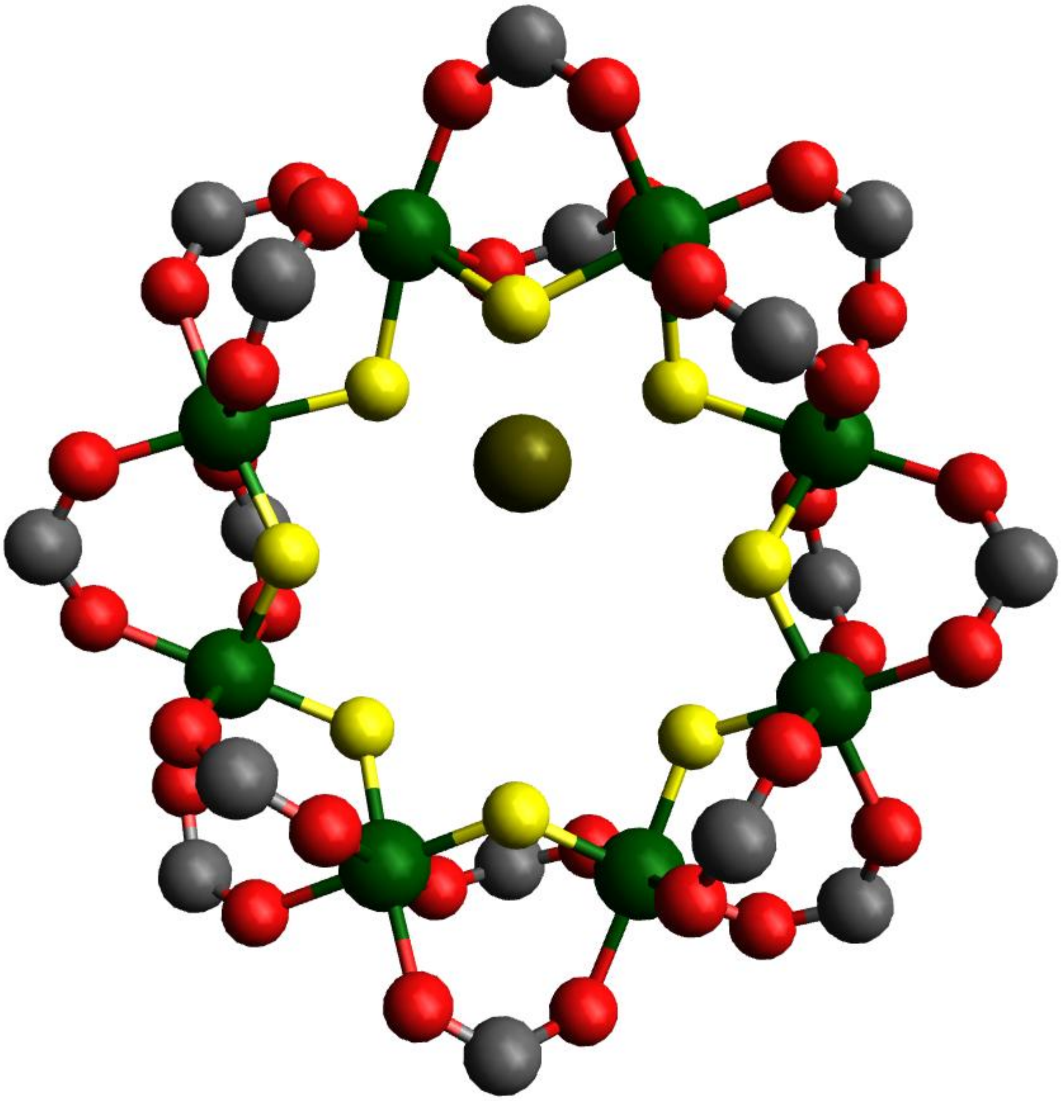

Figure S41: DFT optimised structure of [**1** + Sb]$^{3+}$ (Sb: olive, F: yellow, Cr: dark green, C: black, O: red, H: white). *Tert*-butyl groups were omitted for clarity. Optimised coordinates, ESP charges and the minimum energy can be found in the Supplementary Dataset.

Table S2: Relative binding energies of M to **1** in the DFT optimised structures of [**1** + M]$^{x+}$ that were experimentally observed (top part) and those that were not (bottom part).

**Metal M**	**Relative Binding Energy (kJ mol^{-1})**
Fe^{II}	1291
Co^{II}	1342
Ni^{II}	1409
Zn^{II}	1357
Cd^{II}	1172
Sn^{II}	1068
Pb^{II}	998
La^{III}	1965
Al^{III}	3099
Ga^{III}	3253
Y^{III}	2345
Ru^{III}	2773
In^{III}	2877
Sb^{III}	2548

Table S3: Eight M-F distances (Å) in the DFT optimised structures of the experimentally observed [**1** + M]$^{x+}$ species.

Fe^{II}	Co^{II}	Ni^{II}	Zn^{II}	Cd^{II}	Sn^{II}	Pb^{II}	La^{III}
1.951	1.967	1.948	1.932	2.496	2.284	2.373	2.542
1.998	2.019	1.967	1.980	2.518	2.313	2.467	2.551
2.149	2.072	1.991	2.077	2.811	2.346	2.489	2.605
3.862	3.978	3.821	3.888	2.895	3.504	3.006	2.606
3.919	3.995	3.971	3.960	3.525	3.848	3.686	2.764
4.813	4.940	4.875	4.895	3.618	4.190	4.002	2.771
4.891	5.056	4.947	4.977	4.059	4.408	4.429	4.062
5.489	5.666	5.496	5.528	4.083	4.801	4.561	4.072

Table S4: Eight M-F distances (Å) in the DFT optimised structures of the not experimentally observed [**1** + M]$^{3+}$ species.

**Al^{III}**	**Ga^{III}**	**Y^{III}**	**Ru^{III}**	**In^{III}**	**Sb^{III}**
1.871	2.270	2.135	2.142	2.680	2.137
1.885	2.411	2.136	2.269	2.684	2.217
1.936	2.758	2.380	2.306	2.963	2.232
3.824	3.120	2.392	2.307	3.037	3.328
3.948	3.556	2.423	3.879	3.408	3.408
4.910	3.894	3.355	4.331	3.506	4.179
4.987	4.117	4.209	5.008	3.789	4.245
5.523	4.375	4.726	5.132	3.950	4.619

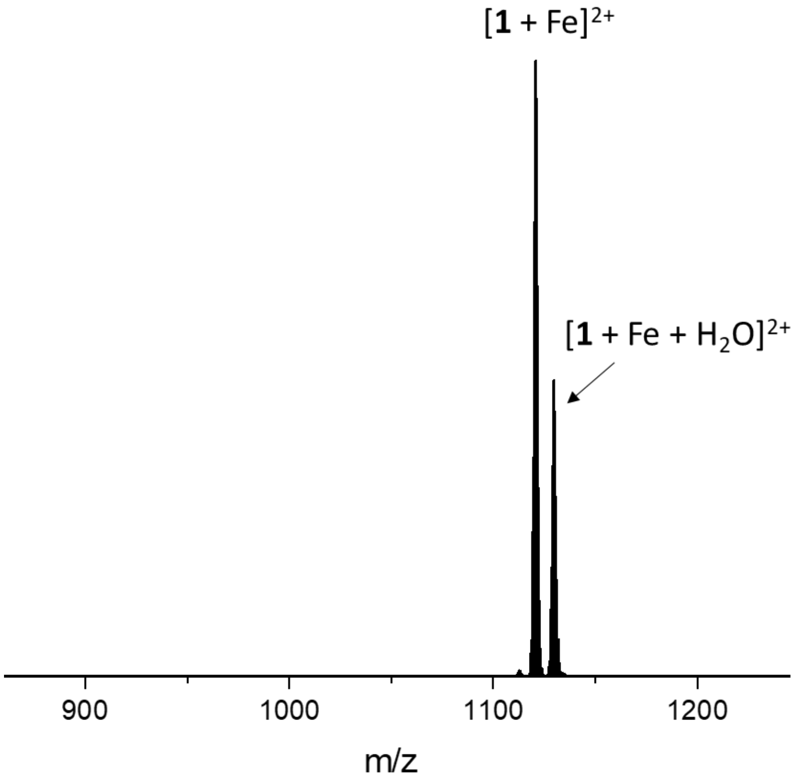

Figure S42: MS^2 spectrum of [**1** + Fe + H_2O]$^{2+}$ at E_{lab} = 60 eV. The single water adduct of [**1** + Fe + H_2O]$^{2+}$ readily dissociates via the loss of water to [**1** + Fe]$^{2+}$, a behaviour found for all host-guest-complex water adducts studied (Supplementary Dataset). For some M, the direct water loss is metastable, as an increase in intensity of [**1** + M]$^{x+}$ is not observed upon increasing the collision energy for the fragmentation of [**1** + M + H_2O]$^{x+}$. However, in all cases, the ring disassembly products do not contain water, indicating that water is lost upon collisional activation in the first step.

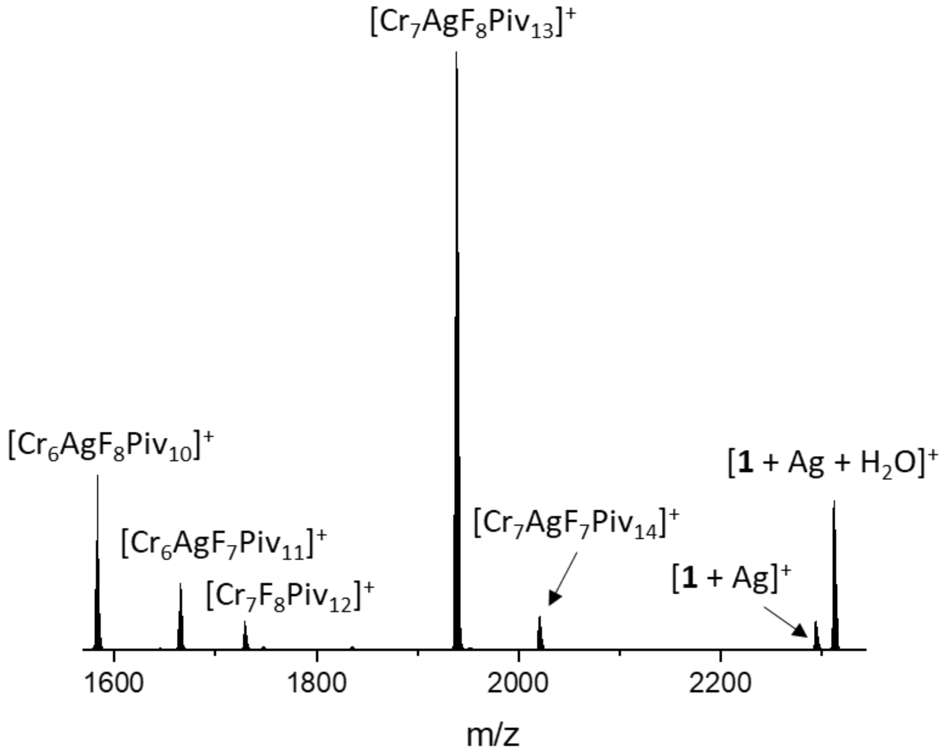

Figure S43: MS^2 spectrum of [**1** + Ag + H_2O]$^+$ at E_{lab} = 155 eV. After the initial loss of water, the disassembly proceeds via the loss of one Cr centre, mainly to $[Cr_7AgF_8Piv_{13}]^+$. In the secondary fragmentation step, both the loss of a second Cr centre and the dissociation of the Ag centre occur, along with the respective, charge-balancing number of ligands.

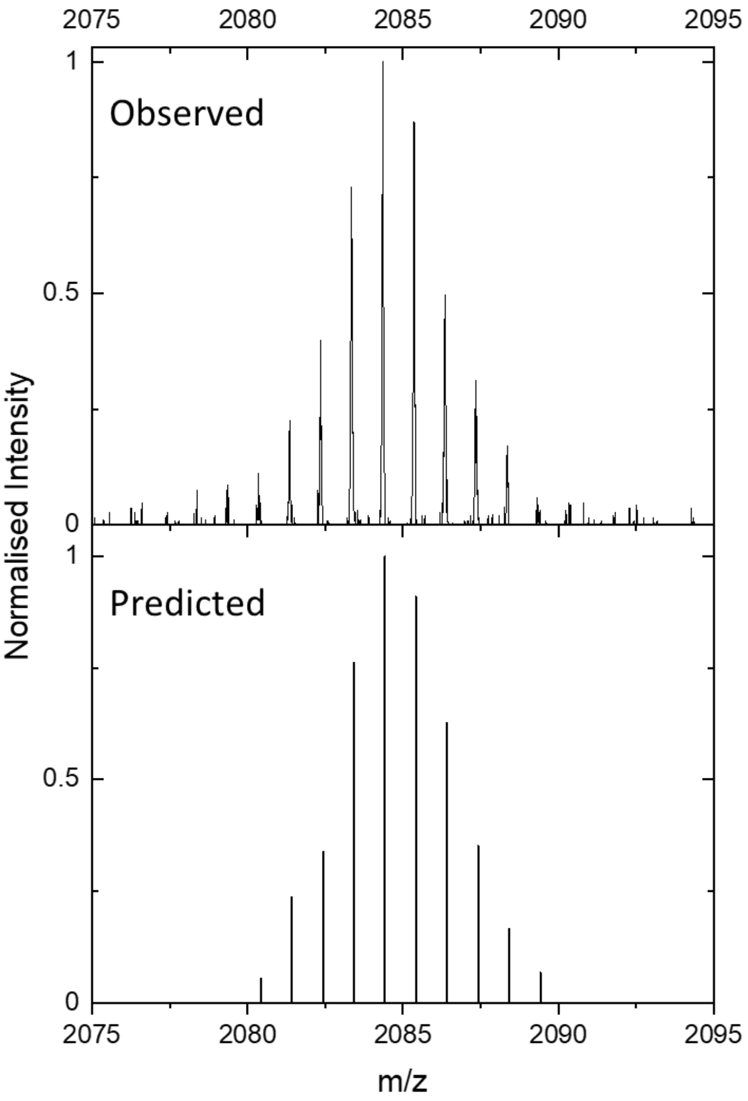

Figure S44: Comparison between observed and predicted isotopic distribution of $[Cr_8F_8Piv_{15}]^+$, obtained after fragmentation of [**1** + Pb]$^{2+}$ at E_{lab} = 120 eV.

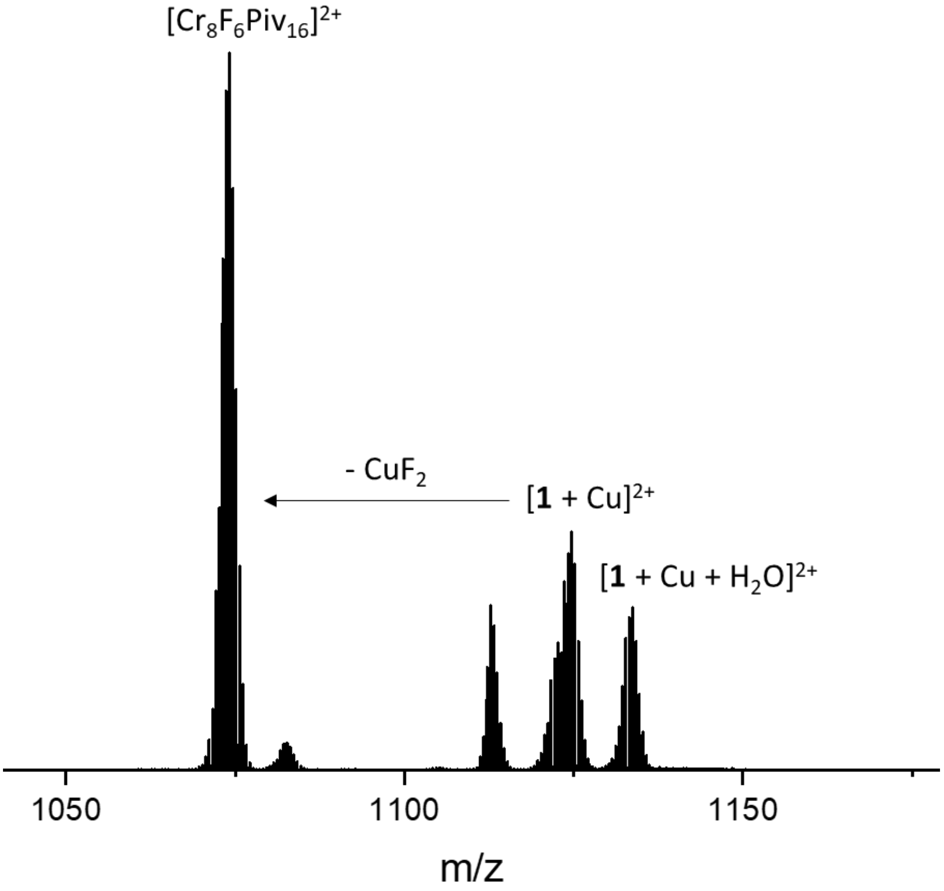

Figure S45: MS^2 spectrum of [**1** + Cu + H_2O]$^{2+}$ at E_{lab} = 80 eV. After the initial loss of water, the disassembly proceeds *via* the loss of CuF_2. Other visible ions are likely contaminations that result from fragmentation of overlapping ions during *m/z*-selection.

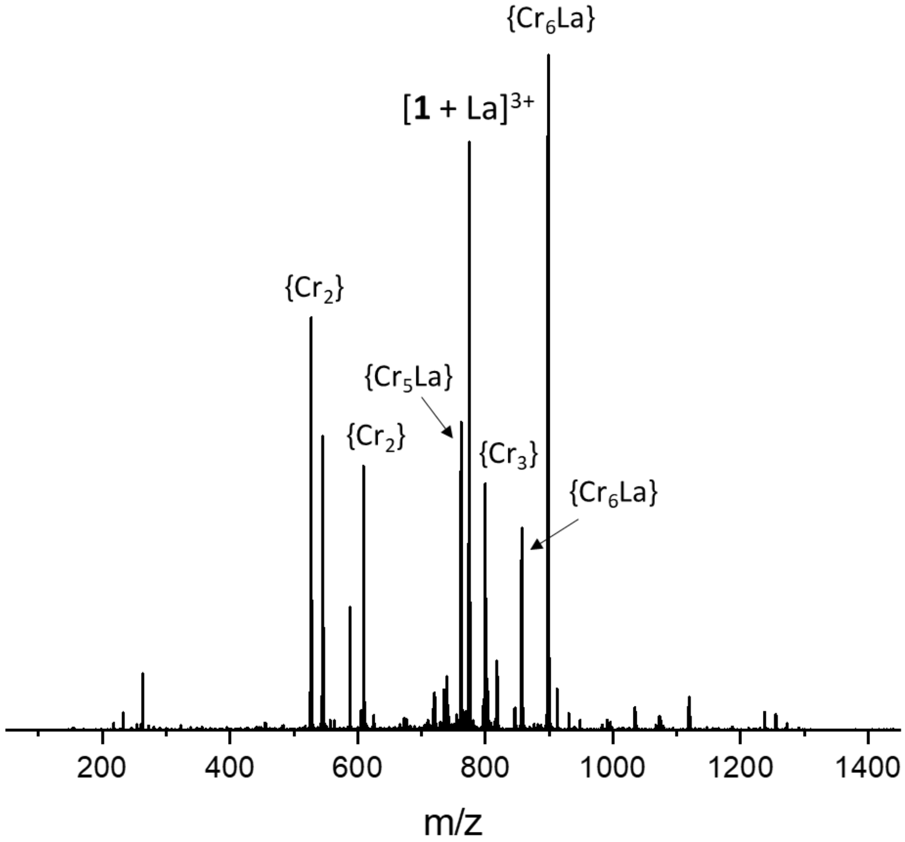

Figure S46: MS^2 spectrum of [**1** + La]$^{3+}$ at E_{lab} = 99 eV. Fragmentation of $\{Cr_8La\}^{3+}$ proceeds primarily to $\{Cr_6La\}^{2+}$ and $\{Cr_2\}^{+}$ as well as $\{Cr_5La\}^{2+}$ and $\{Cr_3\}^{+}$. Each ion includes also an appropriate number of anionic F^- and Piv^- ligands, which are not included in the labels for clarity.

Table S5: Composition, m/z and $^{TW}CCS_{N2}$ of selected homometallic fragments of [**1** + M]$^{x+}$ and [**1** + M + H_2O]$^{x+}$.

Stoichiometry	m/z	$^{TW}CCS_{N2}$ (Å^2)
$[Cr_8F_6Piv_{16}]^{2+}$	1073.7	438.9 ± 0.7
$[Cr_8F_8Piv_{15}]^+$	2084.4	423.8 ± 0.4
$[Cr_7F_8Piv_{12}]^+$	1729.3	380.1 ± 0.9
$[Cr_6F_6Piv_{11}]^+$	1538.3	353.0 ± 0.8<br>373.3 ± 0.9<br>382.6 ± 0.4
$[Cr_5F_5Piv_9]^+$	1264.2	322.2 ± 0.9<br>334.7 ± 0.9<br>341.4 ± 0.7
$[Cr_4F_3Piv_8]^+$	1073.2	299.4 ± 0.9<br>308.6 ± 0.7
$[Cr_3F_2Piv_6]^+$	800.2	266.7 ± 0.9
$[Cr_2Piv_5]^+$	609.2	241.1 ± 1.4

Table S6: Composition, m/z and $^{TW}CCS_{N2}$ of selected heterometallic fragments of [**1** + M]$^{x+}$ and [**1** + M + H_2O]$^{x+}$.

**Stoichiometry**	**m/z**	**$^{TW}CCS_{N2}$ (Å^2)**
$[Cr_7AgF_7Piv_{14}]^+$	2020.3	395.3 ± 0.1
$[Cr_7AgF_8Piv_{13}]^+$ and $[Cr_7MF_8Piv_{13}]^{2+}$ (M^{2+})	Ag^+: 1938.3	Ag^+: 389.6 ± 0.1
	Fe^{2+}: 943.1	Fe^{2+}: 408.6 ± 0.6
	Co^{2+}: 944.6	Co^{2+}: 408.3 ± 0.6
	Ni^{2+}: 944.6	Ni^{2+}: 405.2 ± 0.6
	Zn^{2+}: 947.6	Zn^{2+}: 406.8 ± 0.6
	Cd^{2+}: 971.6	Cd^{2+}: 407.1 ± 0.6
	Sn^{2+}: 974.6	Sn^{2+}: 414.9 ± 0.6
	Pb^{2+}: 1018.7	Pb^{2+}: 413.8 ± 0.6
$[Cr_6LnF_7Piv_{12}]^{2+}$	La^{3+}: 899.1	La^{3+}: 401.3 ± 0.6
	Gd^{3+}: 908.1	Gd^{3+}: 400.8 ± 0.6
	Yb^{3+}: 916.1	Yb^{3+}: 400.3 ± 0.6
$[Cr_6MF_8Piv_{11}]^+$ (M^{2+}) and $[Cr_6MF_8Piv_{11}]^{2+}$ (M^{3+})	Fe^{2+}: 1632.2	Fe^{2+}: 359.9 ± 0.2 & 367.4 ± 0.2
	Co^{2+}: 1635.2	Co^{2+}: 358.2 ± 0.2 & 365.8 ± 0.2
	Ni^{2+}: 1634.2	Ni^{2+}: 358.6 ± 0.1 & 366.1 ± 0.1
	Zn^{2+}: 1641.2	Zn^{2+}: 359.5 ± 0.1 & 366.7 ± 0.1
	Cd^{2+}: 1689.2	Cd^{2+}: 358.6 ± 0.7 & 366.5 ± 0.5
	Sn^{2+}: 1695.2	Sn^{2+}: 363.9 ± 0.1 & 372.2 ± 0.1 & 395.0 ± 0.1
	Pb^{2+}: 1783.3	Pb^{2+}: 362.9 ± 0.2 & 371.9 ± 0.1

	La^{3+}: 857.6<br>Gd^{3+}: 866.6<br>Yb^{3+}: 874.6	La^{3+}: 397.8 ± 0.6<br>Gd^{3+}: 396.0 ± 0.6<br>Yb^{3+}: 395.5 ± 0.6
$[Cr_6AgF_8Piv_{10}]^+$	1583.1	350.7 ± 0.1
$[Cr_5MF_6Piv_{10}]^+$ (M^{2+}) and $[Cr_5MF_6Piv_{10}]^{2+}$ (M^{3+})	Fe^{2+}: 1441.2<br>Co^{2+}: 1444.2<br>Ni^{2+}: 1443.2<br>Zn^{2+}: 1450.2<br>Cd^{2+}: 1498.2<br>Sn^{2+}: 1504.2<br>Pb^{2+}: 1592.3<br>La^{3+}: 762.1<br>Gd^{3+}: 771.1<br>Yb^{3+}: 779.1	Fe^{2+}: 343.2 ± 0.3<br>Co^{2+}: 341.7 ± 0.3<br>Ni^{2+}: 340.7 ± 0.1<br>Zn^{2+}: 342.3 ± 0.1<br>Cd^{2+}: 344.4 ± 0.9<br>Sn^{2+}: 348.1 ± 0.1 & 363.3 ± 0.1<br>Pb^{2+}: 348.7 ± 0.3<br>La^{3+}: 375.3 ± 0.4<br>Gd^{3+}: 374.7 ± 0.7<br>Yb^{3+}: 373.6 ± 0.7
$[Cr_5AgF_7Piv_8]^+$	1310.1	321.5 ± 0.1
$[Cr_4MF_5Piv_8]^+$	Fe^{2+}: 1167.2<br>Co^{2+}: 1170.2<br>Ni^{2+}: 1169.2<br>Zn^{2+}: 1177.2<br>Cd^{2+}: 1225.1<br>Sn^{2+}: 1231.1<br>Pb^{2+}: 1319.2	Fe^{2+}: 311.8 ± 0.5<br>Co^{2+}: 311.7 ± 0.5<br>Ni^{2+}: 310.6 ± 0.2<br>Zn^{2+}: 312.5 ± 0.2<br>Cd^{2+}: 312.7 ± 0.4<br>Sn^{2+}: 320.4 ± 0.5<br>Pb^{2+}: 315.0 ± 0.5

$[Cr_4LnF_7Piv_7]^+$	La^{3+}: 1187.1<br>Gd^{3+}: 1206.1<br>Yb^{3+}: 1222.1	La^{3+}: 308.6 ± 0.1<br>Gd^{3+}: 309.9 ± 0.1<br>Yb^{3+}: 309.5 ± 0.1
$[Cr_4AgF_6Piv_6]^+$	1037.0	285.8 ± 0.2 & 291.5 ± 0.2
$[Cr_3MF_4Piv_6]^+$	Fe^{2+}: 894.1<br>Co^{2+}: 897.1<br>Ni^{2+}: 896.1<br>Zn^{2+}: 902.1<br>Cd^{2+}: 952.1<br>Sn^{2+}: 958.1<br>Pb^{2+}: 1046.2	Fe^{2+}: 280.7 ± 0.6<br>Co^{2+}: 279.8 ± 0.6<br>Ni^{2+}: 279.5 ± 0.3<br>Zn^{2+}: 280.5 ± 0.2<br>Cd^{2+}: 279.0 ± 0.5<br>Sn^{2+}: 280.4 ± 0.6<br>Pb^{2+}: 279.5 ± 0.6
$[Cr_3AgF_4Piv_5]^+$	846.0	262.3 ± 0.3 & 266.7 ± 0.3
$[Cr_3LnF_6Piv_5]^+$	La^{3+}: 914.0<br>Gd^{3+}: 933.0<br>Yb^{3+}: 949.1	La^{3+}: 270.3 ± 0.1<br>Gd^{3+}: 256.9 ± 0.1 & 269.7 ± 0.1<br>Yb^{3+}: 256.2 ± 0.1 & 270.2 ± 0.1
$[Cr_2MF_2Piv_5]^+$	Fe^{2+}: 703.1<br>Co^{2+}: 706.1<br>Ni^{2+}: 705.1<br>Zn^{2+}: 711.1<br>Cd^{2+}: 761.1<br>Sn^{2+}: 767.1	Fe^{2+}: 253.7 ± 0.7<br>Co^{2+}: 253.6 ± 0.7<br>Ni^{2+}: 253.7 ± 0.3<br>Zn^{2+}: 253.0 ± 0.3<br>Cd^{2+}: 252.8 ± 0.5<br>Sn^{2+}: 251.3 ± 0.1 & 256.0 ± 0.6

	Pb^{2+}: 855.2	Pb^{2+}: 251.5 ± 0.7 & 255.9 ± 0.7
$[Cr_2LnF_3Piv_5]^+$	La^{3+}: 805.1<br>Gd^{3+}: 824.1<br>Yb^{3+}: 840.1	La^{3+}: 251.3 ± 0.1 & 260.9 ± 0.1<br>Gd^{3+}: 250.6 ± 0.1 & 259.2 ± 0.1<br>Yb^{3+}: 250.5 ± 0.1 & 258.3 ± 0.1
$[Cr_2AgF_2Piv_4]^+$	653.0	238.3 ± 0.3

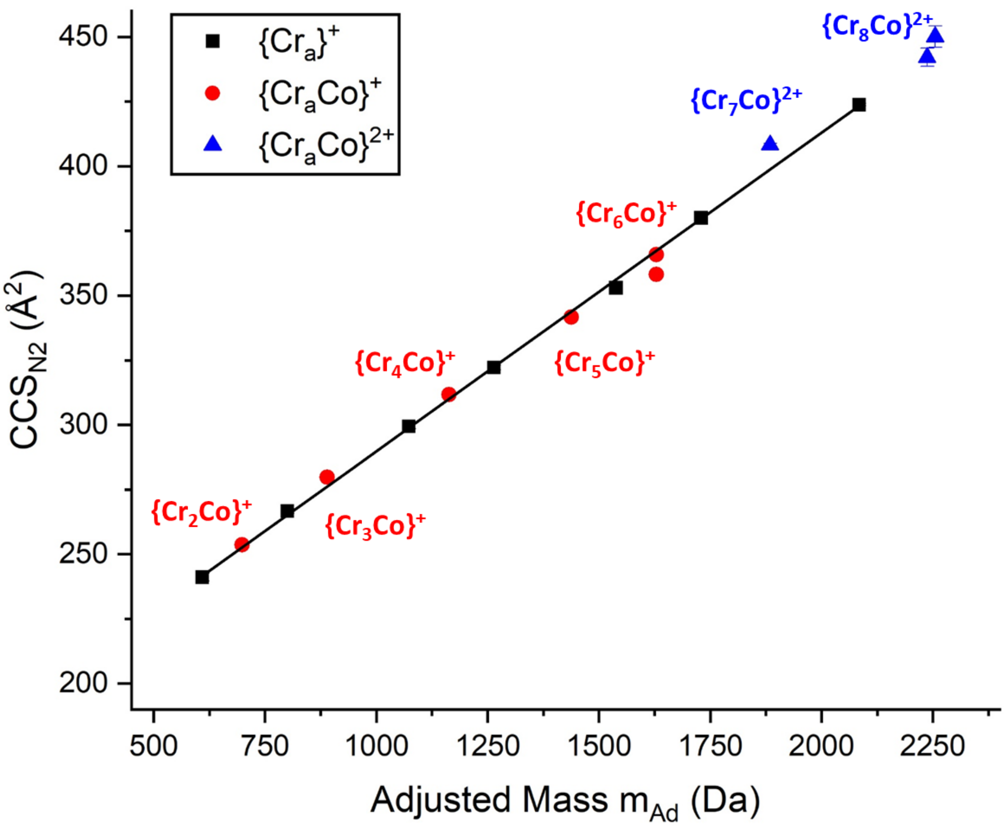

Figure S47: Correlation between CCS_{N2} and adjusted mass for $\{Cr_aCo\}$ ions. Data for singly charged homometallic ions (Table S5) are represented as black squares, whereas data for heterometallic ions are depicted as red circles (+1) and blue triangles (+2), respectively (Table S1 for host-guest complexes and Table S6 for fragments). The mass was adjusted so that every metal centre is accounted for with the mass of chromium (52 Da).

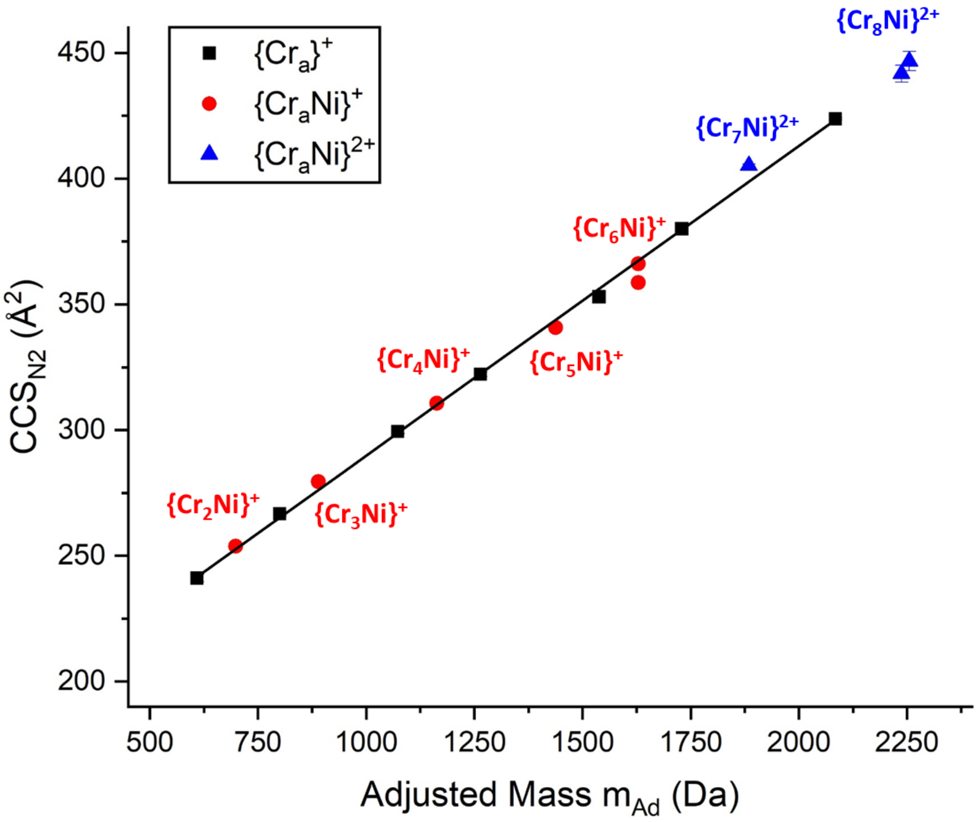

Figure S48: Correlation between CCS$_{N2}$ and adjusted mass for {Cr$_a$Ni} ions. Data for singly charged homometallic ions (Table S5) are represented as black squares, whereas data for heterometallic ions are depicted as red circles (+1) and blue triangles (+2), respectively (Table S1 for host-guest complexes and Table S6 for fragments). The mass was adjusted so that every metal centre is accounted for with the mass of chromium (52 Da).

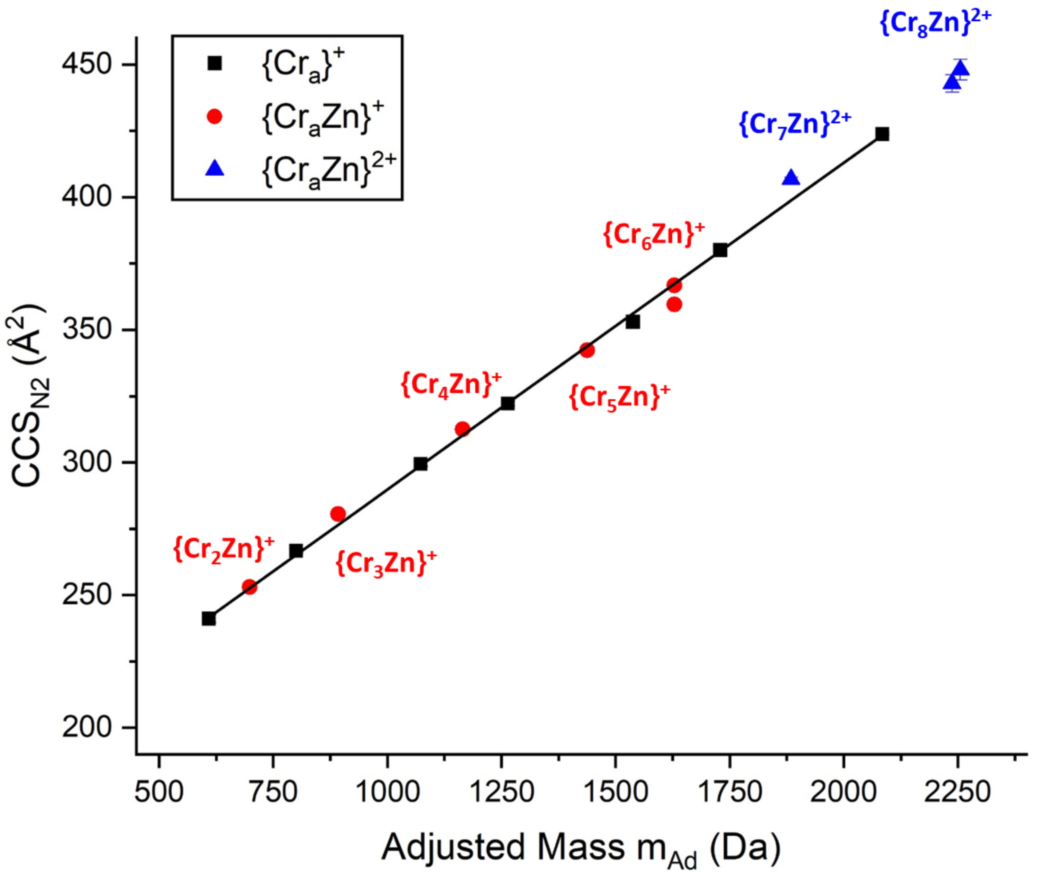

Figure S49: Correlation between CCS_{N2} and adjusted mass for {Cr_aZn} ions. Data for singly charged homometallic ions (Table S5) are represented as black squares, whereas data for heterometallic ions are depicted as red circles (+1) and blue triangles (+2), respectively (Table S1 for host-guest complexes and Table S6 for fragments). The mass was adjusted so that every metal centre is accounted for with the mass of chromium (52 Da).

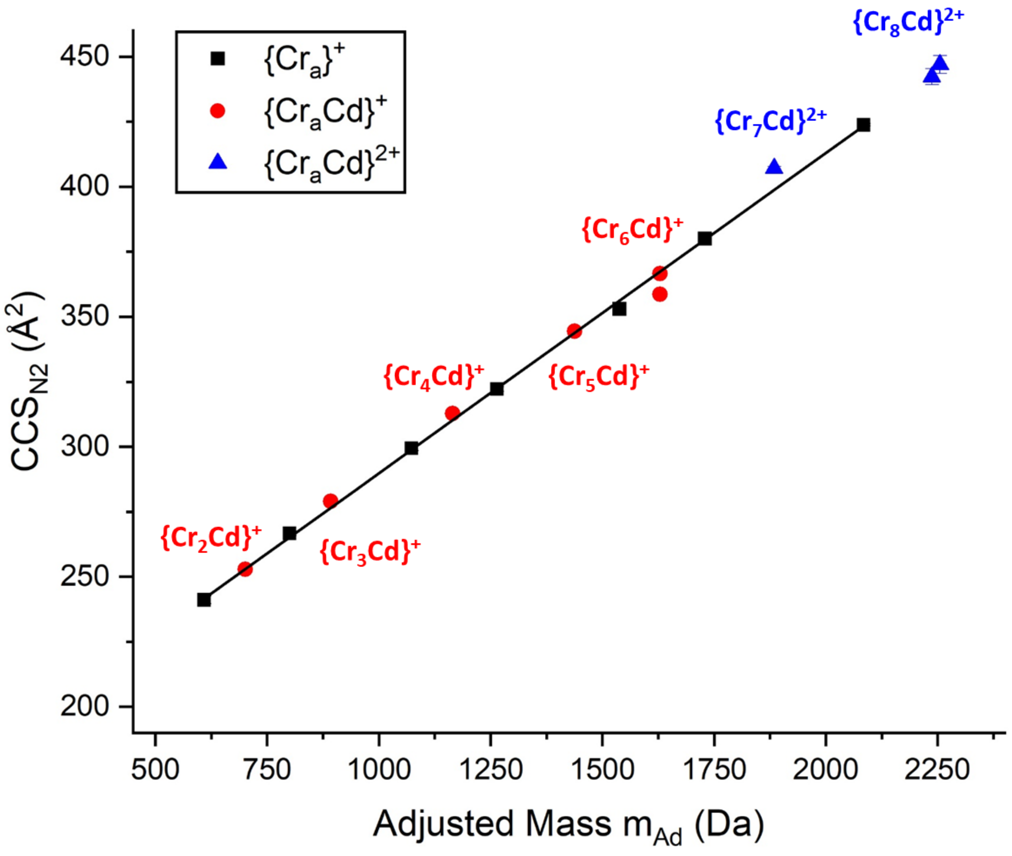

Figure S50: Correlation between CCS_{N2} and adjusted mass for {Cr_aCd} ions. Data for singly charged homometallic ions (Table S5) are represented as black squares, whereas data for heterometallic ions are depicted as red circles (+1) and blue triangles (+2), respectively (Table S1 for host-guest complexes and Table S6 for fragments). The mass was adjusted so that every metal centre is accounted for with the mass of chromium (52 Da).

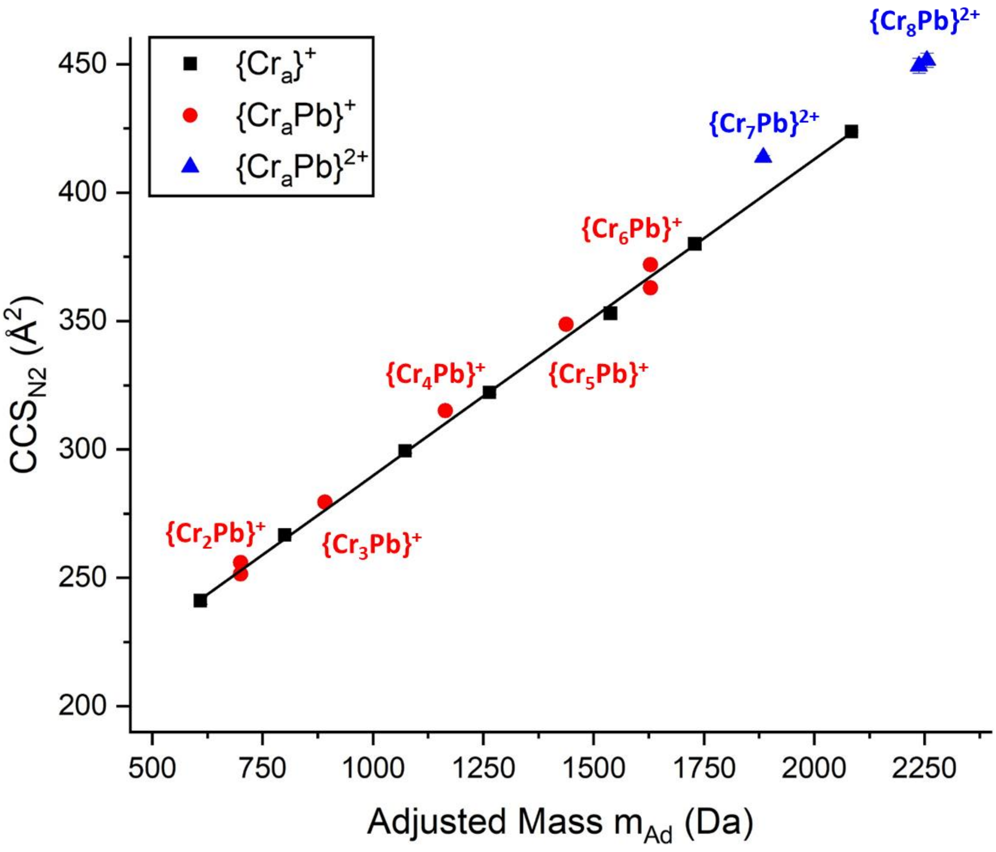

Figure S51: Correlation between CCS_{N2} and adjusted mass for {Cr_aPb} ions. Data for singly charged homometallic ions (Table S5) are represented as black squares, whereas data for heterometallic ions are depicted as red circles (+1) and blue triangles (+2), respectively (Table S1 for host-guest complexes and Table S6 for fragments). The mass was adjusted so that every metal centre is accounted for with the mass of chromium (52 Da).

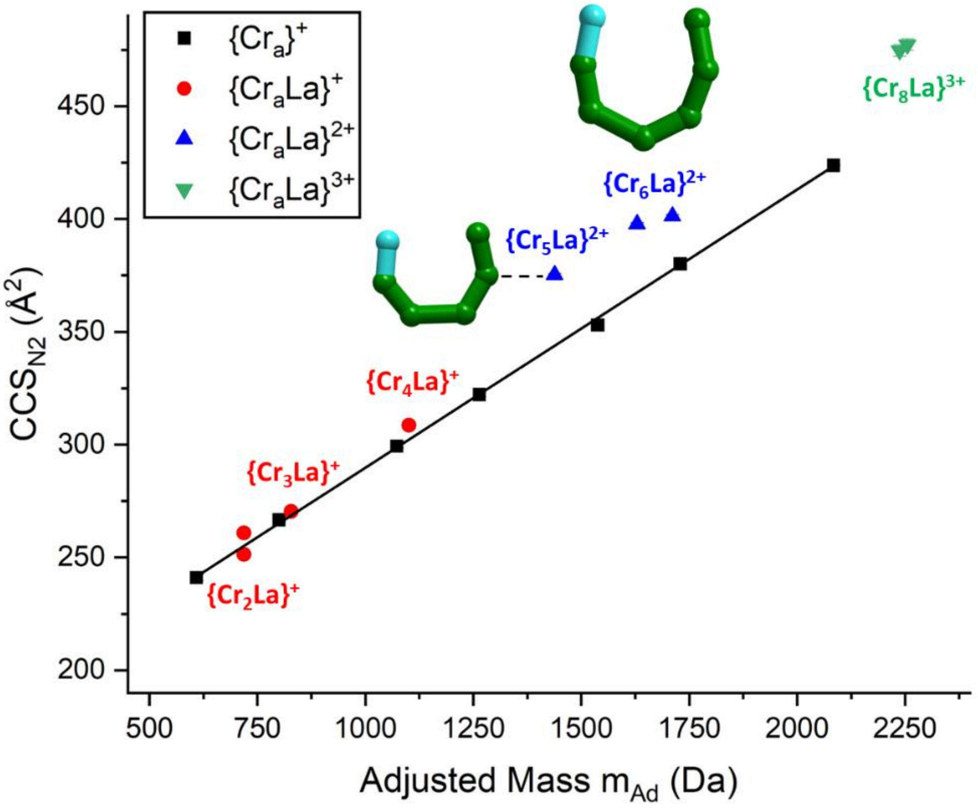

Figure S52: Correlation between CCS_{N2} and adjusted mass for $\{Cr_aLa\}$ ions. Data for singly charged homometallic ions (Table S5) are represented as black squares, whereas data for heterometallic ions are depicted as red circles (+1), blue upwards triangles (+2), green downwards triangles (+3), respectively (Table S1 for host-guest complexes and Table S6 for fragments). The mass was adjusted so that every metal centre is accounted for with the mass of chromium (52 Da). The species $\{Cr_5La\}^{2+}$ and $\{Cr_6La\}^{2+}$ were tentatively assigned to open chain structures as they deviate 6 – 9% from the trendline, however it is challenging to disentangle the charge effect reliably.

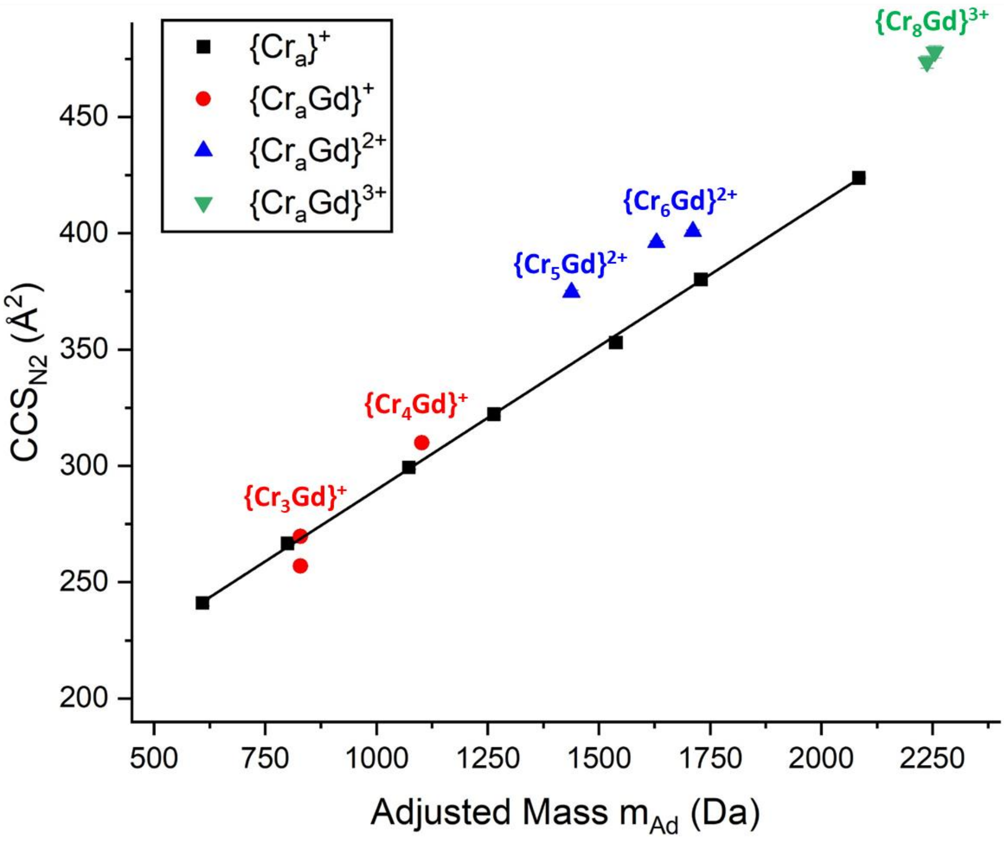

Figure S53: Correlation between CCS_{N2} and adjusted mass for {Cr_aGd} ions. Data for singly charged homometallic ions (Table S5) are represented as black squares, whereas data for heterometallic ions are depicted as red circles (+1), blue upwards triangles (+2), green downwards triangles (+3), respectively (Table S1 for host-guest complexes and Table S6 for fragments). The mass was adjusted so that every metal centre is accounted for with the mass of chromium (52 Da). The species {Cr_5Gd}$^{2+}$ and {Cr_6Gd}$^{2+}$ were tentatively assigned to open chain structures as they deviate 6 – 9% from the trendline, however it is challenging to disentangle the charge effect reliably.

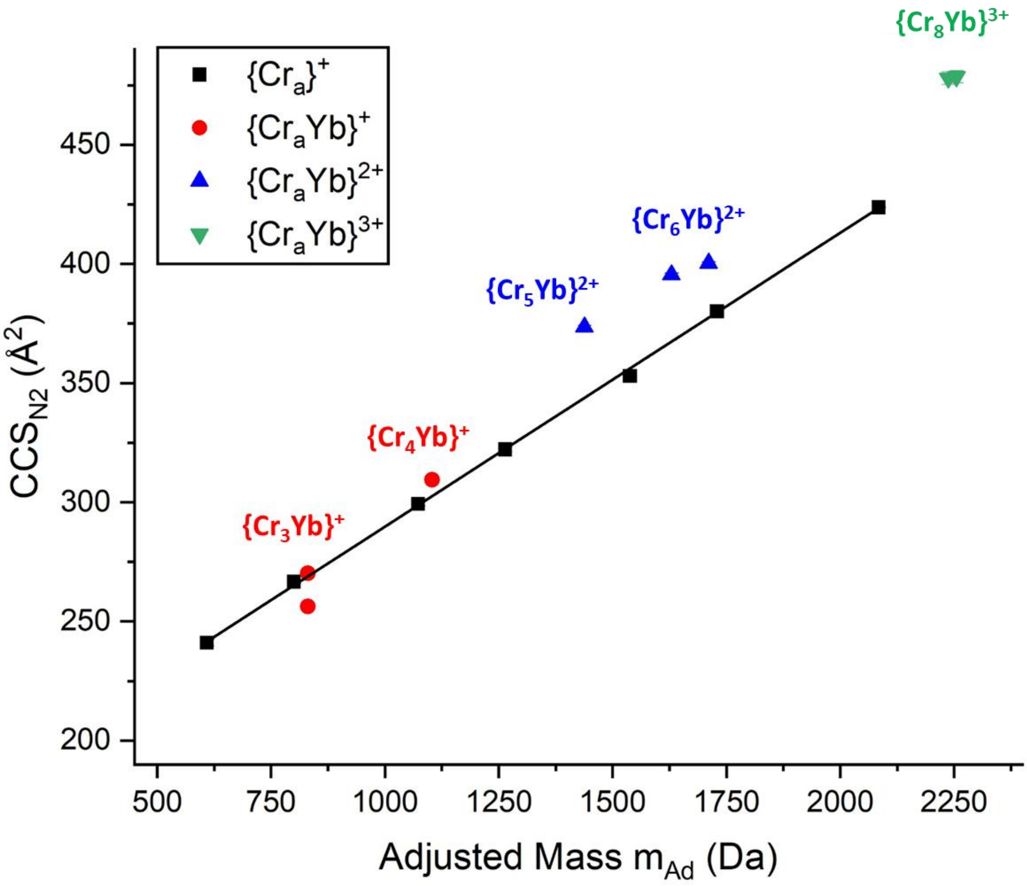

Figure S54: Correlation between CCS_{N2} and adjusted mass for {Cr$_a$Yb} ions. Data for singly charged homometallic ions (Table S5) are represented as black squares, whereas data for heterometallic ions are depicted as red circles (+1), blue upwards triangles (+2), green downwards triangles (+3), respectively (Table S1 for host-guest complexes and Table S6 for fragments). The mass was adjusted so that every metal centre is accounted for with the mass of chromium (52 Da). The species {Cr$_5$Yb}$^{2+}$ and {Cr$_6$Yb}$^{2+}$ were tentatively assigned to open chain structures as they deviate 6 – 9% from the trendline, however it is challenging to disentangle the charge effect reliably.

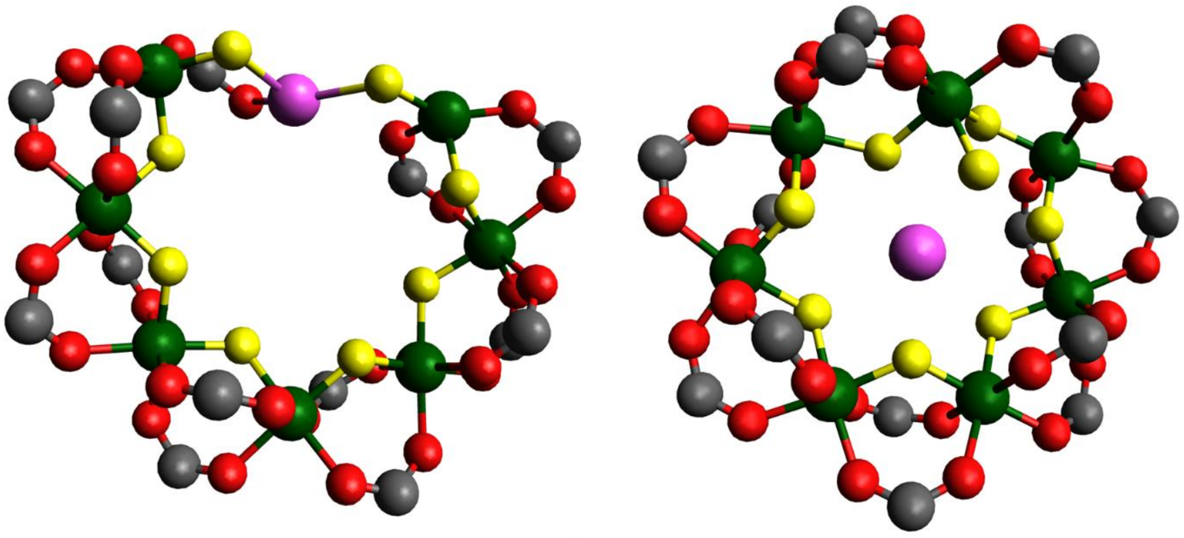

Figure S55: DFT optimised structures of fragment $[Cr_7AgF_8Piv_{13}]^+$ as ring (left) and host-guest complex (right; Ag: pink, F: yellow, Cr: dark green, C: black, O: red). *Tert*-butyl groups were omitted for clarity. Optimised coordinates and ESP charges can be found in the Supplementary Dataset.

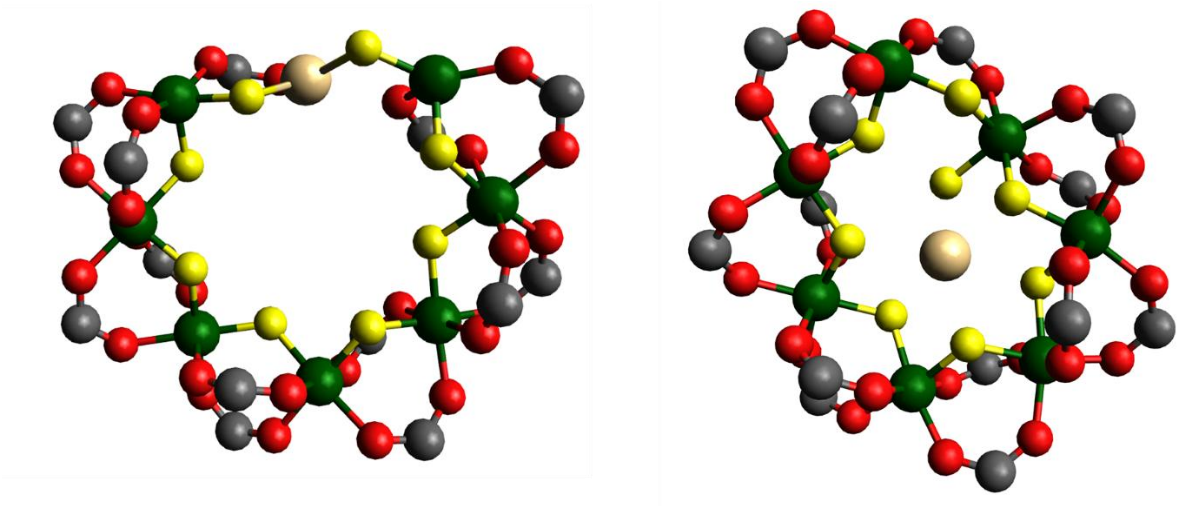

Figure S56: DFT optimised structures of fragment $[Cr_7CdF_8Piv_{13}]^{2+}$ as ring (left) and host-guest complex (right; Cd: gold, F: yellow, Cr: dark green, C: black, O: red). *Tert*-butyl groups were omitted for clarity. Optimised coordinates and ESP charges can be found in the Supplementary Dataset.

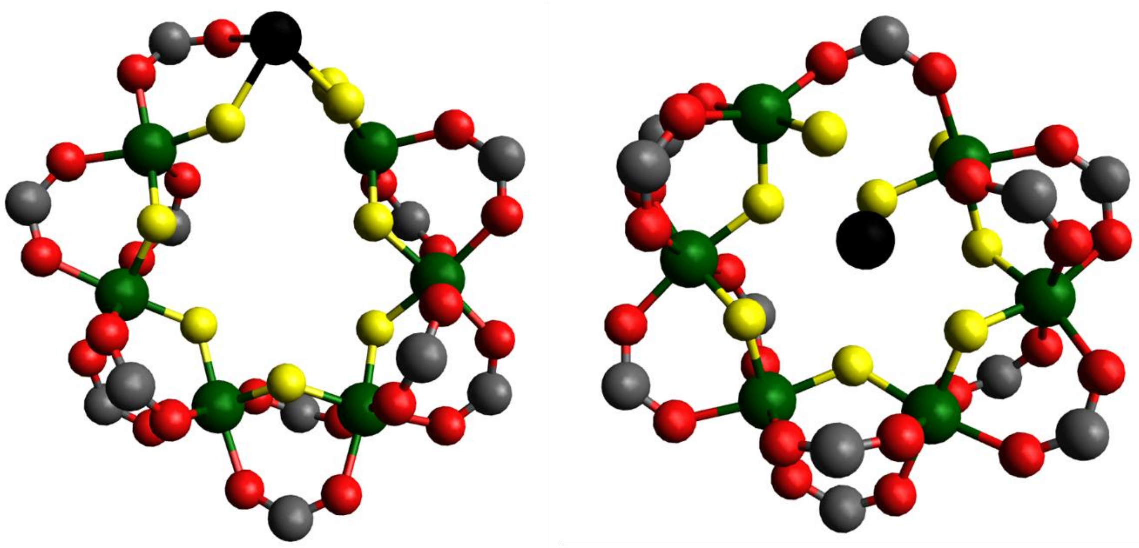

Figure S57: DFT optimised structures of fragment $[Cr_6SnF_8Piv_{11}]^+$ as ring (left) and host-guest complex (right; Sn: black, F: yellow, Cr: dark green, C: black, O: red). *Tert*-butyl groups were omitted for clarity. Optimised coordinates and ESP charges can be found in the Supplementary Dataset.

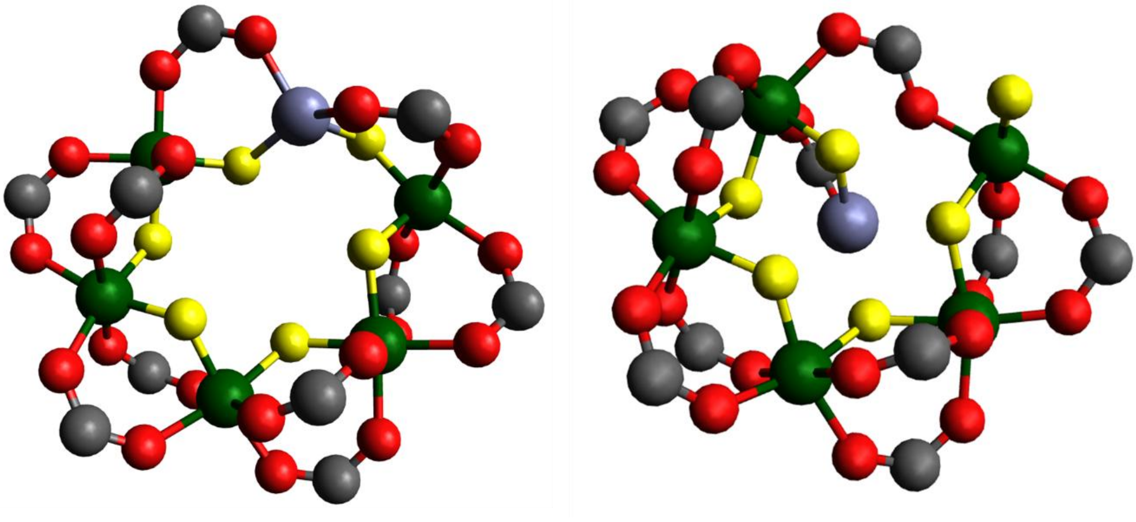

Figure S58: DFT optimised structures of fragment $[Cr_5ZnF_6Piv_{10}]^+$ as ring (left) and host-guest complex (right; Zn: blue grey, F: yellow, Cr: dark green, C: black, O: red). *Tert*-butyl groups were omitted for clarity. Optimised coordinates and ESP charges can be found in the Supplementary Dataset.

Table S7: Unscaled $^{TH}CCS_{N2}$ values of selected fragment ions from Table 2 including error as well as relative minimum energies.

Fragment	Mass (Da)	Unscaled $^{TH}CCS_{N2}$ of the Ring (Å^2)	Relative Energy of the Ring (kJ mol^{-1})	Unscaled $^{TH}CCS_{N2}$ of the Host-Guest Complex (Å^2)	Relative Energy of the Host-Guest Complex (kJ mol^{-1})
$[Cr_7AgF_8Piv_{13}]^+$	1938.3	430.5 ± 0.9	221	421.7 ± 1.2	0
$[Cr_7CdF_8Piv_{13}]^{2+}$	1943.2	444.3 ± 2.4	197	436.1 ± 1.4	0
$[Cr_6SnF_8Piv_{11}]^+$	1695.2	396.9 ± 1.3	0	389.3 ± 1.9	12
$[Cr_5ZnF_6Piv_{10}]^+$	1450.2	360.3 ± 1.2	0	355.0 ± 0.9	116

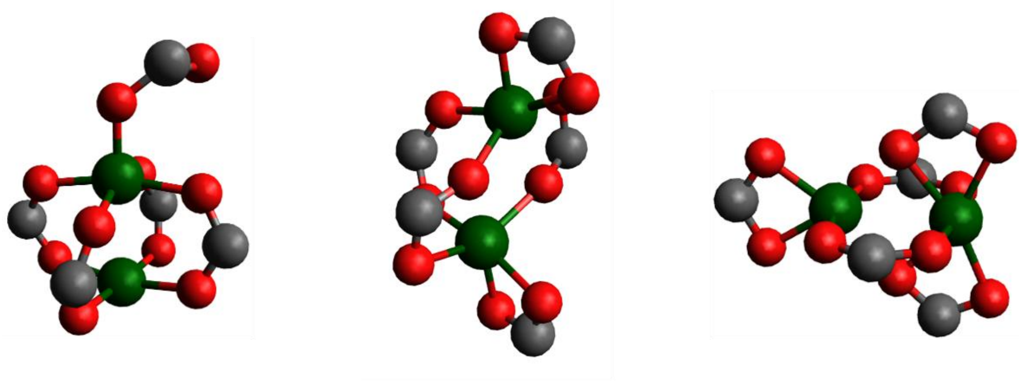

Cr2_crest1 Cr2_crest3 Cr2_CI

Figure S59: DFT optimised structures of fragment $[Cr_2Piv_5]^+$ (Cr: dark green, C: black, O: red). *Tert*-butyl groups were omitted for clarity. Cr2_CI was derived by chemical intuition whereas Cr2_crest1 and Cr2_crest are the result of conformational sampling with CREST. Optimised coordinates and ESP charges can be found in the Supplementary Dataset.

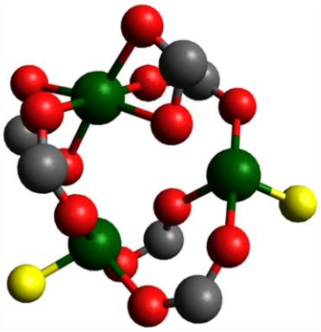

Cr3_crest0

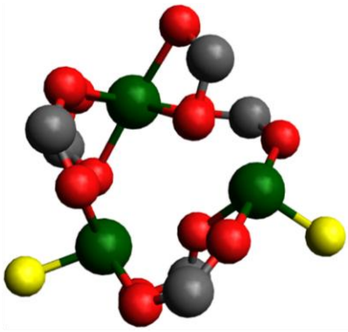

Cr3_crest1

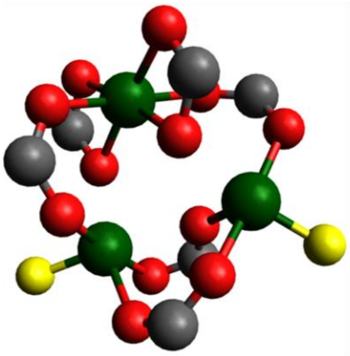

Cr3_crest2

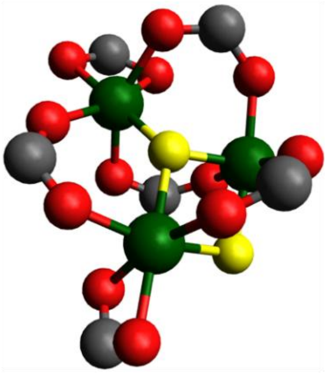

Cr3_CI

Figure S60: DFT optimised structures of fragment $[Cr_3F_2Piv_6]^+$ (Cr: dark green, F: yellow, C: black, O: red). *Tert*-butyl groups were omitted for clarity. Cr3_CI was derived by chemical intuition whereas Cr3_crest0, Cr3_crest1 and Cr3_crest2 are the result of conformational sampling with CREST. Optimised coordinates and ESP charges can be found in the Supplementary Dataset.

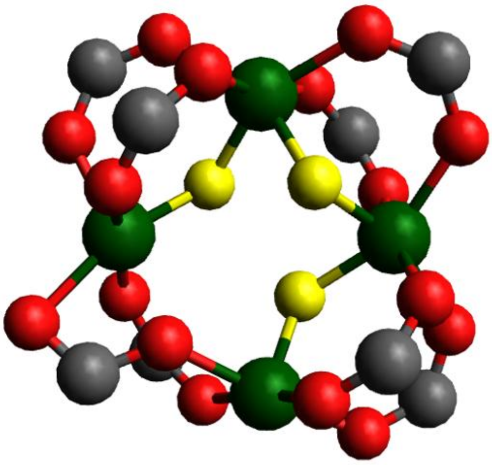

Cr4_CI

Figure S61: DFT optimised structure of fragment $[Cr_4F_3Piv_8]^+$ (Cr: dark green, F: yellow, C: black, O: red). *Tert*-butyl groups were omitted for clarity. Cr4_CI was derived by chemical intuition. Optimised coordinates and ESP charges can be found in the Supplementary Dataset.

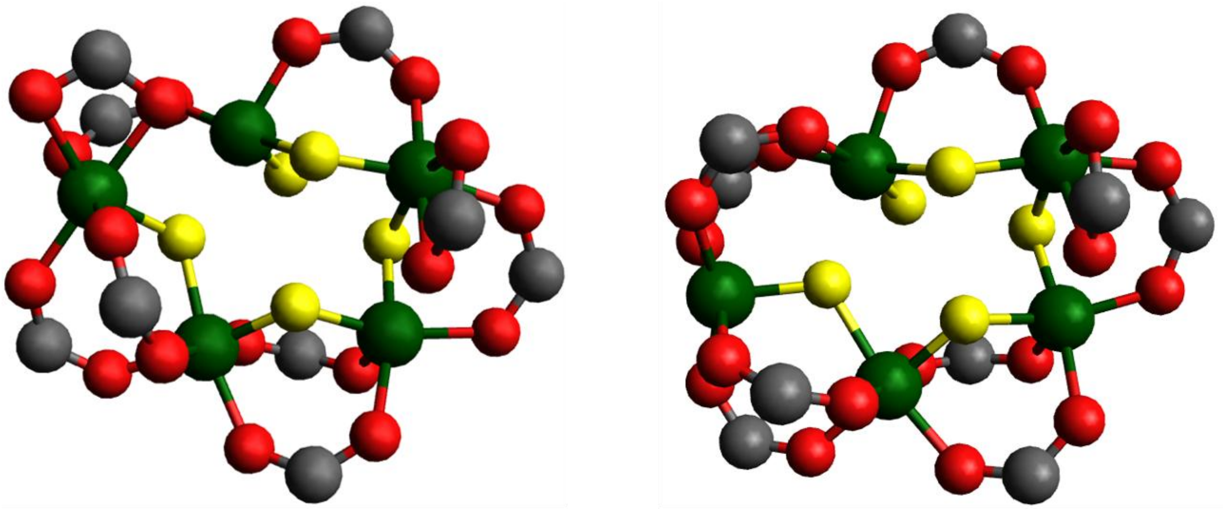

Cr5_CI1 Cr5_CI2

Figure S62: DFT optimised structures of fragment $[Cr_5F_5Piv_9]^+$ (Cr: dark green, F: yellow, C: black, O: red). *Tert*-butyl groups were omitted for clarity. Cr5_CI1 and Cr5_CI2 were derived by chemical intuition. Optimised coordinates and ESP charges can be found in the Supplementary Dataset.

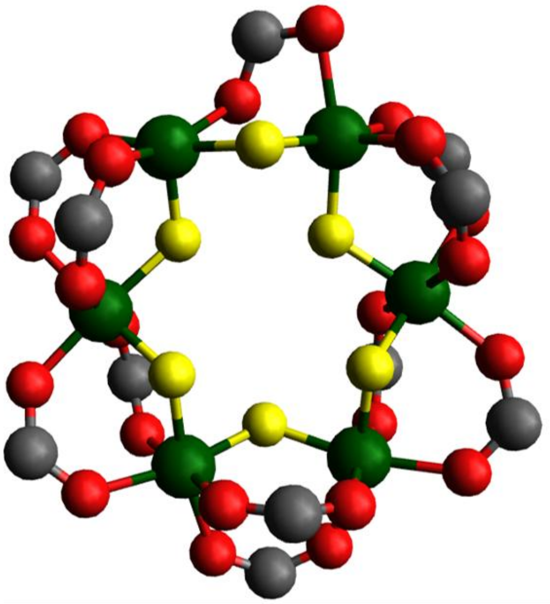

Cr6_CI

Figure S63: DFT optimised structure of fragment $[Cr_6F_6Piv_{11}]^+$ (Cr: dark green, F: yellow, C: black, O: red). *Tert*-butyl groups were omitted for clarity. Cr6_CI was derived by chemical intuition. Optimised coordinates and ESP charges can be found in the Supplementary Dataset.

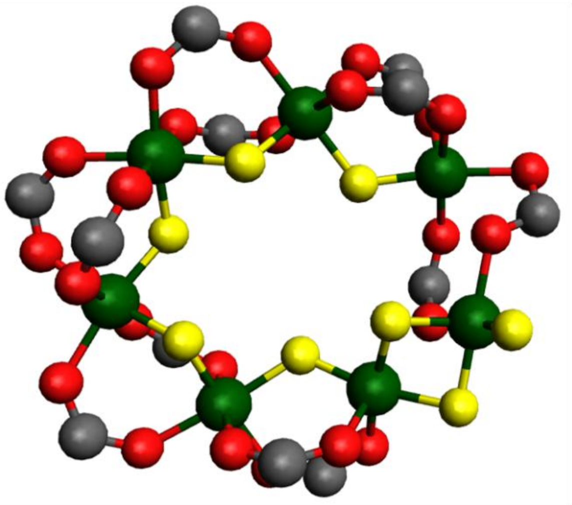

Cr7_CI1

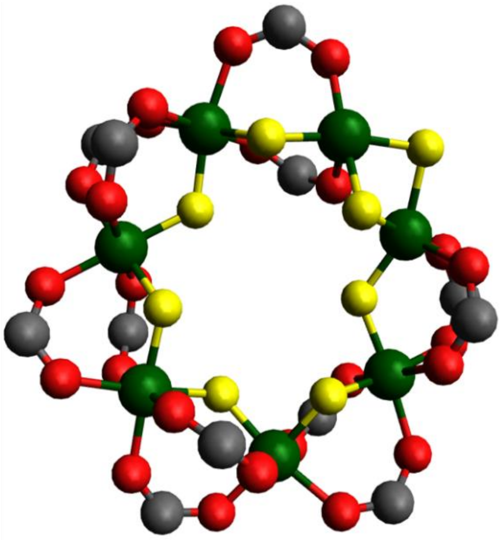

Cr7_CI2

Figure S64: DFT optimised structures of fragment $[Cr_7F_8Piv_{12}]^+$ (Cr: dark green, F: yellow, C: black, O: red). *Tert*-butyl groups were omitted for clarity. Cr7_CI1 and Cr7_CI2 were derived by chemical intuition. Optimised coordinates and ESP charges can be found in the Supplementary Dataset.

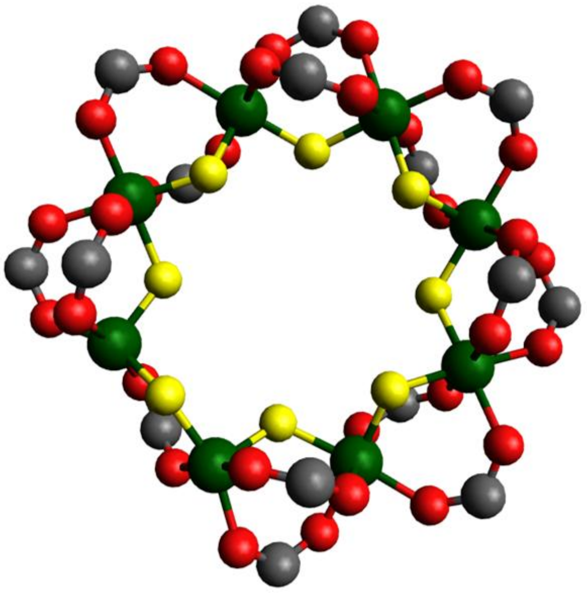

Cr8_CI

Figure S65: DFT optimised structure of fragment $[Cr_8F_8Piv_{15}]^+$ (Cr: dark green, F: yellow, C: black, O: red). *Tert*-butyl groups were omitted for clarity. Cr8_CI was derived by chemical intuition. Optimised coordinates and ESP charges can be found in the Supplementary Dataset.

Table S8: $^{TH}CCS_{N2}$ values and relative minimum energies for singly charged homometallic fragments.

Fragment	Candidate Structure ID	$^{TH}CCS_{N2}$ (Å^2)	Relative Minimum Energy (kJ mol^{-1})
$[Cr_8F_8Piv_{15}]^+$	Cr8_CI	454.0 ± 1.6	-
$[Cr_7F_8Piv_{12}]^+$	Cr7_CI1	400.1 ± 1.9	40
	Cr7_CI2	405.6 ± 0.5	0
$[Cr_6F_6Piv_{11}]^+$	Cr6_CI	379.6 ± 1.5	-
$[Cr_5F_5Piv_9]^+$	Cr5_CI1	336.3 ± 0.7	0
	Cr5_CI2	337.8 ± 0.9	17
$[Cr_4F_3Piv_8]^+$	Cr4_CI	306.0 ± 0.7	-
$[Cr_3F_2Piv_6]^+$	Cr3_crest0	245.7 ± 1.1	0
	Cr3_crest1	246.8 ± 0.7	0
	Cr3_crest2	247.0 ± 0.6	0
	Cr3_CI	264.3 ± 0.5	15
$[Cr_2Piv_5]^+$	Cr2_crest1	225.8 ± 1.0	167
	Cr2_crest3	232.1 ± 0.5	0.0
	Cr2_CI	231.2 ± 1.0	16

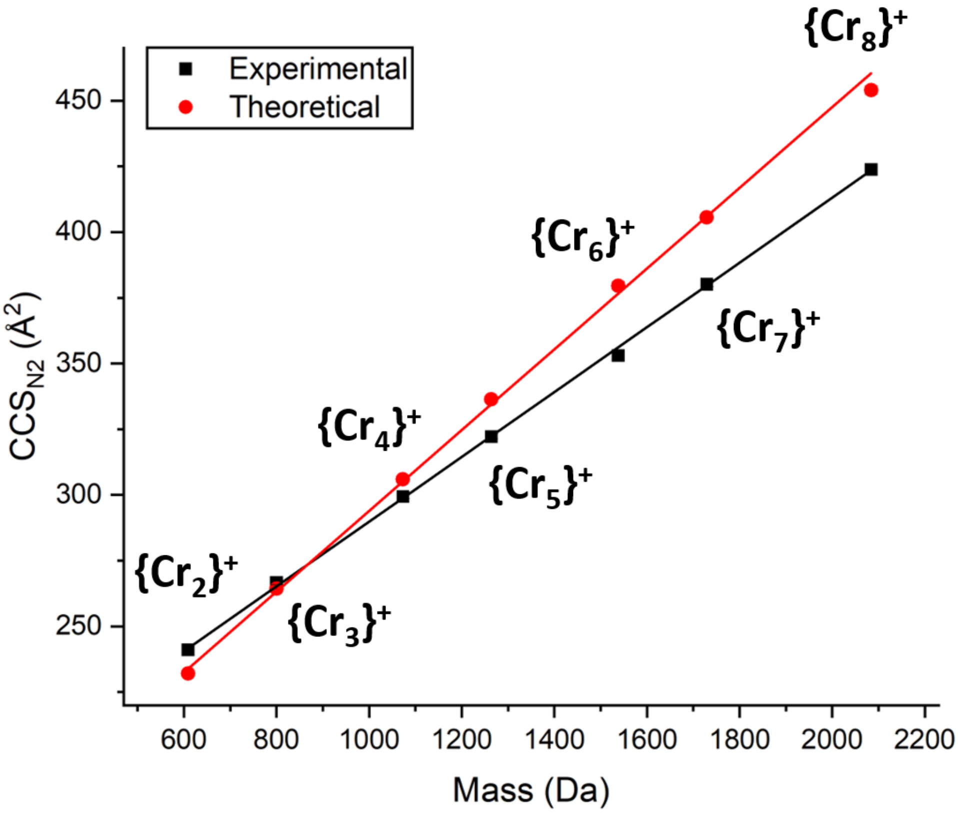

Figure S66: Correlation of experimental (black) and theoretical (red) CCS$_{N2}$ values with mass for {Cr$_a$}$^+$ ions. Both experimental and theoretical data yield linear correlations that show different slopes and intercepts. Experimental and theoretical CCS$_{N2}$ values were used from Tables S5 and S8, respectively.

The comparison of theoretical and experimental CCS$_{N2}$ values of the {Cr$_a$}$^+$ ions yields two lines with different slopes and intercepts. For the theoretical data, different candidate structures were modelled for some of the homometallic ions (Figures S59 – S65), and the lowest energy structure was included in the plot for all cases except for {Cr$_3$}$^+$. For {Cr$_3$}$^+$, the structure Cr3_CI was chosen (relative minimum energy: 14 kJ/mol, Table S8) which is based on chemical intuition, as the more stable structures obtained from CREST are highly unlikely to emerge through fragmentation of the precursor ion. This is because of the location of the fluoride ligands, which in the precursor exclusively bridge metal centres, whereas they are terminal in Cr3_crest0, Cr3_crest1 and Cr3_crest2. Although they cannot be fully excluded, we suggest that Cr3_CI is most likely of the structures sampled and further lies within an acceptable energy difference to the most stable structure. Importantly, the overarching conclusions of

the scaling function are unaffected by the choice of the $\{Cr_3\}^+$ structure, and for the other $\{Cr_a\}^+$ ions, theoretical CCS_{N2} values do not change much for different candidate structures (Table S8).

The mass-dependent scaling function *SF (m_{Ad})* can be obtained through dividing the linear function of the experimental data by the linear function of the theoretical data:

$$SF\ (m_{Ad}) = \frac{slope_{exp} \cdot m_{Ad} + intercept_{exp}}{slope_{theor} \cdot m_{Ad} + intercept_{theor}} = \frac{0.12323\ \frac{\text{Å}^2}{Da} \cdot m_{Ad} + 166.61\ \text{Å}^2}{0.15362\ \frac{\text{Å}^2}{Da} \cdot m_{Ad} + 140.32\ \text{Å}^2}$$

The scaled theoretical CCS_{N2} of the heterometallic fragments can be found in Table 2.

References

(1) Jordan, R. B. Lanthanide Contraction: What Is Normal? *Inorg. Chem.* **2023**, *62* (9), 3715–3721. https://doi.org/10.1021/acs.inorgchem.2c03674.

(2) Geue, N.; Bennett, T. S.; Ramakers, L. A. I.; Timco, G. A.; McInnes, E. J. L.; Burton, N. A.; Armentrout, P. B.; Winpenny, R. E. P.; Barran, P. E. Adduct Ions as Diagnostic Probes of Metallosupramolecular Complexes Using Ion Mobility Mass Spectrometry. *Inorg. Chem.* **2023**, *62* (6), 2672–2679. https://doi.org/10.1021/acs.inorgchem.2c03698.

(3) Shannon, R. D. Revised Effective Ionic Radii and Systematic Studies of Interatomic Distances in Halides and Chalcogenides. *Acta Crystallogr. A* **1976**, *32* (5), 751–767. https://doi.org/10.1107/S0567739476001551.

(4) Irving, H.; Williams, R. J. P. The Stability of Transition-Metal Complexes. *J. Chem. Soc.* **1953**, 3192–3210.
